# Endomyocardial Gremlin-1 is associated with structural remodeling and adverse clinical outcomes in non-ischemic cardiomyopathy

**DOI:** 10.1038/s43856-026-01762-9

**Published:** 2026-07-02

**Authors:** Tobias Harm, Karin Klingel, Patrick Krumm, David Heinzmann, Livia Dingemann, Ioannis Toskas, Peter Seizer, Jörg Kumbrink, Thomas Kirchner, Oliver Borst, Jürgen Schreieck, Tobias Geisler, Simon Greulich, Meinrad Paul Gawaz, Karin Anne Lydia Müller

**Affiliations:** 1 Department of Cardiology and Angiology, University Hospital Tübingen, Eberhard Karls University Tübingen, Tübingen, Germany; 2 Cardiopathology, Institute for Pathology and Neuropathology, University Hospital Tübingen, Tübingen, Germany; 3 Department of Diagnostic and Interventional Radiology, University Hospital Tübingen, Tübingen, Germany; 4 Department of Cardiology, Ostalb Clinic Aalen, Aalen, Germany; 5 Institute of Pathology, University of Munich, Munich, Germany; 6 German Cancer Consortium, German Cancer Research Center, Heidelberg, Germany

**Keywords:** Prognostic markers, Cardiology

## Abstract

**Background:**

Risk stratification in non-ischemic cardiomyopathies (NICM) remains challenging despite guideline-based phenotypic classification using multimodal diagnostics including endomyocardial biopsy (EMB). We aimed to identify EMB-derived histological and molecular markers that improve phenotypic characterization and long-term risk stratification in patients with NICM.

**Methods:**

In this prospective cohort study, 703 consecutive patients with symptomatic NICM underwent standardized multimodal evaluation, including clinical assessment, cardiac imaging, and endomyocardial biopsy. Biopsy specimens were analyzed using histology, immunohistochemistry, and targeted myocardial mRNA profiling. Associations between endomyocardial markers, and fibroinflammatory remodeling, imaging parameters, and molecular signatures were assessed cross-sectionally. Long-term prognostic relevance was evaluated using survival and multivariable prediction analyses during follow-up of up to fifteen years for all-cause mortality, cardiovascular mortality, implantable cardioverter-defibrillator (ICD) implantation, and appropriate ICD discharge.

**Results:**

Elevated myocardial Gremlin-1 expression was associated with increased fibrosis, adverse cardiac remodelling, reduced left ventricular function, and enrichment of pro-fibrotic and inflammatory mRNA signalling pathways. Myocardial and circulating Gremlin-1 expression was independently associated with all-cause and cardiovascular mortality, and ICD implantation and discharge. Machine learning–based phenotyping using histological EMB data identified Gremlin-1 as a key predictive feature of poor prognosis. Incorporation of Gremlin-1 into predictive models significantly improved long-term cardiovascular risk stratification in NICM patients.

**Conclusion:**

Our results unveil that Gremlin-1 is associated with inflammation and cardiac remodelling in patients with NICM, and patients with Gremlin-1^+^ EMB and high plasmatic Gremlin-1 concentrations are at elevated risk to develop adverse cardiovascular events. Thus, the histological evaluation of Gremlin-1 may help to improve risk discrimination and management of NICM and HF patients.

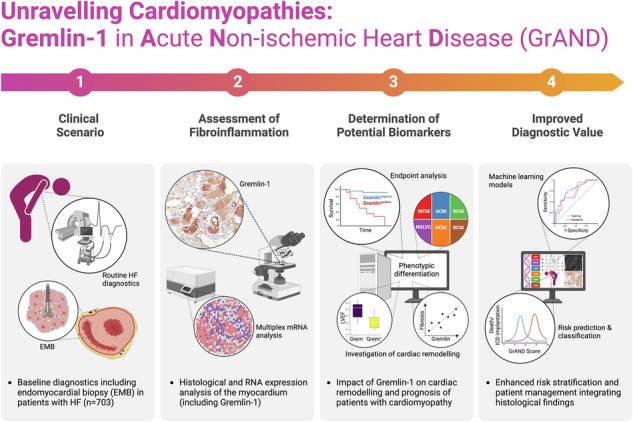

## Plain Language Summary

Non-ischemic cardiomyopathy (NICM) refers to diseases in which the heart muscle becomes abnormal and unable to pump effectively, without being caused by blocked coronary arteries. Predicting which patients will develop serious complications remains difficult using current clinical tests. We examined whether information from small heart tissue samples could improve long-term risk prediction. We analysed 703 NICM patients who underwent comprehensive clinical assessment and endomyocardial biopsy. Heart tissue was assessed for markers of inflammation and scarring, including the protein Gremlin-1, and patients were followed for up to fifteen years for major cardiovascular outcomes. Higher Gremlin-1 expression and circulating Gremlin-1 were associated with scarring, reduced heart function, and increased risk of adverse outcomes. These findings suggest that tissue-based biomarkers including Gremlin-1 may support more accurate risk stratification and personalized management in NICM.

## Abstract (web summary)

Harm et al. analyse endomyocardial biopsies from 703 patients with non-ischemic cardiomyopathy to test whether tissue-based markers improve long-term risk prediction. Increased myocardial Gremlin-1 is associated with adverse remodelling and predicts overall and cardiovascular mortality, defibrillator implantation, and appropriate discharge.

## Introduction

Cardiomyopathies represent a significant cause of progressive heart failure (HF) among adults, imposing a considerable socioeconomic burden^1,2^. Hitherto, management of patients with cardiomyopathy remains challenging since the course of the disease spans from complete recovery to repeated hospitalisation and end-stage HF necessitating heart transplantation^3^. Significant advances in both pharmacological and device-based therapies have remarkably enhanced cardiovascular outcomes over the past decades^3,4^.

Persistent inflammation, immune activation, and fibrotic cardiac remodelling are key drivers of disease progression and adverse outcomes, including cardiac death, in both ischemic and non-ischemic cardiomyopathies^5–7^. Recent advances in histological analyses of endomyocardial biopsy (EMB) enabled to characterize aberrant structural changes of the myocardium in patients with underlying cardiomyopathy.

We and others found that assessment of histological and immunohistochemical signatures, such as expression of cytokine and pro-inflammatory mediators, infiltration of immune cells, and cardiac fibrosis, independently determines the progression of HF^8–11^. Previously, critical changes in the endomyocardial tissue contributing to the pathophysiology of inflammatory cardiomyopathies were found to differentiate the aetiology on NICM^7,12,13^. While crucial for treatment strategies and prognosis, current guidelines prioritize an integrated phenotypic classification system over an etiological one as the conceptual framework for diagnostics and therapies in patients with cardiomyopathy^3^. A bridging between both phenotypic traits and underlying pathophysiological determinants might therefore facilitate diagnostics and improve the prognosis of patients with cardiomyopathies.

In this study, we performed a comprehensive risk assessment, including endomyocardial biopsy, in a large prospective cohort of patients with symptomatic heart failure (*n* = 703). We profiled structural, histological, and myocardial mRNA expression changes alongside guideline-based multimodal clinical assessment and cardiac imaging of cardiomyopathies. The primary objective was to determine whether EMB-derived histological and molecular features capture disease-relevant pathophysiological mechanisms in non-ischemic cardiomyopathies and can (i) support guideline-based phenotypic classification and (ii) enable clinically meaningful risk stratification. To this end, we applied machine learning approaches integrating histopathological data with long-term clinical follow-up of up to ten years. We demonstrate that myocardial Gremlin-1 is associated with adverse remodeling and independently predicts adverse cardiovascular outcomes.

## Methods

### Study population

This study was designed to establish a prospective cohort of patients with symptomatic NICM to enable integrated phenotypic characterization and long-term outcome assessment. Therefore, seven hundred three (703) patients with new onset of symptomatic non-ischemic HF were enrolled in this prospective, consecutive study at the university hospital of Tübingen.

Inclusion criteria comprised clinical presentation with HF symptoms, and structural and/ or functional cardiac abnormalities according to ESC guideline definitions^3,14–16^, irrespective of underlying etiology, including patients in whom subsequent EMB identified non-ischemic cardiomyopathy or, in a minority of cases, biopsy-proven cardiac allograft rejection. A careful clinical follow-up of CV events was initially performed over a mean 3.5-year period after hospital discharge and relied on a review of medical records from hospital documentation or general practitioners. The primary endpoint of all-cause mortality and eligibility for ICD implantation was selected to reflect clinically relevant disease progression and guideline-based risk stratification in patients with NICM. Further, key secondary endpoints included cardiovascular mortality and appropriate ICD discharge to assess the risk for sudden cardiac death, and were assessed according to guideline-based recommendations during the clinical follow-up period. We additionally screened for all-cause mortality and appropriate ICD discharge during an extended follow-up of up to fifteen years (mean 7.4 years) to elucidate the impact of plasmatic Gremlin-1 on adverse outcomes. For the investigation of non-failing hearts, post-mortem myocardial samples were obtained from 20 individuals who had died from non-cardiac causes. All individuals had a documented normal left ventricular ejection fraction and no history of cardiovascular disease or treatment with antihypertensive or heart failure medications. Comprehensive review of medical history, clinical records, and pathological examination revealed no evidence of structural heart disease in any donor (Suppl. Table S1). The study design was approved by the local ethics committee of Tübingen (253/2009B02 and 240/2018B02), and written informed consent was obtained from all patients. The study was conducted in accordance with the highest ethical standards as laid down within the Declaration of Helsinki.

### Phenotypic classification of cardiomyopathies

NICM phenotypes were defined using a guideline-based multimodal diagnostic framework to provide the clinical reference for subsequent histological and molecular analyses. The definition of NICM was based on the phenotypic classification approach of the current guidelines proposed by the European Society of Cardiology^3,15^. Therefore, a multimodal diagnostics algorithm integrated the aetiology and clinical scenario (e.g., symptoms, ECG analysis, family screening), a morphological (e.g., dilatation, hypertrophy, ventricular scar, fatty replacement), and functional characterization (global and regional wall movement, ventricular ejection fraction), and assessment of additional traits (e.g., histopathology, cardiac magnetic resonance (CMR) imaging, genetic testing, laboratory markers, extracardiac involvement) in patients with suspected cardiomyopathy.

In accordance with the definition of cardiomyopathies, patients with significant coronary artery disease (CAD), valvular, hypertensive, or congenital heart disease were excluded from this study^3,17^. Therefore, coronary angiography was performed in all patients to rule out relevant CAD ( > 50% diameter luminal stenosis). Pathological analysis was performed in all patients by a trained cardiopathologist at the University Hospital of Tübingen and included histological and immunohistochemical evaluation in relation to the phenotypic classification workup. Electron microscopy was employed in cases of suspected storage or mitochondrial cardiomyopathies.

Routine echocardiography (iE33, Philips Medical Systems, Hamburg, Germany) included assessment of left ventricular ejection fraction (LVEF) and left ventricular end-diastolic diameter (LVEDD) in all patients. LVEF was analysed from apical four-chamber and two-chamber views using the modified Simpson method. LVEDD was quantified by two-dimensionally guided motion mode echocardiography.

Left ventricular functional capacity and morphology were assessed by contrast-enhanced CMR using a 1.5 T scanner (Siemens Medical Systems, Erlangen, Germany). A breath-holding manoeuvre was performed to standardize acquisition of ECG-gated images with the subject in the supine position. For acquisition of late gadolinium enhancement (LGE) patters, a two-dimensional segmented inversion-recovery fast gradient echo was performed 5–10 min after contrast administration. The contrast dose was 0.15 mmol gadobutrol per kilogram of body weight (Gadovist, Bayer Healthcare, Berlin, Germany).

CMR imaging was conducted and analysed using an advanced cardiac software package and independently reviewed in a blinded manner by two experienced investigators as outlined previously^18^. Thereafter, HF patients were categorized based preserved (HFpEF; LVEF ≥ 50%) mildly reduced (HFmrEF; LVEF 41–49%) or reduced (HFrEF; LVEF ≤ 40%) LVEF. All patients enrolled into this prospective study underwent an extensive clinical examination and standardized questionnaire on medication history, smoking status, genetic disposition, and contact information to conduct a careful clinical follow-up analysis. Risk factors were assessed according to validated standard procedures as recommended by current guidelines^19–21^. In patients with only assessment of B-type natriuretic peptide (BNP), a validated conversion was performed to obtain N-terminal pro BNP values^22^.

All patients received guideline-based treatment including heart failure medication according to recommendations of the European Society of Cardiology at study entrance and after performance of EMB to mitigate potential bias from co-medication on histological findings^14,15,23^. Adherence to optimal medical therapy (OMT) was monitored during regular visits to our outpatient clinic and through contact with healthcare practitioners. A follow-up of patients included in this study was performed over a maximum ten-year period after hospital dismissal. The close-meshed clinical follow-up relied on review of medical data including general practitioners, and visits from our outpatient clinics. Analyses were conducted according to the intention-to-treat principle. All-cause mortality included non-cardiac and cardiac death. The latter included sudden cardiac death (SCD) and aborted SCD, defined as resuscitation after cardiac arrest or appropriate defibrillator discharge following ventricular arrhythmia. Eligibility for ICD implantation was based on recommendations endorsed by the European Society of Cardiology^15,24^.

### Specific classification of non-ischemic cardiomyopathies (NICM)

Phenotypic classification was further refined by integrating clinical, imaging, and histopathological data to link structural characteristics with underlying disease mechanisms. NICM were defined based on structurally and functionally abnormal myocardium. The diagnostic algorithm to further refine the classification of cardiomyopathies integrated the clinical scenario (i.e., symptoms, family history), functional and morphological characterisation assessed by cardiac imaging, as well as additional traits (i.e., findings from EMB, ECG, genetic testing, or laboratory parameters)^3,25–27^. For the phenotypic classification echocardiography parameters were determined by retrospective analyses of DICOM-data of transthoracic echocardiographic records in all patients and cardiac magnetic resonance imaging in 384 patients.

In adults, hypertrophic cardiomyopathy (HCM) was diagnosed when the left ventricular (LV) wall thickness was ≥15 mm in any myocardial segment, and the thickening could not be explained solely by loading conditions^3^. In first-degree relatives of individuals with definitive HCM, the condition was identified by an LV wall thickness of ≥ 13 mm^3^. MRI was performed to screen for patchy mid-wall LGE pattern in areas of hypertrophy and at the anterior and posterior RV insertion points^3^. Histopathological findings in HCM included myocyte hypertrophy and disarray, interstitial or pericellular-type fibrosis, and endocardial fibrosis^28^. Dilated cardiomyopathy (DCM) was characterized by left ventricular (LV) dilatation and systolic dysfunction that cannot be fully explained by abnormal loading conditions^3^. LV dilatation was defined as an LV end-diastolic diameter (LVEDD) ≥ 58 mm in males and ≥ 52 mm in females. Global systolic LV dysfunction was defined by LVEF of ≤ 50%^3^. MRI findings in DCM included myocardial edema and LGE, suggesting specific etiologies (e.g, subepicardial distribution indicates post-myocarditis, patchy LGE suggest sarcoidosis, inferolateral involvement in dystrophinopathies, and septal mid-wall enhancement in hereditary forms)^3^. To determine the underlying etiology, inflammatory cell infiltration, myocardial disarray, vacuole formation, cardiomyocyte hypertrophy, myocytolysis, scar formation, collagen deposition, and fibrosis, granulomas and giant cells were assessed by EMB^3^. The non-dilated left ventricular cardiomyopathy (NDLVC) phenotype was defined by non-ischemic LV scarring or fatty replacement without LV dilatation, or by isolated global LV hypokinesia without scarring, as indicated by the presence of LGE on cardiac MRI. EMB allowed for further etiological classification, including mostly inflammatory heart disease^3^. Arrhythmogenic cardiomyopathy (ACM) diagnosis was based on the revised 2010 Task force criteria^29^ alongside the Padua criteria^30^. Cardiac imaging including transthoracic echocardiography and cardiac MRI, was performed to detect LV or RV myocardial atrophy with fibro-fatty replacement of the RV myocardium corresponding with wall motional abnormalities such as ventricular akinesia, dyskinesia, bulging, or dilatation. Further, the diagnostic workup included ECG, Holter monitoring, genetic testing, and EMB^3^. In the latter, fibro-fatty replacement and myocyte atrophy of the ventricular myocardium were the most prominent features^31^. A restrictive phenotype of cardiomyopathies (RCM) was defined by diastolic dysfunction and normal systolic LV function^3^. Genetic testing was performed to identify primary RCM and genetic variants of cytoskeletal proteins^32^. Cardiac MRI was performed to distinguish RCM from constrictive pericarditis, and to provide the extent of myocardial fibrosis, which distinguishes metabolic from inflammatory diseases^3^. EMB was performed to detect further aetiology of restrictive heart disease (e.g., endomyocardial fibrosis, amyloidosis, hypereosinophilia, hyperoxaluria, and post-radiation fibrosis)^33,34^. Patients were diagnosed with mixed phenotype cardiomyopathy (MCM) if findings from the diagnostic workup, including cardiac imaging, endomyocardial biopsy (EMB), genetic testing, and etiologic classification, did not meet the criteria for a specific phenotype or showed overlapping features that could not be attributed to a single phenotype.

### Endomyocardial biopsy

EMB was performed to enable direct assessment of myocardial structure and biology, thereby capturing disease-relevant pathophysiological processes. EMBs were obtained from five different regions of the right ventricular septum using a bioptome (Biopsy Forceps, Cordis Corporation, USA). Endomyocardial biopsy, immunohistological and mRNA analyses, and detection of viral genomes were performed as previously described^3,4,14,15,24,35^. The sample size was calculated based on 15 percentage points difference in mortality rates among patients with aberrant EMB findings. This study was conducted as an intention-to-treat analysis in an all-comers cohort. Assuming an error rate of 10% due to inadequate tissue quality of endomyocardial biopsy (EMB) specimens, a minimum of 544 biopsies was required. Owing to the availability and sufficient quality of EMB material, histopathological analyses, including fibroinflammatory parameters, were ultimately performed in 633 EMB samples. We analysed the cellular constituents of deparaffinized cardiac tissue sections from endomyocardial biopsies as described previously^9,10,36–38^. Concisely, three samples were fixed in 4% buffered formaldehyde for further immunohistological analysis. Two tissue samples were fixed in RNAlater (Ambion Inc., Foster City, USA) for multiplex mRNA analysis. Immunohistologically stained heart tissue sections were analysed in a semiquantitative approach ranging from 1 (no expression) to 4 (ubiquitous high expression). The analyses were performed by two independent co-investigators in a blinded manner. Subsequently, EMB sections were dichotomized as negative (no/low expression) or positive (moderate/high expression) as described previously (Suppl. Fig. S1)^17,26,39^. Biopsies were treated with an avidin–biotin–immunoperoxidase method (Vectastain-Elite ABC Kit, Vector, Burlingame, USA) and stained with antibodies against C3aR (Santa Cruz Biotechnology, Santa Cruz, USA), C5aR (Abcam, Cambrigde, UK), CD3 (Novocastra Laboratories, Newcastle, UK), CD68 (DAKO, Glostrup, Denmark), CypA (Abcam, Cambrigde, UK), CXCL12 (Abcam, Cambrigde, UK), Emmprin (Abcam, Cambrigde, UK), Gremlin-1 (Abnova, Taipeh, Taiwan), HLA-Drα/MHCII (DAKO, Hamburg, Germany), and MIF (R&D Systems, Minneapolis, USA).

### Immunohistochemistry-photography and analysis

Immunohistochemical analyses were conducted to quantify myocardial inflammatory and fibroinflammatory markers associated with cardiac remodeling. In accordance with histological guidelines, analysis of immunohistochemistry and cardiac fibrosis was blinded to clinical findings^40,41^. The immunostained cardiac tissue sections were photodocumented (Nikon Eclipse Ti2-A, Nikon, Tokyo, Japan) and analysed with standardized protocols and software (NIS-Elements AR, Nikon, Japan). To statistically analyze and compare the quantity of immunohistochemical expression (e.g., C3aR, C5aR, CD3, CD68, CypA, CXCL12, Emmprin, Gremlin-1, MHCII, and MIF), tissue sections were analysed using a score-based approach, ranging from no expression to ubiquitous high expression. Patients were then dichotomized according to expression levels into no/low expression (EMB^-^) and moderate/high expression (EMB^+^), as described previously (Suppl. Fig. S1)^37,41,42^.

Similarly, the amount of fibrosis was analysed in percentage of fibrosis in relation to the total area cardiac tissue sections. Thereafter, patients were categorized according to tertile distribution, defined as mild (0–10%), moderate (11–20%), or severe fibrosis ( >20%).

### Multiplex expression analysis of myocardial mRNA

Targeted myocardial mRNA profiling was performed to characterize molecular pathways underlying fibroinflammatory remodeling linked to adverse outcomes. Total RNA was extracted from up to 15 later-fixed endomyocardial biopsy sections using the RNeasy FFPE Kit (Qiagen, Hilden, Germany) according to the manufacturer’s instructions. RNA yield and purity were assessed using a NanoDrop ND-1000 spectrophotometer (NanoDrop Technologies, Rockland, USA). Targeted messenger RNA (mRNA) expression profiling was performed using the NanoString^®^ nCounter assay (NanoString Technologies, Seattle, USA) with 100 ng of total RNA, as described previously^43^. Data were analysed using NanoString nSolver Software v3.0. Quality control was conducted according to the manufacturer’s guidelines using positive (POS) and negative (NEG) controls, housekeeping genes, and total counts (excluding controls) for each sample. A complete list of analysed transcripts is provided in Suppl. data 1.

### Detection of viral genome

Viral genome analysis was performed to assess potential infectious contributors to myocardial inflammation and adverse remodeling. For detection of viral genomes, deep-frozen or RNAlater-fixed endomyocardial biopsy specimens were analysed using established nested PCR/RT-PCR protocols, as previously described in refs. ^36,42^. The detection included enterovirus species (including coxsackieviruses and echoviruses), adenoviruses, Epstein–Barr virus, human herpesvirus type 6, and parvovirus B19. For RT-PCR analyses, RNA was reverse-transcribed into cDNA. Enzymatic amplification of cDNA or DNA was performed as nested PCR using two 30-cycle programs. Successful nucleic acid isolation was verified by PCR detection of the housekeeping gene GAPDH. Viral detection was categorized qualitatively as positive or negative based on genome detection by PCR, and specificity was confirmed by automatic DNA sequencing of amplification products. For parvovirus B19, additional quantitative real-time PCR analysis was performed to determine viral load, with a lowest detectable copy number of 63 copies/µg myocardial DNA. All PCR procedures included appropriate negative controls and were performed under strict contamination-controlled conditions to minimize the risk of false-positive results.

### Plasma Gremlin-1 measurement

Peripheral venous blood samples were obtained under standardized conditions at the time of clinical evaluation. EDTA-Blood was collected and processed within a defined time frame to minimize pre-analytical variability. Following centrifugation, plasma was separated and stored at −80 °C until analysis. Plasma Gremlin-1 concentrations were quantified using a commercially available enzyme-linked immunosorbent assay (ELISA; USCN Life Science Inc., Houston, TX, USA) according to the manufacturer’s instructions. All samples were measured in duplicate, and mean values were used for analysis. Calibration curves were generated using standard solutions provided in the assay kit, and concentrations were calculated accordingly. Quality control procedures included the use of internal controls and assessment of intra- and inter-assay variability. For the assessment of circulating Gremlin-1 levels, a control cohort of healthy individuals (*n* = 75) was included. All controls had no history of cardiovascular disease, exhibited normal cardiac function, and were not receiving any cardiovascular medication. Laboratory personnel were blinded to clinical data.

### Statistics and Reproducibility

Statistical analyses were performed to identify key predictors and develop models supporting phenotypic classification and risk stratification of NICM. Patients baseline characteristics, histological data, and pre-processed Nanostring^®^ data were analysed using JMP Pro (SAS Institute, Cary, USA) and R (R foundation for Statistical Computing, Vienna, Austria). Non-normally distributed baseline characteristics and histological data were compared using Wilcoxon test and normally distributed data were analysed using student´s t-test and analysis of variance (ANOVA) for multiple subgroups. For multiple group comparison Tukey´s and Dunn´s post-hoc procedures were adopted to correct significance levels of individual tests. Continuous data are presented as median with the lower and upper quartiles or mean and 95% confidence interval (95% CI) as appropriate. Categorical variables are expressed as counts and proportions after comparison with chi-squared test. Correlation data is presented as Spearman’s rank correlation coefficient or Pearson’s correlation coefficient where applicable. RNA expression levels were compared with ANOVA and to account for the multiple hypothesis testing, a false discovery rate (FDR) controlling procedure was further adopted to correct significance levels (*p* < 0.05) for FDR ≤ 5%. Pathway enrichment analysis of RNA data was performed within the “pathfindR” package in R^44^. Therefore, KEGG (Kyoto Encyclopedia of Genes and Genomes) pathway analysis was performed integrating significantly enriched transcripts (Bonferroni *p* < 0.01) within greedy algorithm subnetwork search. Chord chart of RNA data was created with the “circlize” package in R^45^. For prediction of guidelines-based classification of cardiomyopathies, extreme gradient boosting (XGBoost) with cross-validation was applied exclusively integrating histological data. Thus, the patient cohort was randomly split into training dataset (75%, *n* = 527) or test dataset (25%, *n* = 176). Model development included only clinically relevant EMB-derived variables available at the time of prediction and hyperparameters were autotuned. No explicit feature selection was performed, and feature relevance was determined implicitly through XGBoost’s built-in regularization and tree-based splitting mechanisms. Feature importance was assessed using gain, and variables with a gain ≥ 1% were visualized (Suppl. Table S2). For prediction of cardiovascular (CV) outcomes, variable selection was performed using L1-regularized Cox proportional hazards regression with the least absolute shrinkage and selection operator (LASSO), as previously described in ref. ^46^. Optimal hyperparameters were selected using 10-fold cross-validation (Suppl. Fig. S7). For CV risk prediction, LASSO was adjusted for age, gender, and phenotype. CV risk factors sharing a nonzero coefficient from different random data partitions with optimal λ were included for further analysis of the predictive risk. The final models were trained on training data set and cross-validated via test group of patients. Confidence intervals were derived from percentile bootstrap (100 repetitions) analysis. The prediction formula to estimate the risk for all-cause mortality of the entire cohort derived from LASSO was transformed to a likelihood ratio (GrAND Score). Survival analyses for the overall cohort in Kaplan-Meier transformations were conducted using the log-rank test, and subgroups analyses were formed using the likelihood ratio test. To determine thresholds of plasmatic Gremlin-1 a NICM diagnostic threshold was derived from receiver operating characteristic (ROC) analysis comparing patients with non-ischemic cardiomyopathy and healthy controls. For prognostic analyses, a separate high-risk prognostic threshold was determined using time-dependent ROC analysis and the Youden index. No imputation of missing data was performed to avoid introducing model-based assumptions. All patients were retained in the cohort according to the intention-to-treat principle. Analyses were conducted using an available-case approach, with sample sizes varying according to data availability and tissue quality for specific assessments (e.g., immunohistochemistry, mRNA profiling, and cardiac imaging), reflecting the real-world workflow of endomyocardial biopsy–based diagnostics. The number of patients included in each analysis is reported in Suppl. Table S9 and in the corresponding figures and tables.

## Results

### Phenotypic and histological characterisation in non-ischemic cardiomyopathies

**Fig. 1:**
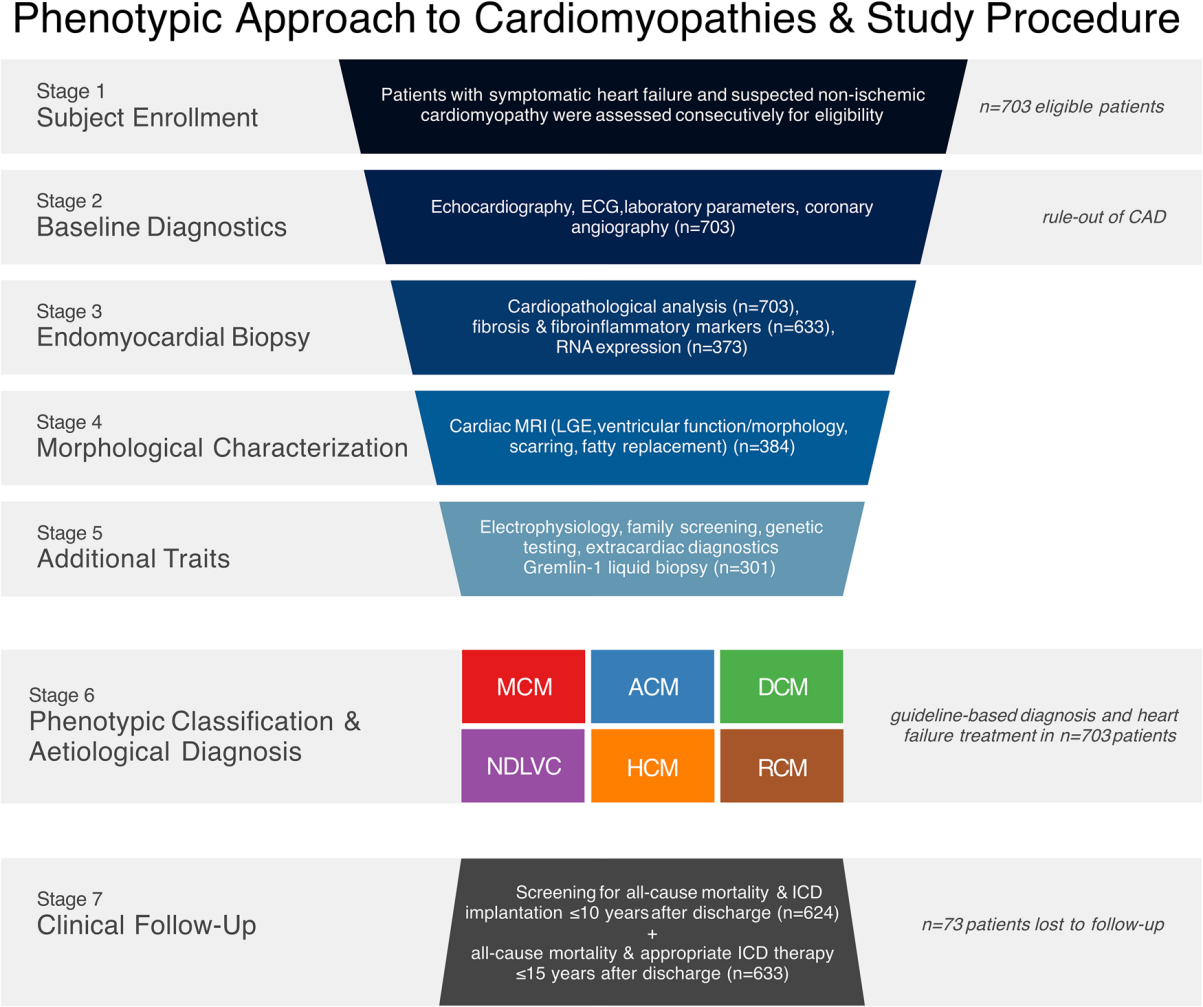
Study protocol and phenotypic classification algorithm of patients with non-ischemic cardiomyopathies (NICM). Patients with symptomatic heart failure due to NICM were consecutively enrolled in an all-comers cohort (*n* = 703). Relevant coronary artery disease (CAD) was ruled-out by coronary angiography and all patients underwent EMB including assessment of fibroinflammation and RNA data. Alongside cardiac imaging, multimodal etiological diagnostics were integrated into the phenotypic classification approach. Thereafter, an up to ten-year follow-up was performed to screen for all-cause mortality and ICD implantation.

In this study, we analysed myocardial abnormalities and mRNA expression via EMB in a large cohort of patients with non-ischemic cardiomyopathy (NICM; *n* = 703) (Fig. 1). Baseline demographics and patient characteristics are presented in Tables 1 and 2. The median age was 59 (58.7–61.1) years, with 26% female patients.

**Table 1:** Baseline characteristics of patient population (*n* = 703)

	All patients (*n* = 703)	MCM (*n* = 95, 13.5%)	ACM (*n* = 11, 1.6%)	DCM (*n* = 246, 35%)	NDLVC (*n* = 275, 39.1%)	HCM (*n* = 23, 3.3%)	RCM (*n* = 53, 7.5%)	*p*-Value
Female, *n* (%)	183 (26)	24 (25.3)	3 (27.3)	48 (19.5)	89 (32.4)	7 (30.4)	12 (22.6)	**0.037**
Age, years (mean, 95% CI)	59 (58.7–61.1)	63.4 (60.3–66.4)	46.9 (39.9–55.9)	60.2 (58.3–62.1)	56.7 (54.9–58.5)	52.8 (46.6–59)	74.3 (70.2–78.4)	**< 0.0001**
Body mass index (mean, 95% CI)	27.1 (26.4–27.8)	28.4 (26.6–30.3)	23.7 (18.3–29)	28.3 (27.1–29.5)	26.3 (25.2–27.4)	27.1 (23.5–30.7)	24.8 (22.4–27.2)	**0.028**
**Medication on admission**								
ACE inhibitors, *n* (%)	271 (38.6)	42 (44.2)	4 (36.4)	105 (42.7)	86 (31.3)	7 (30.4)	27 (50.9)	**0.021**
AT II receptor antagonists, *n* (%)	92 (13.1)	16 (16.8)	0 (0)	28 (11.4)	34 (12.4)	6 (26.1)	8 (15.1)	0.207
Aldosterone antagonists, *n* (%)	201 (28.6)	38 (40)	2 (18.2)	89 (36.2)	52 (18.9)	5 (21.7)	15 (28.3)	**< 0.0001**
β-blockers, *n* (%)	360 (51.2)	62 (65.3)	6 (54.6)	131 (53.3)	117 (42.6)	14 (60.9)	30 (56.6)	**0.003**
Diuretics, *n* (%)	245 (34.9)	47 (49.5)	2 (18.2)	88 (35.8)	73 (26.6)	8 (34.8)	27 (50.9)	**0.001**
ASA, *n* (%)	35 (36.8)	35 (36.8)	1 (9.1)	37 (15)	47 (17.1)	4 (17.4)	18 (34)	**< 0.0001**
P2Y_12_ inhibitors, *n* (%)	21 (3)	9 (9.5)	0 (0)	4 (1.6)	5 (1.8)	0 (0)	3 (5.7)	**0.002**
Oral anticoagulants, *n* (%)	56 (8)	15 (15.8)	0 (0)	19 (7.7)	12 (4.4)	4 (17.4)	6 (11.3)	**0.004**
Ca channel antagonists, *n* (%)	67 (9.5)	20 (21.1)	0 (0)	13 (5.3)	26 (9.5)	4 (17.4)	4 (7.6)	**0.001**
Statins, *n* (%)	136 (19.4)	39 (41.1)	1 (9.1)	35 (14.2)	35 (12.7)	3 (13)	23 (43.4)	**< 0.0001**
**NYHA class**								
I, *n* (%)	204 (29)	15 (15.8)	3 (27.3)	75 (30.5)	9 (34.9)	4 (17.4)	11 (20.8)	**0.006**
II, *n* (%)	201 (28.6)	26 (27.4)	3 (27.4)	54 (22)	94 (34.2)	12 (52.2)	12 (22.6)	**0.005**
III, *n* (%)	232 (33)	45 (44.2)	3 (27.4)	88 (35.8)	67 (24.4)	7 (30.4)	25 (47.2)	**0.001**
IV, *n* (%)	66 (9.4)	12 (12.6)	2 (18.2)	29 (11.8)	18 (6.7)	0 (0)	5 (9.4)	**0.116**

Data is presented as mean and 95% confidence interval (95% CI). Significant (*p* < 0.05) values are highlighted in bold.

**Table 2:** Laboratory parameters and morphological features of the patient population

	All patients (*n* = 703)	MCM (*n* = 95, 13.5%)	ACM (*n* = 11, 1.6%)	DCM (*n* = 246, 35%)	NDLVC (*n* = 275, 39.1%)	HCM (*n* = 23, 3.3%)	RCM (*n* = 53, 7.5%)	*p*-Value
**Laboratory parameters**								
NT-proBNP (mg/dL) (mean, 95% CI)	9637.1 (5747–13,527)	13446.6 (4671–22,222)	4133.3 (0–36,177)	7567.5 (431–14,674)	7681.6 (1397–13,966)	1416.5 (0–40,662)	18489.5 (5408–31,571)	0.623
Troponin I (ng/ml) (mean, 95% CI)	1.5 (0.7–2.3)	0.4 (0–2.5)	0.1 (0–5.8)	0.3 (0–1.7)	3 (1.8–4.1)	0.2 (0–4.4)	1.3 (0–3.9)	0.065
CK (U/l) (mean, 95% CI)	175.7 (143.1–208.4)	141.4 (56.3–226.4)	93.7 (0–338.3)	139.9 (81.5–198.3)	218.1 (166.8–269.4)	280 (110.9–449.1)	135.8 (22.2–249.4)	0.216
CRP (mg/dl) (mean, 95% CI)	2.1 (1.7–2.4)	1.5 (0.7–2.4)	2.5 (0–5.1)	1.4 (0.8–2)	2.9 (2.4–3.4)	1 (0–2.7)	1.8 (0.7–2.9)	**0.004**
Total-cholesterol (mg/dL) (mean, 95% CI)	182.5 (178.1–186.9)	177.4 (166.3–188.4)	180.5 (142.1–218.9)	183.8 (175.9–191.7)	185.7 (175.6–192.7)	185.6 (164.1–207.2)	170.9 (155.2–186.6)	0.559
HbA1c (%) (mean, 95% CI)	6.5 (6.3–6.7)	6.8 (6.4-7.3)	5.6 (2.9–8.3)	6.6 (6.3–7)	6.2 (5.9–6.5)	5.9 (5–6.8)	6.3 (5.6–6.9)	0.127
Renal function (GFR) (mean, 95% CI)	93.6 (92.8–94.4)	91.3 (89.2-93.3)	102.6 (96.6–108.6)	93.2 (91.9–94.5)	95.7 (94.5–96.9)	98.7 (94.6–102.9)	84.5 (81.8–87.2)	**< 0.0001**
**Echocardiographic parameters**								
LVEF (%) (mean, 95% CI)	42.6 (41.5–43.7)	39.4 (36.9–41.8)	50.5 (43.5–57.6)	32.4 (30.7–34.1)	49 (47.5–50.4)	49.2 (44.2–54.2)	51.4 (48.2–54.7)	**< 0.0001**
LVEDD (mm) (mean, 95% CI)	53.3 (52.5–54.3)	54 (52.3–55.7)	49.7 (44.2–55.2)	62.8 (61.7–63.9)	47.3 (46.3–48.4)	46.3 (43-49.7)	44.3 (42.1–46.5)	**< 0.0001**
PAPsys (mmHg) (mean, 95% CI)	29.1 (28.1–30.2)	31.5 (28.8–34.2)	23.1 (14.4–31.9)	28.5 (26.6–30.3)	28.1 (26.4–29.8)	27.7 (22.4–33)	33.9 (30.3–37.5)	**0.018**
**Cardiac MRI parameters**								
LGE, n (%)	233 (60.7)	26 (57.8)	5 (62.5)	80 (60)	95 (64.3)	9 (64.3)	18 (85.7)	0.231
LVEF (%) (mean, 95% CI)	41.2 (39.6–42.8)	35.9 (32.2–39.7)	59.4 (50.4–68.5)	29.4 (27.1–31.6)	49.1 (47.2–51)	49.7 (43.1–56.3)	44.3 (38.8–49.8)	**< 0.0001**
**Follow-up**	**n** = **624**	**n** = **90**	**n** = **11**	**n** = **199**	**n** = **250**	**n** = **23**	**n** = **51**	
Death, n (%)	139 (22.3)	17 (18.9)	3 (27.3)	47 (23.6)	46 (18.4)	7 (30.4)	19 (37.3)	0.059
Cardiovascular death, n (%)	130 (20.8)	17 (18.9)	3 (27.3)	45 (22.6)	41 (16.4)	6 (29.1)	18 (35.3)	0.058
ICD implantation, n (%)	238 (38.1)	33 (36.3)	7 (63.6)	116 (58.9)	48 (19.2)	15 (65.2)	19 (36.5)	**< 0.0001**
Appropriate ICD therapy, n (%)	57 (9.1)	5 (5.6)	2 (18.2)	25 (12.6)	16 (6.4)	7 (30.4)	2 (3.9)	**< 0.001**

Data is presented as mean and 95% confidence interval (95% CI). Significant (*p* < 0.05) values are highlighted in bold.

**Fig. 2:**
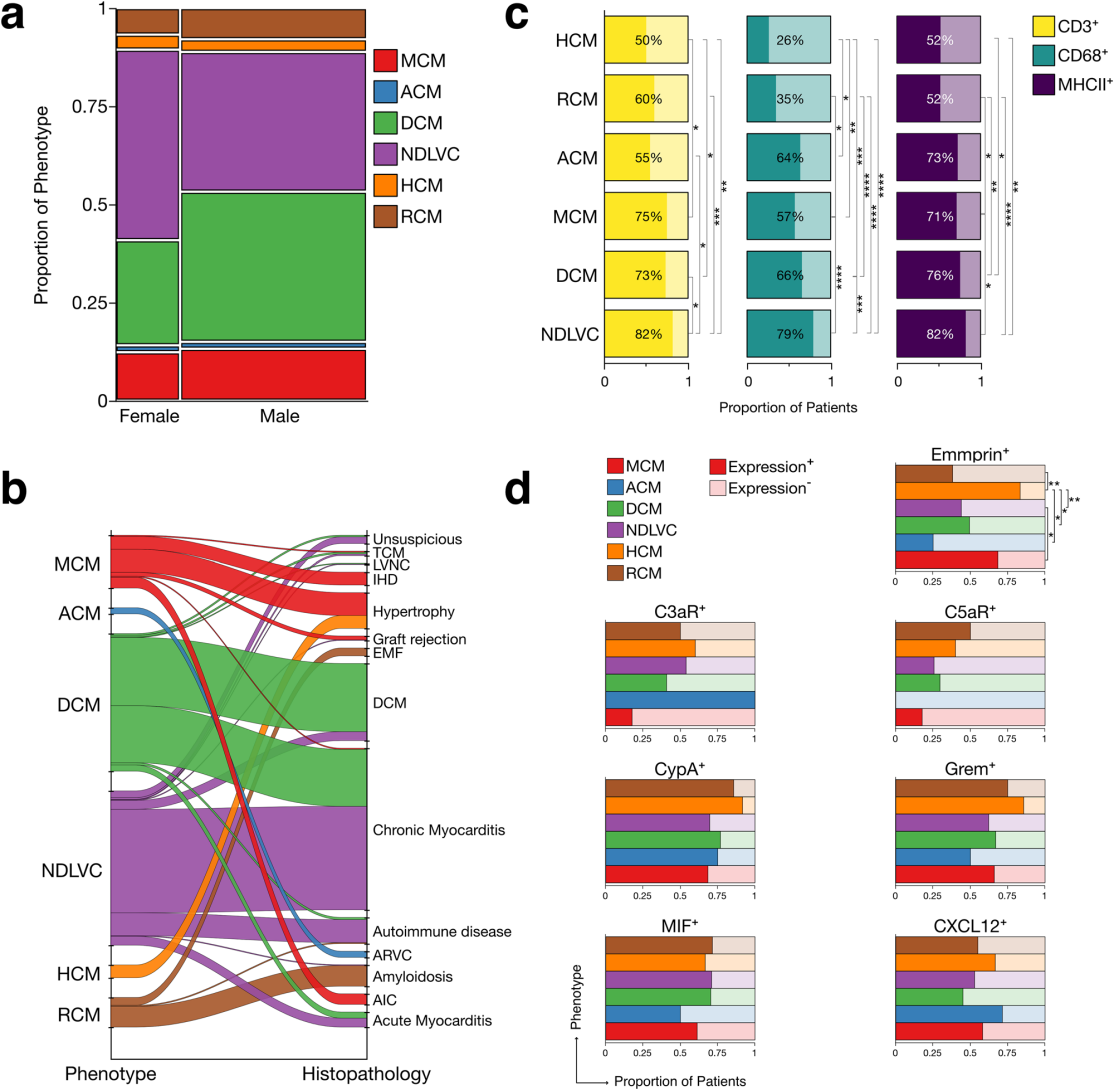
Implementation of endomyocardial biopsy into the phenotypic classification approach to cardiomyopathies. **a** Distribution of phenotypes between female and male patients with cardiomyopathy (*n* = 703). ACM arrhythmogenic cardiomyopathy, DCM dilative cardiomyopathy, HCM hypertrophic cardiomyopathy, MCM mixed phenotype, NDLVC non-dilated left ventricular cardiomyopathy, RCM restrictive cardiomyopathy. **b** Sankey plot illustrating the association of histological diagnoses in relation to the underlying phenotype (*n* = 703). AIC arrhythmia-induce cardiomyopathy, ARVC arrhythmogenic right ventricular cardiomyopathy, DCM dilative cardiomyopathy, EMF endomyocardial fibrosis, IHD ischemic heart disease, LVNC left ventricular non-compaction, TCM toxic cardiomyopathy. **c** Proportion of CD3^+^ T cells (*n* = 632), CD68^+^ macrophages (*n* = 633) and MHCII^+^ cells (*n* = 633) in the myocardium of patients with distinct NICM. Two-sided Pearson’s chi-squared test. **d** Endomyocardial expression of fibroinflammatory mediators (e.g., Emmprin (*n* = 235), C3aR (*n* = 93), C5aR (*n* = 93), cyclophilin A (CypA; *n* = 237), Gremlin-1 (Grem; *n* = 362), macrophage migration inhibitory factor (MIF; *n* = 357), and C-X-C motif chemokine ligand 12 (CXCL12; *n* = 370) in patients with NICM. Two-sided Pearson’s chi-squared test. *p < 0.05, **p < 0.01, ***p < 0.001, ****p < 0.0001.

To establish a comprehensive baseline of phenotypic and histological characteristics in NICM, we first assessed structural, inflammatory, and immunohistochemical patterns across cardiomyopathy subtypes. Non-dilated left ventricular cardiomyopathy (NDLVC; *n* = 275, 39.1%) was most frequent, followed by DCM (*n* = 246, 35%), RCM (*n* = 53, 7.5%), HCM (*n* = 23, 3.3%), and ACM (*n* = 11, 1.6%). A mixed phenotype (MCM) was observed in 13.5% (*n* = 95). Phenotypes differed significantly between female and male patients (*p* = 0.04) (Fig. 2a). Histological classifications mainly revealed inflammation, hypertrophic or fibrotic remodelling, and distinct cardiomyopathy-related pathologies (Fig. 2b). Inflammatory assessments following Dallas^47^ and ESC (Caforio)^48^ criteria varied across phenotypes: CD3^+^ T cells (82%), CD68^+^ macrophages (79%), and MHCII^+^ cells were highly present in NDLVC (82%) versus others (*p* < 0.0001) (Fig. 2c). Patients with NICM had enhanced CD3^+^ T cells (*p* < 0.01), CD68^+^ macrophages (*p* < 0.0001), and MHCII^+^ cells (*p* < 0.0001) compared to controls (Suppl. Fig. S2), indicating a pro-inflammatory myocardial milieu.

Therefore, we additionally assessed inflammatory and pro-fibrotic cytokines, cell surface receptors, and mediators of patients with NICM (Fig. 2d). Here, we found that Emmprin (CD147, basigin) varied significantly among phenotypes (*p* = 0.04) (Fig. 2d), while component receptors C3aR, C5aR, cyclophilin A (CypA), Gremlin-1 (Grem1), macrophage migration inhibitory factor (MIF), and C-X-C motif chemokine 12 (CXCL12) expressions did not. A detailed cardiopathological analysis, including Gremlin-1 expression across phenotypes, is shown in Suppl. Fig. S3. While these analyses demonstrate phenotype-associated inflammatory patterns, they do not explain the mechanisms underlying adverse myocardial remodelling, prompting further investigation of fibroinflammatory drivers.

### Gremlin-1 is associated with cardiac remodelling and supports guideline-based classification of cardiomyopathies

**Fig. 3:**
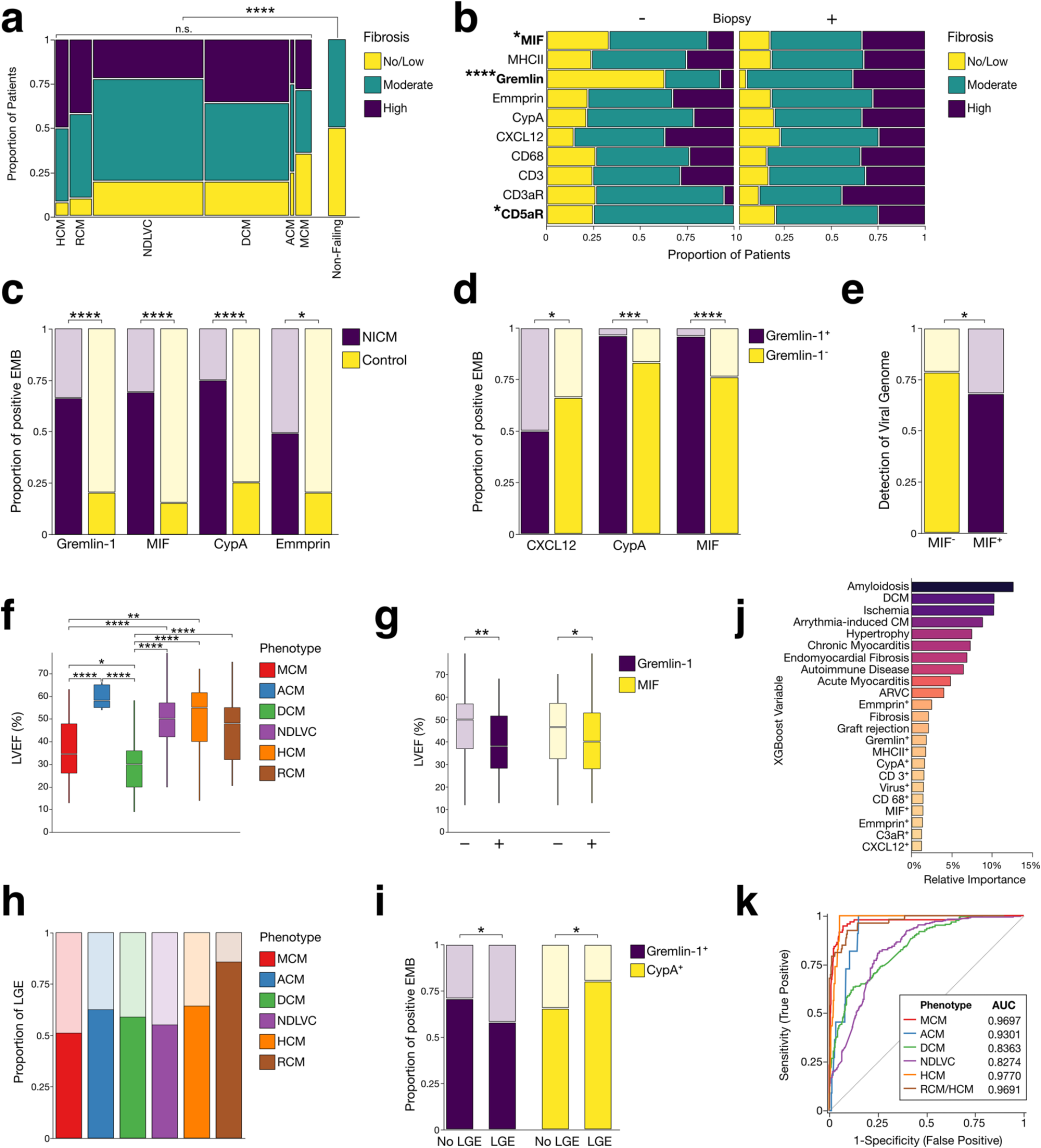
Gremlin-1 is associated with fibroinflammation, adverse cardiac remodelling and increases the diagnostic accuracy in patients with cardiomyopathy. **a** Extend of endomyocardial fibrosis was not associated with the underlying phenotype (*p* = 0.35) but significantly (*p* < 0.0001; two-sided Pearson’s chi-squared test) differed between patients with NICM (*n* = 210) and controls with non-failing hearts (*n* = 20). **b** Degree of cardiac fibrosis in patients with negative (left pane) or positive (right pane) immunohistochemical staining for fibroinflammatory markers (*n* = 362). Patients with Gremlin-1^+^, MIF^+^, and CD5aR^+^ EMB exhibited a critically enhanced extend of endomyocardial fibrosis when compared to those patients with negative staining. Two-sided Pearson’s chi-squared test. **c** Expression of Gremlin-1 (n = 362), MIF (n = 357), CypA (n = 237) and Emmprin (*n* = 235) was significantly enhanced in patients with NICM when compared to non-failing hearts. Two-sided Pearson’s chi-squared test. **d** Interplay between Gremlin-1, the MIF/CXCL12 axis and CypA expression. Immunohistochemical analyses demonstrated enriched abundance of MIF (*n* = 353) and CypA (*n* = 237) in Gremlin-1^+^ tissue sections, whereas expression of CXCL12 (*n* = 281) was decreased in the Gremlin-1^+^ myocardium^.^ Two-sided Pearson’s chi-squared test. **e** Abundance of viral genomes was less frequent in MIF^+^ tissue sections in contrast to MIF^-^ EMB. Two^-^sided Pearson’s chi-squared test (*n* = 115). **f** Comparison of left ventricular functional capacity from cardiac MRI images unveiled significantly divergent LVEF between distinct phenotypes (*p* < 0.0001). Two-sided Mann Whitney U test (*n* = 345). **g** LVEF was critically reduced in patients with Gremlin-1^+^ and MIF^+^ EMB compared to patients with negative staining. Two-sided Mann Whitney U test (*n* = 200). **h** Distribution of late gadolinium enhancement (LGE) was not varying between cardiomyopathy phenotypes (*p* = 0.16). Two-sided Pearson’s chi-squared test (*n* = 373). **i** In patients with LGE on cardiac MRI, EMB unveiled a significantly enriched expression of CypA (*n* = 144), whereas abundance of Gremlin-1 (*n* = 232) was decreased compared to patients without LGE. Two-sided Pearson’s chi-squared test. **j** Most important histological parameters predicting the morphological phenotype in patients with NICM. (*n* = 633) **k** Receiver operating characteristic (ROC) analysis of XGBoost model unveiled a high accuracy to predict the distinct phenotype as outlined by areas under the curve (AUC). *p < 0.05, **p < 0.01, ***p < 0.001, ****p < 0.0001.

To identify key fibroinflammatory drivers of myocardial remodeling beyond phenotypic classification, we next examined the association between histological markers, particularly Gremlin-1, and structural and functional cardiac alterations. Although the extent of endomyocardial fibrosis did not differ significantly across phenotypes (*p* = 0.35), fibrosis was markedly increased in patients with non-ischemic cardiomyopathy compared with non-failing controls (*p* < 0.0001) (Fig. 3a). Fibrosis correlated with the myocardial expression of fibroinflammatory mediators. Above all, fibrosis was highly enriched in Gremlin-1^+^ (*p* < 0.0001), MIF^+^ (*p* = 0.02), and CD5aR^+^ (*p* = 0.04) biopsies compared to negative samples (Fig. 3b). Multivariable analysis identified Gremlin-1 as an independent predictor of cardiac fibrosis (Suppl. Table S2). NICM tissues showed significantly elevated Gremlin-1 (66% vs. 20%, *p* < 0.0001), MIF (68.9% vs. 15%, *p* < 0.0001), CypA (74.6% vs. 25%, *p* < 0.0001), and Emmprin (48.9% vs. 20%, *p* = 0.01) expression compared to non-failing hearts (Fig. 3c), suggesting a crucial interplay of these markers in disease progression. High Gremlin-1 expression was associated with increased MIF (96.1% vs. 76.2%, *p* < 0.0001) and CypA (96.4% vs. 83.3%, *p* < 0.001) levels (Fig. 3d). Conversely, CXCL12 expression was significantly decreased in Gremlin- 1^+^ samples (50.35% vs. 66.3%, *p* = 0.01) (Fig. 3d). Viral genomes (adenoviruses, enteroviruses, Epstein-Barr virus, HHV-6, parvovirus B19) were detected in 65% of NICM samples (*p* = 0.02) (Suppl. Fig. S4). Viral presence was associated with reduced myocardial MIF expression (65% vs. 82.9%, *p* < 0.05) (Fig. 3e).

To evaluate whether Gremlin-1–associated fibroinflammatory remodelling is reflected in structural and functional myocardial impairment, we assessed cardiac imaging parameters. Consistent with adverse remodelling, LVEF significantly differed across NICM phenotypes as assessed by MRI (*p* < 0.0001) (Fig. 3f) and echocardiography (*p* < 0.0001) (Suppl. Fig. S5). LVEDD also varied significantly between phenotypes (*p* < 0.0001) (Suppl. Fig. S6). Gremlin-1^+^ patients showed significantly reduced LVEF compared to Gremlin-1^−^ patients (43% vs. 50% in TTE, *p* < 0.01; 38% vs. 50% in MRI, *p* < 0.01) and increased LVEDD (54 mm vs. 51 mm, p = 0.03) (Fig. 3g, Suppl. Figs. S7–S8). The presence of late gadolinium enhancement (LGE) did not vary significantly among phenotypes (*p* = 0.16) (Fig. 3h). However, patients with LGE had higher CypA expression (80.2% vs. 65.5%, *p* < 0.05) and reduced Gremlin-1^+^ expression (58.2% vs. 70.9%, *p* < 0.05) compared to those without LGE (Fig. 3i). Gremlin-1 expression was independent of LGE pattern or myocardial edema based on T1/T2 mapping (Suppl. Fig. S9). LVEF and LVEDD were unaffected by the expression of Emmprin, C3aR, C5aR, CypA, MIF, and CXCL12, highlighting the unique significance of Gremlin-1 as a potential diagnostic marker in NICM. To determine whether EMB-derived histological features can support guideline-based phenotypic classification, we applied machine learning approaches integrating histopathological data. Thus, we performed XGBoost machine learning analysis integrating exclusively histopathological EMB data. Key predictive factors included histomorphology and Gremlin-1 expression (Fig. 3j). The model demonstrated high sensitivity and specificity in distinguishing cardiomyopathy phenotypes (Fig. 3k, Suppl. Fig. S10), although classification between DCM and NDLVC remained limited as these phenotypes are defined by left ventricular diameters assessed through cardiac imaging. Model interpretability was enhanced using SHAP (Shapley additive explanation) values to identify key histological features driving phenotype classification (Suppl. Fig. S11).

### Assessment of mRNA expression signatures in non-ischemic cardiomyopathies

**Fig. 4:**
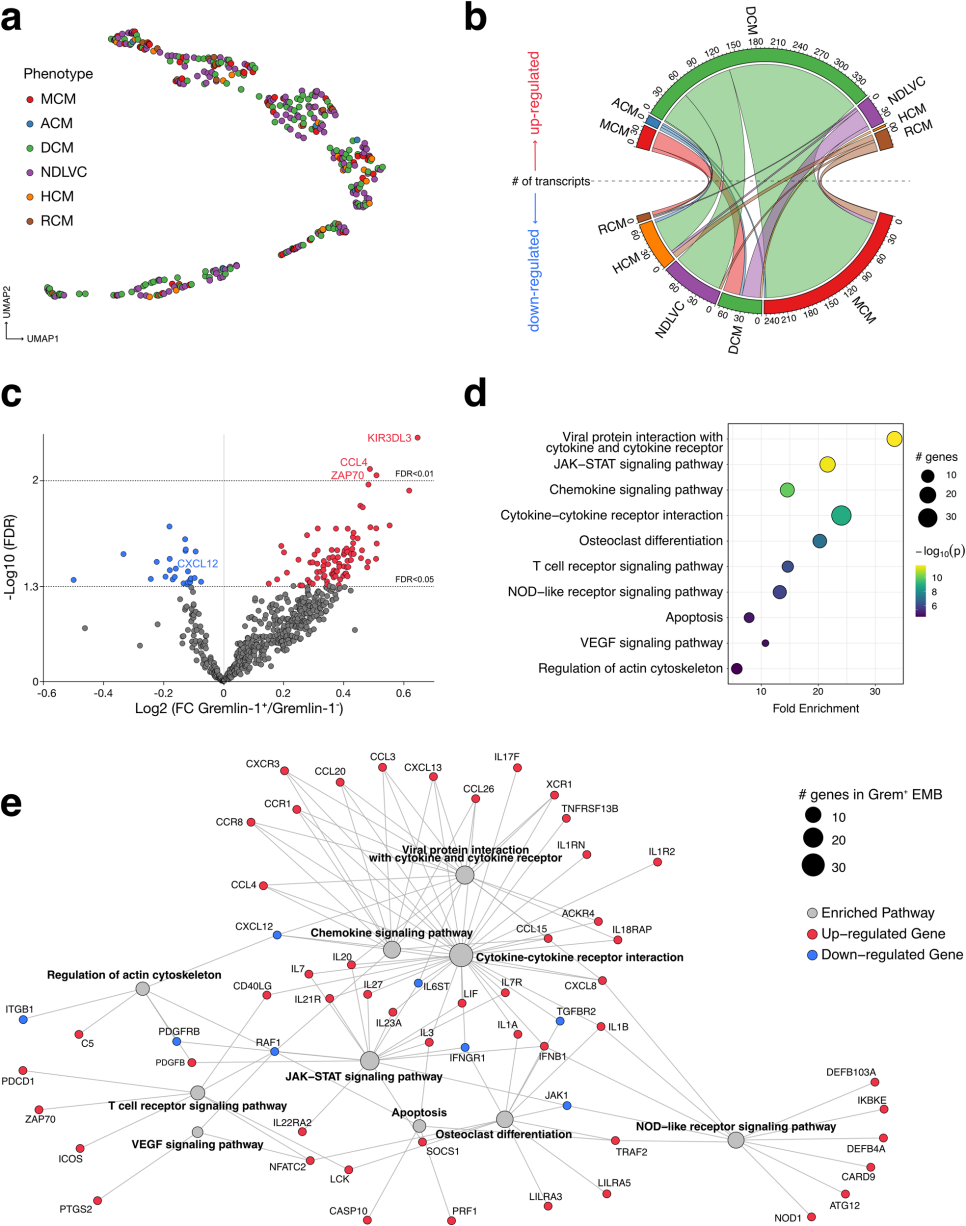
Assessment of mRNA signatures in non-ischemic cardiomyopathies uncovers pathophysiological signalling cascades associated with cardiac remodelling. **a** Uniform Manifold Approximation and Projection (UMAP) of mRNA data from myocardial biopsy samples unveiled a minor separation between cardiomyopathy phenotypes (*n* = 373). **b** Chord chart presents a synopsis of differentially expressed transcripts between distinct phenotypes of NICM (*n* = 373). **c** Results of mRNA data were Log2-transformed, and fold change of transcript was plotted in volcano plot displaying significant alterations between patients with Gremlin-1^+^ and Gremlin-1^−^ EMB (ANOVA *p* < 0.05, BH FDR < 0.05, 89 upregulated, 24 downregulated transcripts in patients with Gremlin-1^+^ EMB; *n* = 281). **d** Kyoto Encyclopaedia of Genes and Genomes (KEGG) pathway analysis was performed. The top ten significantly enriched pathways primarily govern inflammation, cellular death, and cascades critical for structural integrity of the myocardium. ANOVA, BH FDR < 0.05; *n* = 281. **e** Term-gene-graph of subnetworks and enriched KEGG pathways with the differentially expressed genes in patients with Gremlin-1^+^ EMB (*n* = 281).

To further elucidate the molecular pathways underlying Gremlin-1–associated remodelling, we performed targeted myocardial mRNA expression profiling in patients with non-ischemic cardiomyopathy, focusing on transcripts involved in inflammatory and fibrotic remodelling processes (Suppl. data 1). Uniform Manifold Approximation and Projection (UMAP) of mRNA data from paraffin-embedded myocardial biopsy samples unveiled a substantial overlap across patients, with no clear segregation according to NICM phenotype (Fig. 4a). This finding indicates that global mRNA expression patterns are largely shared among NICM entities and that the influence of phenotype on transcriptomic profiles is modest rather than discriminatory. However, in-depth juxtaposition of distinct phenotypes indicated specific multiplex mRNA expression within the subgroups of NICM (Fig. 4b & Suppl. data 2). Next, we aimed to uncover pathophysiological cascades contributing to adverse cardiac remodelling. Therefore, we sought to identify differentially expressed mRNAs between patients with Gremlin-1^+^ and Gremlin-1^−^ EMB. We found that, among 594 tested transcripts, 89 were significantly up-regulated (*p* < 0.05, FDR < 0.05), whereas 24 mRNA transcripts were down-regulated in Gremlin-1^+^ tissue sections compared to Gremlin-1^−^ samples (*p* < 0.05, FDR < 0.05) (Fig. 4c).

We identified predominantly differentially expressed transcripts enriched in inflammation, cellular death, and pro-fibrotic myocardial remodelling, based on the Kyoto Encyclopedia of Genes and Genomes (KEGG) database (Fig. 4d, e). In particular, T cell and cytokine-chemokine mediated inflammation, JAK-STAT, NOD-like, and VEGF signalling pathway, apoptosis, osteoclastogenesis and calcification, as well as regulation of cardiomyocyte actin cytoskeleton were significantly (p < 0.01) enriched in patients with Gremlin-1^+^ EMB (Fig. 4d, e).

### Association of adverse cardiovascular events with endomyocardial expression of Gremlin-1

**Fig. 5:**
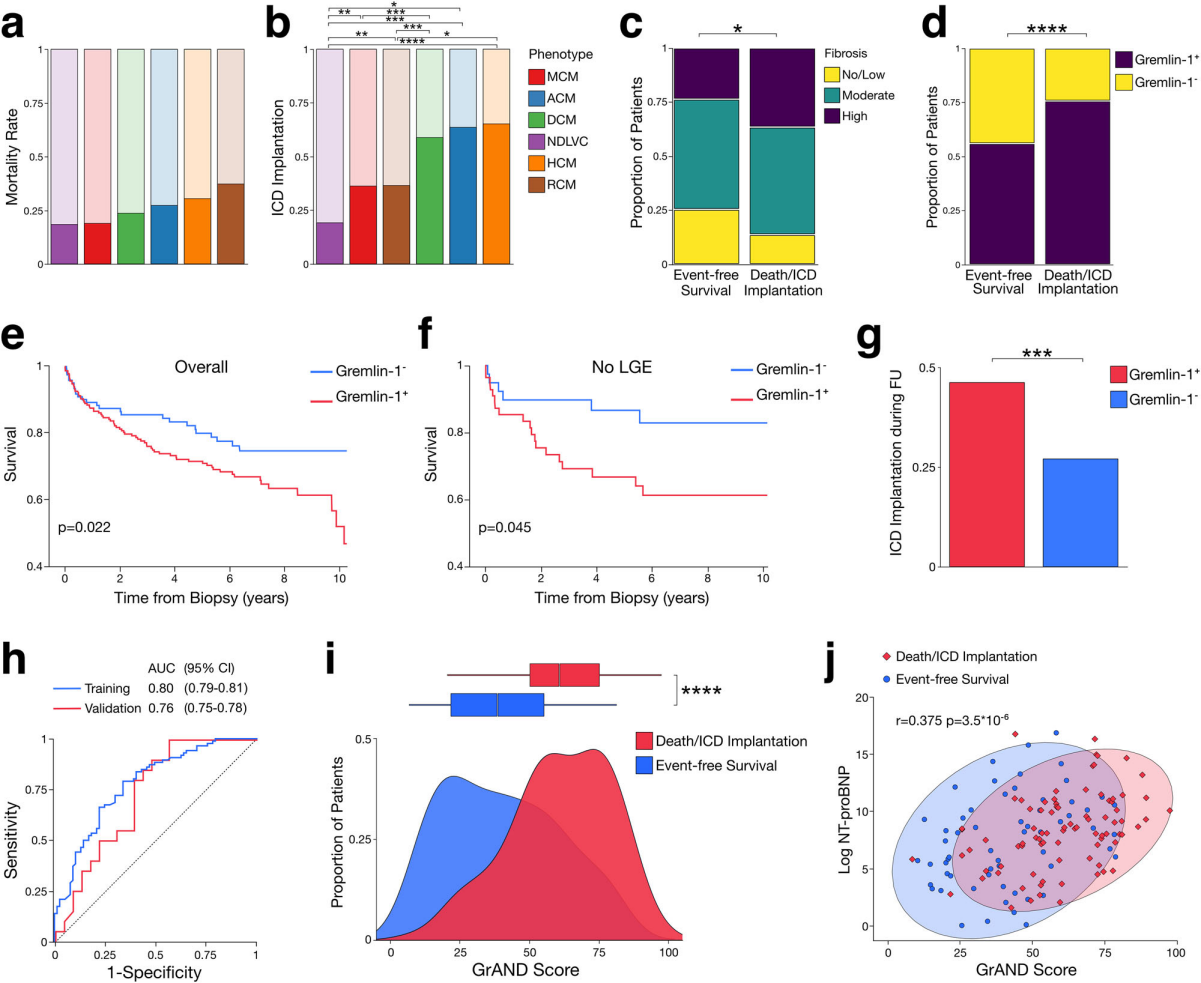
Expression of Gremlin-1 is critically associated with adverse cardiovascular events and predicts clinical outcome in patients with cardiomyopathy. **a** The mortality rate during the clinical follow-up in this study showed a trend among cardiomyopathy phenotypes but did not reach significance (*p* = 0.06; two-sided Pearson’s chi-squared test; *n* = 624). **b** Likelihood of ICD implantation during the follow-up period was highly dependent on the underlying phenotype (*p* < 0.0001; two-sided Pearson’s chi-squared test; *n* = 624). **c** Comparison of endomyocardial fibrosis depicts enhanced fibrotic remodelling in patients with adverse events compared to those without adverse events. Two-sided Pearson’s chi-squared test; *n* = 210. **d** Cardiac expression of Gremlin-1 was critically enriched in non-survivors and patients undergoing ICD implantation compared to patients without adverse events. Two-sided Pearson’s chi-squared test; *n* = 360. **e** Kaplan-Meier curves showing transformed 10-year estimates of all-cause mortality in patients with cardiomyopathy (i = 360). Failure curves exhibit a significantly (*p* < 0.05) increased risk of patients with Gremlin-1^+^ EMB. **f** Kaplan–Meier transformed 10-year estimates of all-cause mortality in patients without LGE in cardiac MRI affirmed an increased risk of patients with Gremlin-1^+^ EMB (*p* < 0.05) (*n* = 377)^.^ **g** Eligibility of ICD implantation during the follow-up (FU) period was significantly enhanced in patients with Gremlin-1^+^ EMB when compared to patients with Gremlin-1^−^ EMB. Two-sided Pearson’s chi-squared test; *n* = 360. **h** Receiver operating characteristic (ROC) analysis of cross-validated LASSO model predicting all-cause mortality and ICD implantation in patients with NICM (*n* = 703). Area under the curves (AUC) of the machine learning model integrating Gremlin-1 expression unveiled a major prediction accuracy to estimate the cardiovascular risk for future events. The model was trained (blue curve; *n* = 527, 75%) in a random set of patients and cross-validated in the test cohort (red curve; *n* = 176, 25%). **i** Transformed likelihood ratio from LASSO model (GrAND score) showed a high sensitivity to distinguish between patients with adverse events (red) and those without (blue) adverse events during the clinical follow-up. Two-sided Mann Whitney U test. **j** GrAND score was significantly associated with concentrations of NT-proBNP and thus might indicate disease activity in patients with NICM (Spearman´s ρ; *n* = 112). *p < 0.05, **p < 0.01, ***p < 0.001, ****p < 0.0001.

To assess the clinical relevance of Gremlin-1–associated remodeling, we investigated its association with long-term cardiovascular outcomes. During the ten-year clinical follow-up period of 703 patients (2462.7 person-years), 139 individuals died (19.8%), and 238 individuals (33.9%) underwent ICD implantation, corresponding to a cumulative incidence rate of 153.1 (95% CI 138-169.3) per 1000 person-years. Of note, all-cause mortality was not significantly associated with the underlying cardiomyopathy (overall p = 0.06), but there was a trend towards predominant phenotypes (non-survivors per phenotype: RCM 37.3%, HCM 30.4%, ACM 27.3%, DCM 23.6%, MCM 18.9%, NDLVC 18.4%) (Fig. 5a). On the contrary, ICD implantation was most frequent in patients with HCM, ACM, and DCM, revealing a clear association with the phenotype (ICD implantation per phenotype: HCM 65.2%, ACM 63.6%, DCM 58.9%, RCM 36.5%, MCM 36.3%, NDLVC 19.2%; overall *p* < 0.0001) (Fig. 5b). In line with previous findings indicating an increased risk of developing adverse cardiovascular events through cardiac remodelling, we observed a significantly (*p* = 0.04) increased extent of myocardial fibrosis among non-survivors (Fig. 5c). Most strikingly, the expression of Gremlin-1 was significantly enriched (p < 0.0001) in patients who died or underwent early ICD implantation during follow-up (75.7% Gremlin-1^+^) when compared to patients without adverse events (56.2% Gremlin-1^+^) (Fig. 5d). Gremlin-1 was associated with all-cause mortality (*p* = 0.02) based on Kaplan-Meier transformed survival analyses. Thus, patients with Gremlin-1^+^ EMB had a significantly (*p* = 0.022) increased 10-year overall mortality rate (48.1%) compared to patients with Gremlin-1^−^ EMB (25.4%) (Fig. 5e). Furthermore, this observation was equally prominent in patients presenting without late gadolinium enhancement (10-year mortality rate of 38.3 % in patients with Gremlin-1^+^ EMB versus 17.6% in patients with Gremlin-1^−^ EMB; *p* < 0.05) (Fig. 5f). Notably, the occurrence of LGE was not associated with the endpoint of all-cause or cardiovascular mortality in survival analyses (Suppl. Fig. S12) which underscores the importance of Gremlin-1. Subsequently, cardiovascular mortality was significantly (*p* = 0.047) increased in patients with Gremlin-1^+^ EMB (Suppl. Fig. S13).

Moreover, ICD implantation was highly more frequent in patients with Gremlin-1^+^ EMB compared to those with Gremlin-1^−^ EMB (46.2 versus 27.1%, p < 0.001) (Fig. 5g). In addition, appropriate ICD discharge was significantly (*p* = 0.027) more frequent in patients with Gremlin-1^+^ EMB during the 10-year follow-up (Suppl. Fig. S14). Multivariable analysis identified Gremlin-1 as independent predictor of the combined endpoint including all-cause mortality and ICD implantation (Suppl. Table S5). Likewise, in the multivariable analysis Gremlin-1 was an independent predictor for the combined endpoint including all-cause mortality and appropriate ICD discharge Suppl. Table S6. Notably, apart from Gremlin-1, immunohistological parameters were not associated with all-cause mortality or appropriate ICD discharge in survival analysis (Suppl. Figs. S15 & S16).

Finally, to translate these findings into a clinically applicable framework, we developed and validated a multivariable risk prediction model incorporating EMB-derived features. Variable selection was performed using a cross-validated LASSO penalized regression approach adjusted for age, sex, and cardiomyopathy phenotype (Suppl. Fig. S17). In the L_1_-regularized model predicting mortality or ICD implantation, Gremlin-1 exhibited a non-zero coefficient and thus was related to disease severity in patients with NICM (Suppl. Table S7 & S8). The cross-validated model exhibited a high predictive value to identify patients at risk (Fig. 5h & Suppl. Table S8). The area under the curve of receiver operating characteristic (ROC) analyses was 0.80 (95% CI 0.79–0.81) in the training cohort and 0.76 (95% CI 0.75–0.78) in the validation cohort. Sensitivity to predict disease progression in patients with NICM was 0.79 (95% CI 0.78–0.80) in the training cohort and 0.81 (95% CI 0.79–0.82) in the validation cohort. The negative predictive value was 0.69 (95% CI 0.67–0.70) and 0.67 (95% CI 0.64–0.69), respectively. Thus, the **Gremlin-1** in **A**cute **N**on-ischemic Heart **D**isease (GrAND) score demonstrated a high level of significance (*p* < 0.0001) in distinguishing patients at risk of disease progression from those without adverse events (Fig. 5i). Strikingly, GrAND score was associated with concentrations of NT-proBNP. Thus, high scores of the model integrating histological data such as Gremlin-1 to predict adverse events correlated with the reliable biomarker natriuretic peptide (Fig. 5j). Together, these multilevel analyses demonstrate that EMB-derived histological features capture disease-defining biology in NICM and provide complementary diagnostic and prognostic value beyond conventional classification. Therefore, histological investigation of cardiac Gremlin-1 might become a powerful tool to estimate disease activity in patients with NICM.

### Extended follow-up and translational liquid biopsy validation of Gremlin-1

**Fig. 6:**
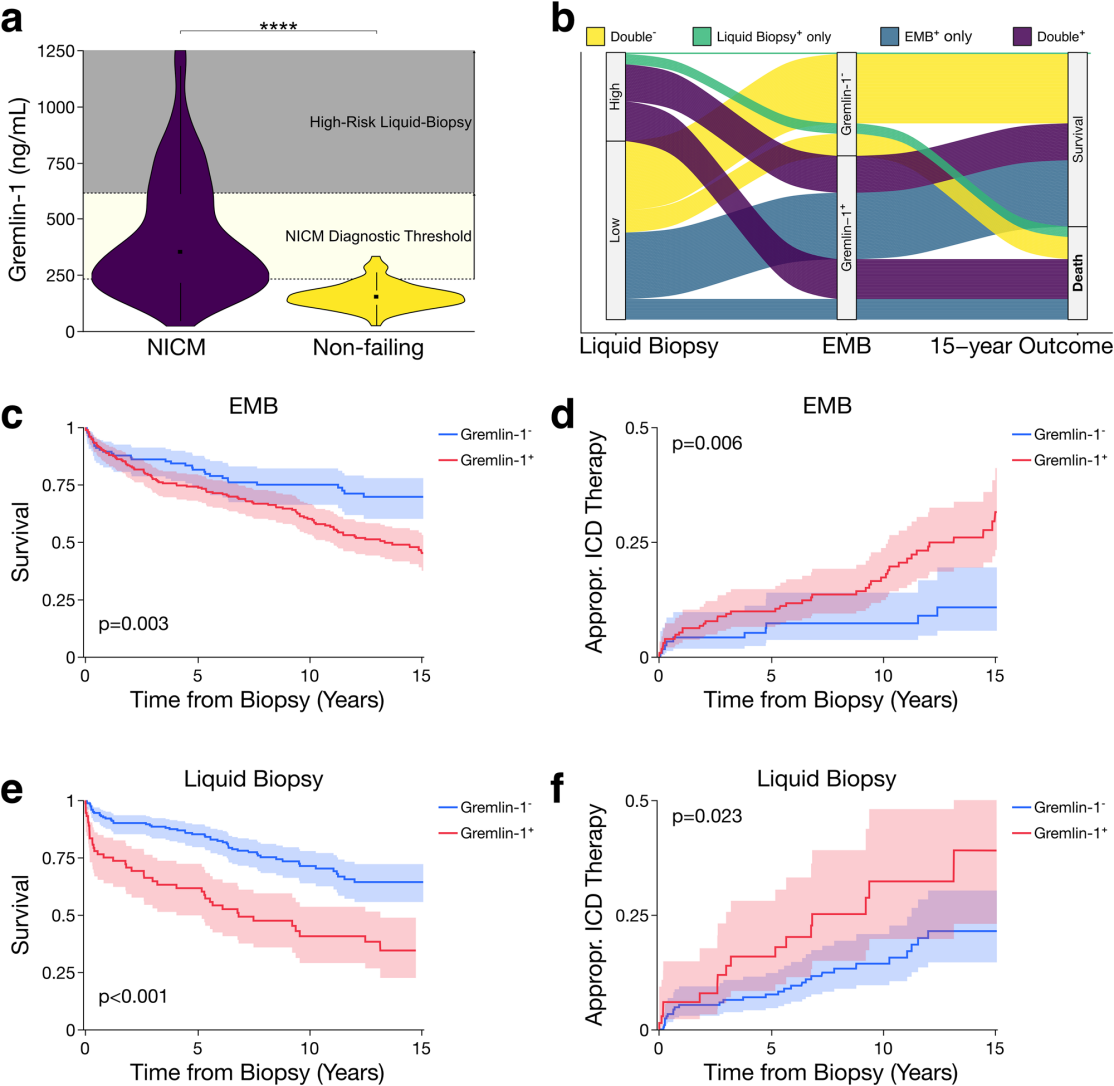
Gremlin-1 bridges tissue pathology and liquid biopsy to improve long-term risk stratification in non-ischemic cardiomyopathy. **a** Circulating plasma Gremlin-1 concentrations in patients with non-ischemic cardiomyopathy (NICM; *n* = 301) and non-failing controls (*n* = 75). Plasma Gremlin-1 was significantly (two-sided Mann-Whitney U test) elevated in NICM patients. Receiver operating characteristic (ROC)-derived thresholds identified a NICM diagnostic threshold of 235 ng/l for discrimination of NICM from healthy controls and a separate high-risk liquid biopsy threshold of 614 ng/l for long-term prognostic stratification. ****p < 0.0001. **b** Combined liquid biopsy and endomyocardial biopsy (EMB) Gremlin-1 status revealed stepwise risk stratification of 15-year mortality. Patients with both elevated plasma Gremlin-1 and Gremlin-1–positive EMB (double positive) showed the highest mortality, whereas double-negative patients demonstrated the most favorable long-term outcome. **c** Kaplan–Meier analysis of 15-year all-cause mortality according to myocardial Gremlin-1 expression in EMB. Patients with Gremlin-1^+^ EMB showed significantly increased long-term mortality compared with Gremlin-1^-^ patients. **d** Kaplan-Meier analysis of appropriate ICD therapy according to myocardial Gremlin-1 expression in EMB. Gremlin-1^+^ EMB was associated with a significantly increased risk of ventricular arrhythmic events requiring ICD therapy. **e** Kaplan–M**e**ier analysis of 15-year all-cause mortality according to circulating plasma Gremlin-1 levels (liquid biopsy). Patients above the predefined high-risk prognostic threshold demonstrated significantly impaired long-term survival. **f** Kaplan–Meier analysis of appropriate ICD therapy according to circulating plasma Gremlin-1 levels. Elevated plasma Gremlin-1 identified patients at significantly increased risk for ventricular arrhythmias and appropriate ICD discharge.

To further assess the long-term prognostic relevance of Gremlin-1 and address its potential clinical applicability beyond invasive EMB, we performed extended follow-up analyses up to 15 years (mean 7.4 years) and evaluated circulating plasma Gremlin-1 as a liquid biopsy marker. Plasma Gremlin-1 concentrations were significantly elevated in patients with NICM compared with healthy controls, supporting its diagnostic value as a circulating biomarker (Fig. 6a). Combined analysis of EMB tissue and liquid biopsy revealed stepwise risk stratification, with patients positive for both EMB and plasma Gremlin-1 (double positive) showing the highest long-term mortality, whereas double-negative patients demonstrated the most favorable prognosis (Fig. 6b). These findings identify Gremlin-1 as a translational biomarker linking myocardial fibroinflammatory remodeling with a less invasive circulating surrogate for persistent cardiovascular risk assessment. Patients with Gremlin-1^+^ EMB continued to exhibit significantly increased all-cause mortality (*p* = 0.003) (Fig. 6c) and a higher incidence of appropriate implantable cardioverter-defibrillator (ICD) therapy (*p* = 0.006) during 15-year follow-up compared with Gremlin-1^−^ patients (Fig. 6d). Similarly, elevated circulating plasma Gremlin-1 above the predefined high-risk liquid biopsy threshold was associated with significantly impaired long-term survival (*p* < 0.001) (Fig. 6e) and increased risk of appropriate ICD discharge (*p* = 0.023) (Fig. 6f).

## Discussion

The major results of the present study are: (i) Patients with non-ischemic cardiomyopathy experience significant structural changes obtained from endomyocardial biopsy (ii) Gremlin-1, a mediator of fibrosis and inflammation, was associated with adverse cardiac remodelling (iii) Among the identified histological changes, the expression of Gremlin-1 correlated with disease severity and was found to exhibit substantial accuracy in indicating poor prognosis of patients with NICM. Our findings imply that, in line with current guidelines, integration of EMB and additional histological and plasmatic assessment of Gremlin-1 may enhance the diagnostic accuracy of cardiomyopathies. Hitherto, accurate classification of NICM has remained challenging, and conventional cardiovascular risk factors often fail to precisely estimate the individual risk of developing adverse events in patients with NICM.

At present, prognostic risk stratification in NICM relies largely on clinical and imaging-based parameters, and no validated risk score integrating histological endomyocardial biopsy data is available^49–51^. Previously, we and others demonstrated that implementation of EMB in the diagnostics of NICM patients critically increases the diagnostic value, and histological assessment of cardiac remodelling is a prognostic indicator related to poor outcome of patients with cardiovascular disease^8–10,35,36^. In this study, we systematically analysed structural cardiac abnormalities in NICM patients using the current phenotypic classification.

Distinct phenotypes showed characteristic histomorphological changes and specific inflammatory patterns, confirming the key role of inflammation in dilated and non-dilated cardiomyopathy, often linked to myocarditis^7,52^. Enhanced myocardial expression of inflammatory cell populations in patients with NICM compared to non-failing hearts suggests a key role of inflammation contributing to cardiac remodelling and progressive HF. The extent of cardiac fibrosis in non-ischemic cardiomyopathy was independent of the underlying phenotype but was significantly increased compared with non-failing hearts and closely associated with a fibroinflammatory myocardial signature. Although several circulating and tissue-based fibrosis markers, including galectin-3, ST2, and collagen turnover markers, have been proposed, their capacity to capture active myocardial remodelling at the tissue level remains limited, underscoring the potential advantage of powerful EMB-derived biomarkers^53–55^. In this context, Gremlin-1, a potent downstream profibrotic mediator of the TGF-β pathway, showed a strong and consistent association with myocardial fibrosis^56,57^. Interestingly, Gremlin-1 was previously stated to govern both adverse cardiac remodelling and cardioprotective limitation of myocardial inflammation and fibrosis^8,58,59^. Gremlin-1 has been recognized as an antagonist of bone morphogenetic proteins (BMPs)^57^. Only recently, in a mouse model of myocarditis, the BMP/Gremlin-1 axis was shown to maintain homeostatic heart function, and inhibition of Gremlin-1 ameliorated T cell-induced myocardial inflammation^60^. Thus, Gremlin-1 may become a druggable target to halt the progression of NICM and terminal HF. Beyond histological changes in Gremlin-1^+^ EMB patients, we observed altered myocardial gene expression affecting inflammation, cell death, and cardiomyocyte function. In line with previous EMB-based studies in NICM, the frequent detection of cardiotropic viral genomes in our cohort likely reflects viral persistence rather than active replication, supporting the concept that latent viral presence may contribute to or modulate the chronic myocardial inflammatory milieu rather than represent overt viral disease^61,62^. Aberrant mRNA signatures in NICM patients may reflect key inflammatory, fibrotic, and remodelling pathways. Accordingly, transcriptomic analyses focused on identifying shared and Gremlin-1–associated regulatory signalling rather than on molecular classification of NICM phenotypes, given biological overlap and the focal nature of EMB sampling. Consistently, Gremlin-1^+^ EMB was associated with reduced left ventricular function. While there is sufficient evidence of the negative impact of reduced LVEF on cardiovascular outcome in patients with cardiomyopathies, risk stratification and treatment regimens including device-based therapy are not solely based on left ventricular function^3,63^. Therefore, multimodal diagnostic approaches in patients with NICM often include cardiac MRI and assessment of LGE patterns^64,65^. Strikingly, Gremlin-1 was found be critically enriched in NICM patients without evidence of LGE, which otherwise might have resulted in underestimation of the cardiovascular risk. This observation supports the concept that structural imaging markers reflect cumulative myocardial injury^66^, whereas histological markers such as Gremlin-1 may capture ongoing disease activity, thereby identifying patients at risk before irreversible remodelling becomes apparent. Subsequently, in this study, presence of LGE failed to solely distinguish patients at risk. Similarly, in survival analysis, neither EMB patterns, including fibrosis, nor conventional markers were linked to poor prognosis, except for Gremlin-1. Thus, in this cohort, Gremlin-1 emerges as an independent predictor of adverse events in patients with NICM at a subclinical level, where conventional parameters such as LGE or subendocardial fibrosis fail to effectively stratify the individual risk. Of note, reduced histological expression and plasmatic concentration of Gremlin-1 in non-failing hearts suggest a potential role as a promising prognostic biomarker of NICM and progressive HF. In the present study, Gremlin-1 was a significant predictor of mortality, ICD discharge, and guideline-based ICD implantation in patients with NICM. Confirming past findings^8^, Gremlin-1 exhibited a high degree of sensitivity for prediction of mortality in patients with HF and was demonstrated to be independent of NICM phenotype, or additional risk measures such as LGE. Therefore, assessment of Gremlin-1 expression in EBM may be a useful tool to identify patients at risk for disease progression. Remarkably, we found that by machine learning of histological parameters, EMB allows to correctly identify the underlying NICM phenotype and thus might strengthen the guideline-based classification approach.

Similarly, the histological GrAND score that incorporates Gremlin-1 to predict future events showed a significant correlation with NT-proBNP and thus might indicate disease activity in patients with NICM. In this context, Gremlin-1–guided phenotyping aligns with emerging precision cardiology approaches that aim to tailor diagnostic and therapeutic strategies based on underlying disease biology rather than phenotypic appearance alone^67^. Therefore, implementation of EMB and staining of Gremlin-1 may become a valuable strategy to tailorize treatment regimens including implantation of cardiac devices. At present, the results of our study and our preliminary experimental approach promote a hypothesis that a differential expression of cardiac Gremlin-1 has consequences for inflammation and pro-fibrotic cardiac remodelling associated with an increased cardiovascular risk. Although therapeutic targeting of Gremlin-1 remains speculative, the present findings provide a clinical rationale for further experimental and translational studies exploring modulation of fibroinflammatory signalling in NICM.

The significance of our study is limited by the diagnostic algorithm used, requiring patient reclassification according to updated guidelines at study entry. We present innovative real-world data from an all-comers HF cohort, including HFrEF, HFmrEF, and HFpEF, potentially influencing the generalizability of our findings. We acknowledge that we do not provide direct evidence linking structural EMB changes to individual risk for adverse events. A further limitation of this study is the incomplete availability of multimodal data across all patients. In addition, genetic testing was not performed in all patients, which may limit the interpretation of genotype–phenotype relationships. Not all individuals had complete datasets for histological, immunohistochemical, molecular, and imaging analyses. Missing data primarily resulted from variability in tissue quality and quantity for EMB-based assessments, as well as the clinical applicability of specific diagnostic procedures. Consequently, analyses were performed using available-case data without imputation, which may introduce bias and limit generalizability. Importantly, this reflects the real-world clinical workflow of EMB diagnostics, where core histological assessments are performed routinely, while extended molecular analyses depend on sufficient tissue availability. Accordingly, the observed associations should be interpreted in the context of available data and do not exclude the possibility of residual confounding or selection effects. Nevertheless, the consistency of findings across independent analytical modalities supports the robustness of the observed associations, although causal inference cannot be inferred. Immunohistochemical data were assessed semiquantitative following established protocols, and fibrosis was assessed as overall burden rather than architectural subtypes, which may partially reflect both the focal nature of fibrosis and, to some extent, inherent sampling limitations of EMB. Using machine learning, we demonstrate that integration of histological data routinely available in EMB diagnostics enables accurate prediction of the underlying NICM phenotype with high diagnostic precision. Although EMB is inherently invasive and not routinely performed in all patients with suspected NICM, tissue-level assessment remains highly valuable for identifying disease-relevant myocardial remodeling. Importantly, the present extended 15-year follow-up and exploratory liquid biopsy analyses support the concept that Gremlin-1 may serve not only as a tissue-based prognostic marker but also as a mechanistic anchor for the development of less invasive circulating biomarkers. While external validation is currently lacking, this work provides a strong foundation for future studies, and validation in independent cohorts using harmonized data acquisition protocols will further enhance generalizability and enable robust phenotypic classification and patient stratification based on EMB-derived patterns. Although the control group was smaller compared to the NICM cohort, this reflects ethical and practical constraints in obtaining myocardial tissue from truly healthy individuals, and the use of carefully selected post-mortem samples provides a well-defined and clinically appropriate reference despite the imbalance in group sizes. The concordance between tissue expression and circulating plasma levels supports its potential role as a translational biomarker linking invasive EMB assessment with less invasive liquid biopsy strategies. Future prospective studies should validate Gremlin-1–guided risk stratification and its role in precision cardiology approaches for personalized management of NICM. Ultimately, this study underscores the importance of EMB in the guideline-based diagnostic algorithm for cardiomyopathies. Future studies should evaluate whether EMB-guided risk stratification can improve clinical decision-making efficiency and resource allocation, particularly in patients with borderline indications for device therapy.

## Supplementary information

Transparent Peer Review file

Supplemental Material

Description of Additional Supplementary files

Supplementary Data 1

Supplementary Data 2

Supplementary Data 3

Supplementary Data 4

Supplementary Data 5

Supplementary Data 6

Supplementary Data 7

## Data Availability

Source data underlying the main figures are provided as Suppl. Data Files 3–7: source data for Fig. 2 are included in Suppl. Data 3; source data for Fig. 3 in Suppl. Data 4; source data for Fig. 4 in Suppl. Data 5; source data for Fig. 5 in Supplementary Data 6; source data for Fig. 6 in Supplementary Data 7. The trained XGBoost model has been made publicly available via a persistent repository (10.5281/zenodo.18145288). Access to analysed GRAND cohort data and related study materials will be considered by the University of Tübingen to ensure compliance with institutional intellectual property and confidentiality policies. Anonymized GRAND cohort data will be made available by the corresponding author to qualified academic investigators for the sole purpose of reproducing the analyses and results reported in this manuscript. Data and materials approved for sharing will be released under a material transfer agreement.
