# Supplementary material for: Endomyocardial Gremlin-1 is associated with structural remodeling and adverse clinical outcomes in non-ischemic cardiomyopathy
Source: Commun Med (Lond). 2026 Jul 2;6:373. doi: 10.1038/s43856-026-01762-9 (PMC13328730; doi:10.1038/s43856-026-01762-9)
Supplement: Supplementary file 2 — Supplemental Material [file 43856_2026_1762_MOESM2_ESM.pdf]

Endomyocardial Gremlin-1 Is Associated with Structural Remodeling and Adverse Clinical Outcomes in Non-Ischemic Cardiomyopathy

Short title: Gremlin-1 in **A**cute **N**on-ischemic Heart **D**isease (GrAND)

Tobias Harm[1], Karin Klingel[2], Patrick Krumm[3], David Heinzmann[1], Livia Baas[1], Ioannis Toskas[1], Peter Seizer[4], Jörg Kumbrink[5,6], Thomas Kirchner[5,6], Oliver Borst[1], Jürgen Schreieck[1], Tobias Geisler[1], Simon Greulich[1], Meinrad Paul Gawaz[1], Karin Anne Lydia Müller[1,*]

[1]Department of Cardiology and Angiology, University Hospital Tübingen, Eberhard Karls University Tübingen, Otfried-Müller-Straße 10, 72076 Tübingen, Germany

[2]Cardiopathology, Institute for Pathology and Neuropathology, University Hospital Tübingen, Liebermeisterstrasse 8, 72076, Tübingen, Germany

[3]Department of Diagnostic and Interventional Radiology, University Hospital Tübingen, Hoppe-Seyler-Straße 3, 72076 Tübingen, Germany

[4]Department of Cardiology, Ostalb Clinic Aalen, Im Kaelblesrain 1, 73430 Aalen, Germany

[5]Institute of Pathology, University of Munich, Munich, Germany

[6]German Cancer Consortium, German Cancer Research Center, Heidelberg, Germany

***Correspondence to:**

Karin Anne Lydia Mueller, MD

Department of Cardiology and Angiology

University Hospital Tübingen, Eberhard Karls University Tübingen

Otfried-Müller-Str. 10, 72076 Tübingen, Germany

Tel.: +49 (0) 7071 29 83688, Fax: +49 (0) 7071 29 5749

E-Mail: k.mueller@med.uni-tuebingen.de

Supplementary Figures and Tables

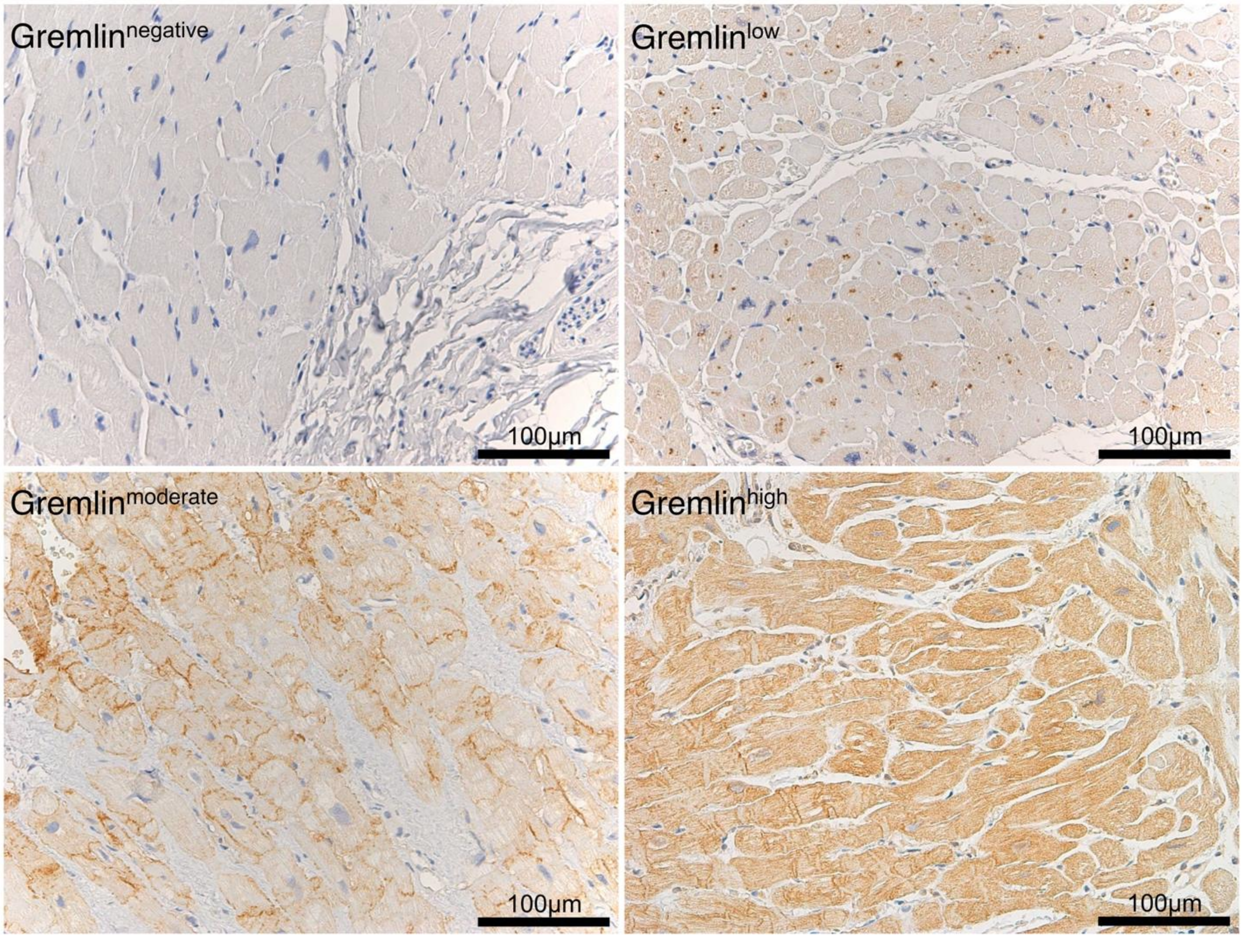

Supplementary Figure S1. Histopathological analysis of endomyocardial Gremlin-1. Representative images of Gremlin-1 expression in EMB of patients with NICM. Tissue sections were analyzed by two primary examiners in a blinded way using a score-based approach, and results were cross-referenced by a trained cardiovascular pathologist. The semiquantitative expression ranged from no/low expression to ubiquitous high expression. Patients were then dichotomized according to expression levels into no/low expression (Gremlin-1^{-}) and moderate/high expression (Gremlin-1^{+}). Scale bar = 100µm.

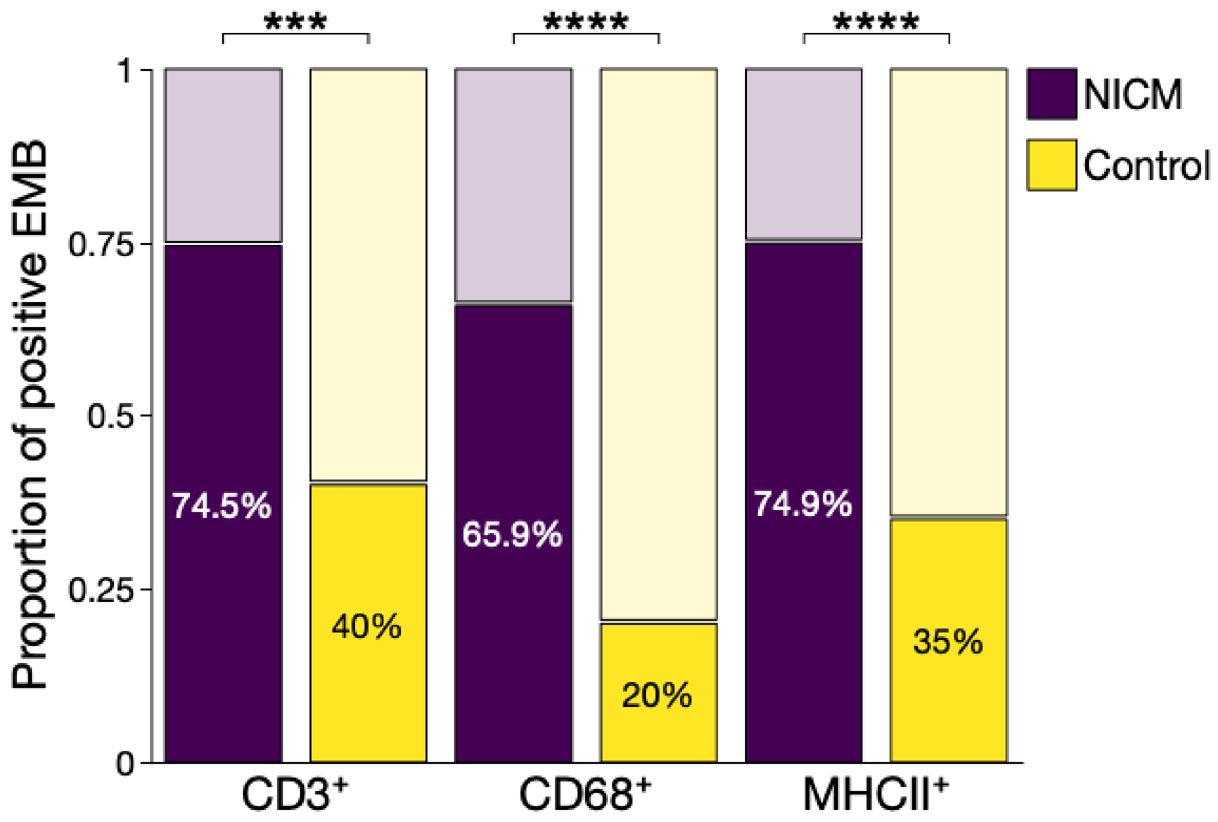

Supplementary Figure S2. Endomyocardial inflammation is enhanced in NICM. $CD3^+$ T cells, $CD68^+$ macrophages, and $MHCII^+$ cells play a key role in myocardial inflammation and were significantly elevated in patients with non-ischemic cardiomyopathy (NICM; n=632 for $CD3^+$ T cells, n=633 for $CD68^+$ macrophages, and $MHCII^+$) compared to controls with non-failing hearts (n=20). ***p<0.001, ****p<0.0001.

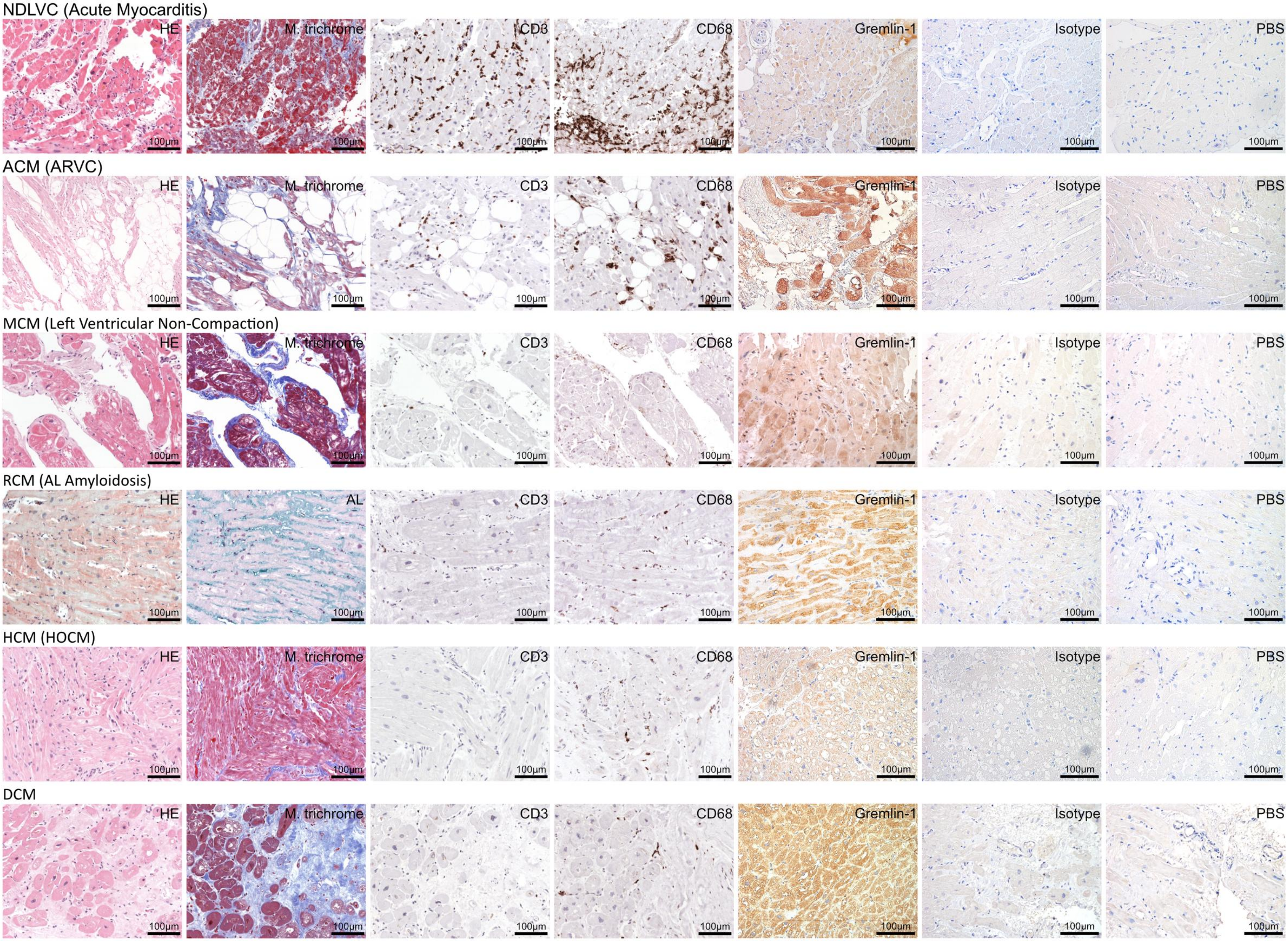

NDLVC (Acute Myocarditis)
ACM (ARVC)
MCM (Left Ventricular Non-Compaction)
RCM (AL Amyloidosis)
HCM (HOCM)
DCM
HE
M. trichrome
AL
CD3
CD68
Gremlin-1
Isotype
PBS
100µm

Supplementary Figure S3. Histological characterisation of non-ischemic cardiomyopathies. Comparison of NICM phenotypes with representative images of EMB tissue sections in hematoxylin and eosin stain (HE) and Masson trichrome (M. trichrome) or anti-amyloid light-chain (AL) staining, as well as immunohistochemical analysis of $CD3^+$ T cells and $CD68^+$ macrophages. Further, we highlight the immunohistological identification of Gremlin-1^+ myocardium with isotype/PBC control staining in patients with NICM. Scale bar = 100μm.

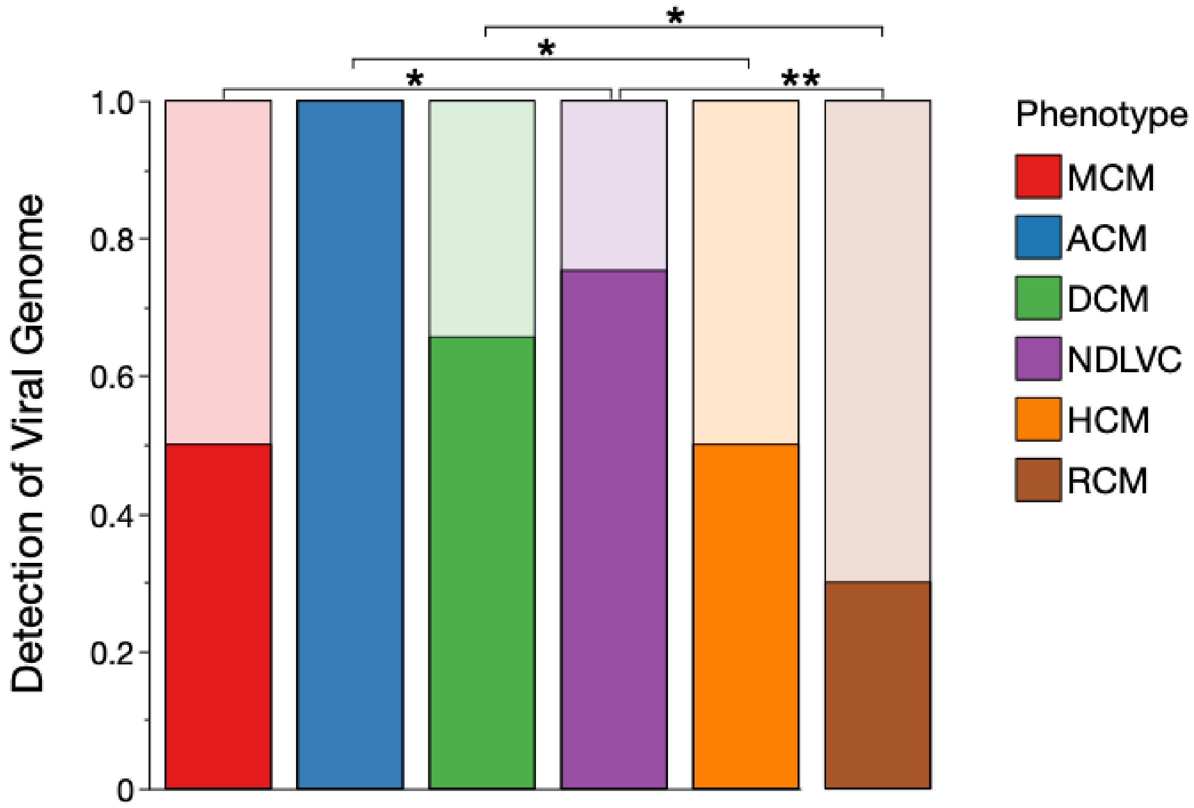

Supplementary Figure S4. Association of myocardial viral genome detection with cardiomyopathy phenotype. Detection of viral genomes within the myocardium was significantly (p=0.02) associated with the cardiomyopathy phenotype (n=185). * p<0.05, **p<0.01.

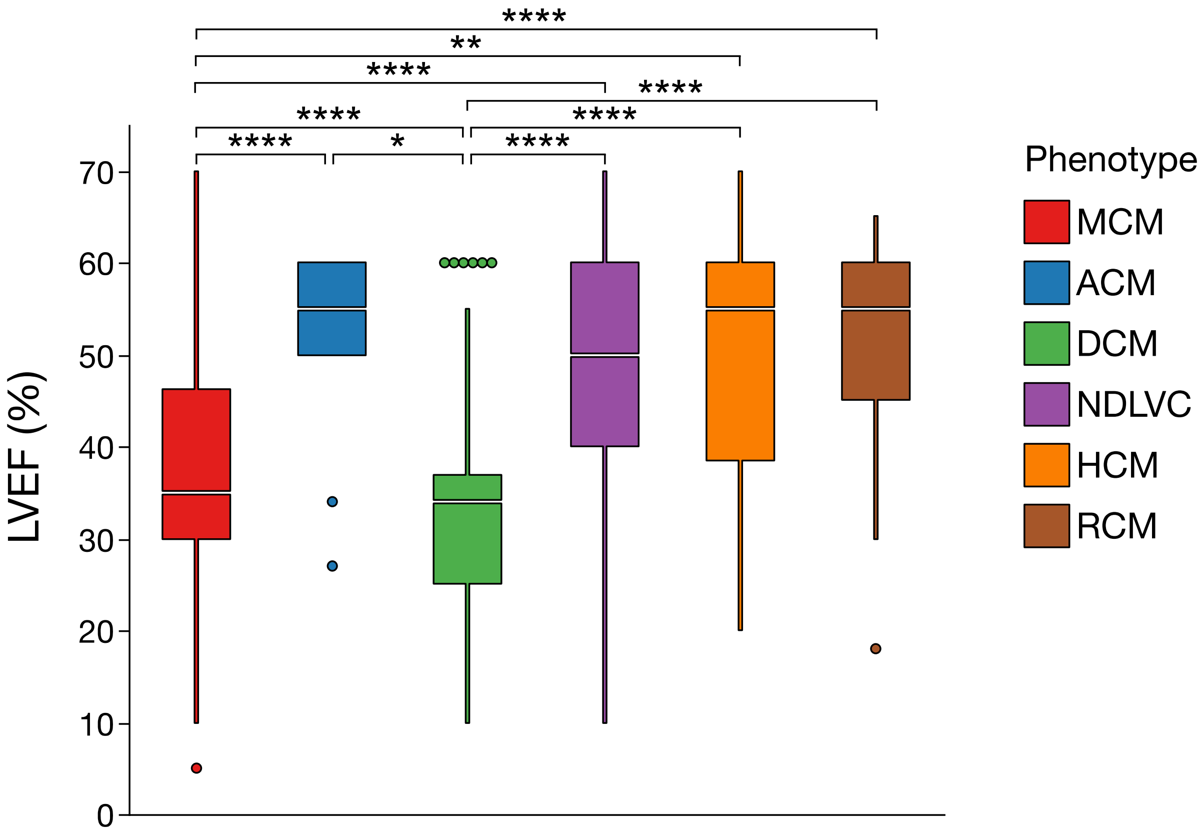

Supplementary Figure S5. Left ventricular ejection fraction (LVEF) differs among cardiomyopathy phenotypes assessed by transthoracic echocardiography. Comparison of LVEF between cardiomyopathy phenotypes (n=618) assessed by transthoracic echocardiography. * p<0.05, **p<0.01, ***p<0.001, ****p<0.0001.

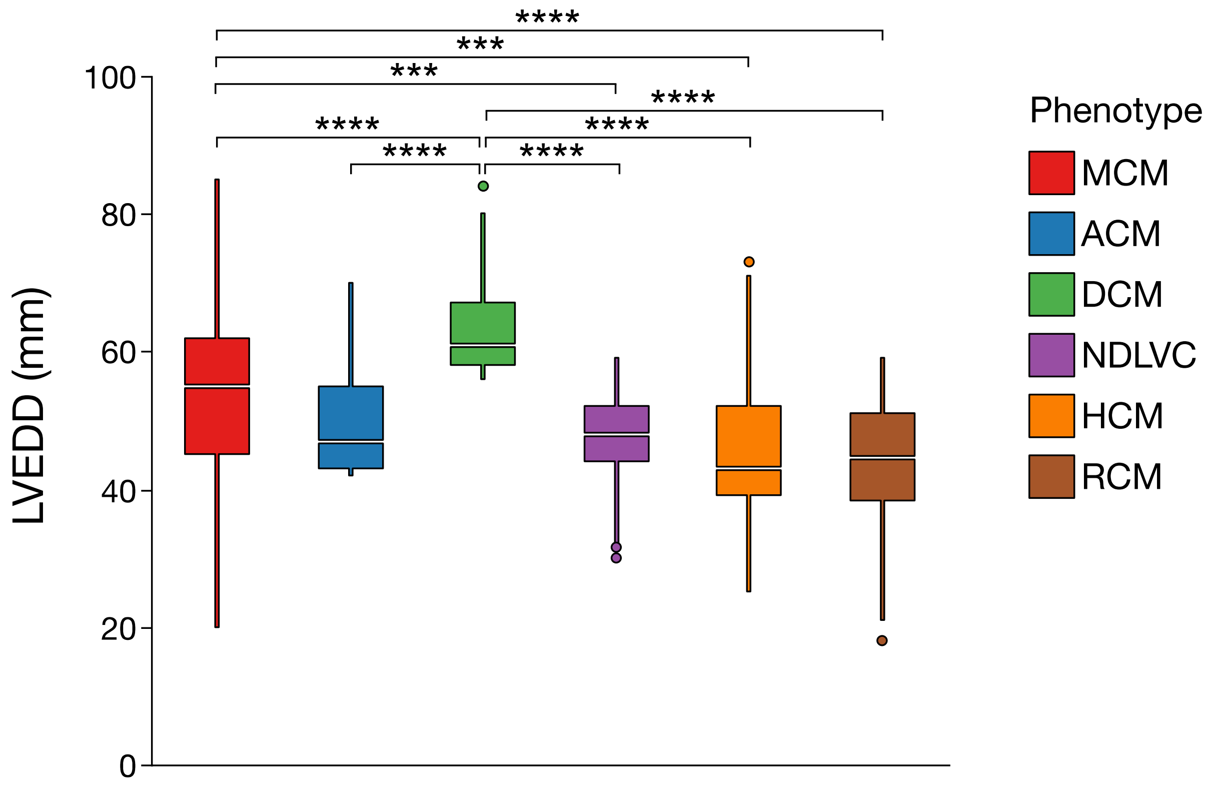

Supplementary Figure S6. Left ventricular end-diastolic diameter (LVEDD) differs among non-ischemic cardiomyopathy phenotypes assessed by transthoracic echocardiography. Comparison of left LVEDD between NICM cardiomyopathy phenotypes (n=527) assessed by transthoracic echocardiography. * $p<0.05$, **$p<0.01$, ***$p<0.001$, ****$p<0.0001$.

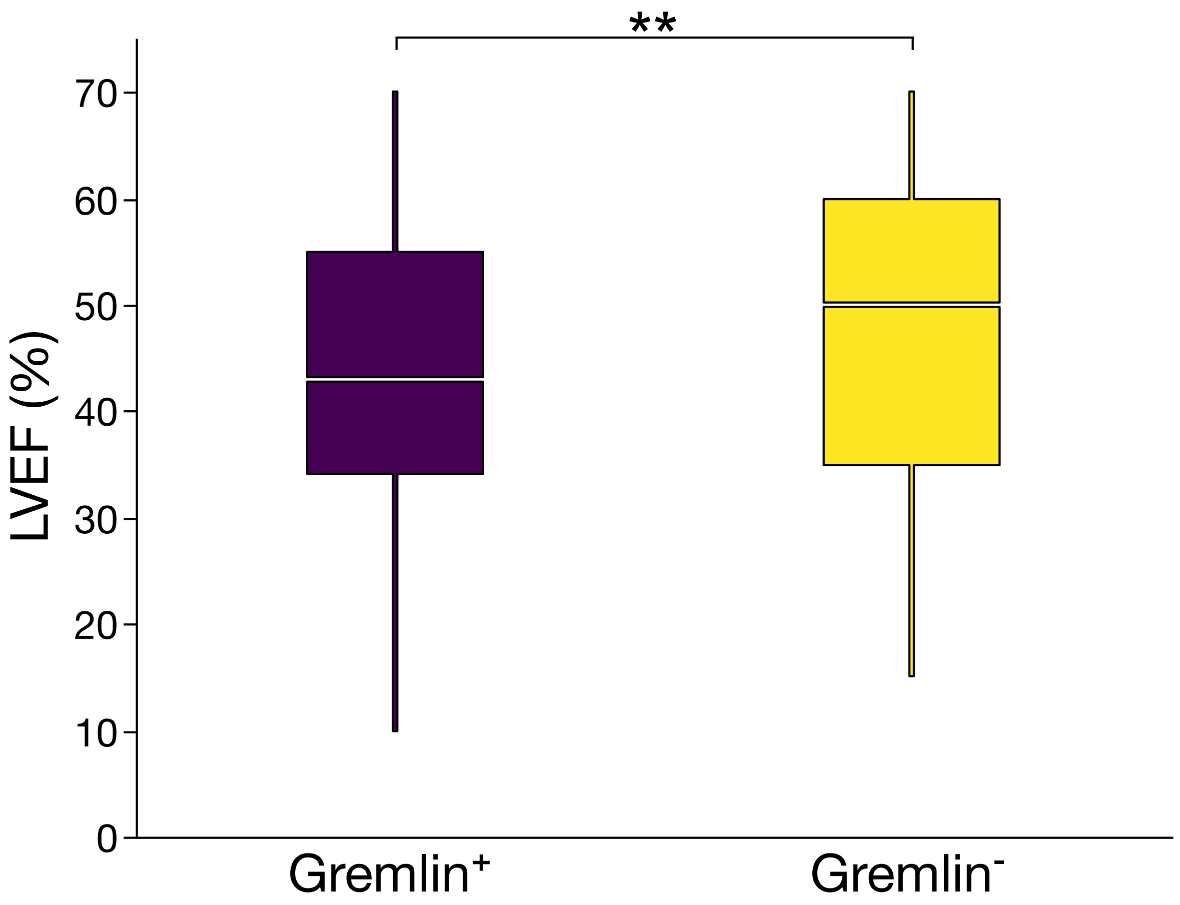

Supplementary Figure S7. Gremlin-1 is associated with reduced left ventricular function. Comparison of left ventricular ejection fraction through transthoracic echocardiography exhibited a significantly reduced left ventricular function capacity in patients with Gremlin$^+$ EMB compared to those with Gremlin$^-$ EMB (n=357). **p<0.01

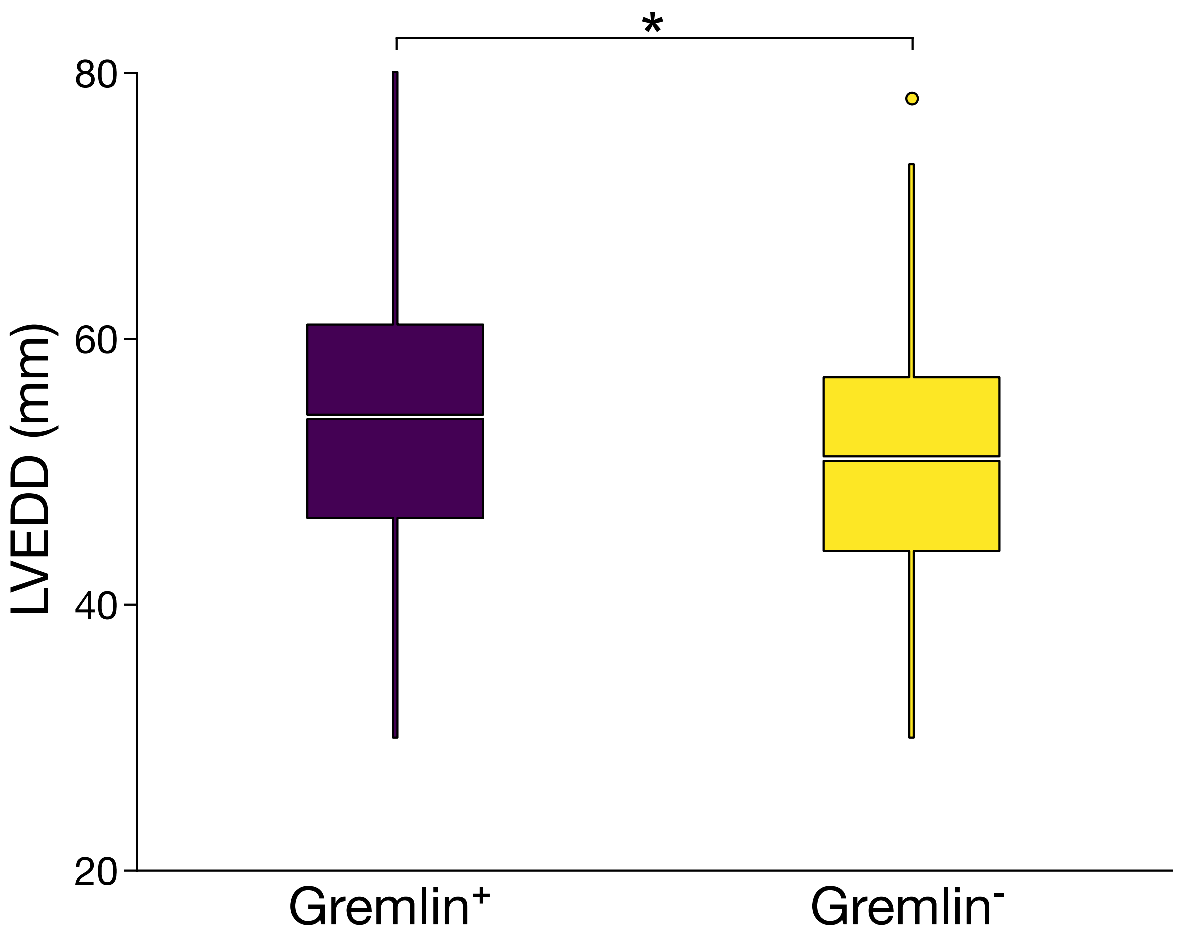

Supplementary Figure S8. Gremlin-1 is associated with adverse cardiac remodeling characterized by left ventricular dilatation. Left ventricular end-diastolic diameter (LVEDD) in transthoracic echocardiography imaging was significantly increased in patients with Gremlin$^+$ EMB when compared to those patients with Gremlin$^-$ EMB (n=341). *$p<0.05$

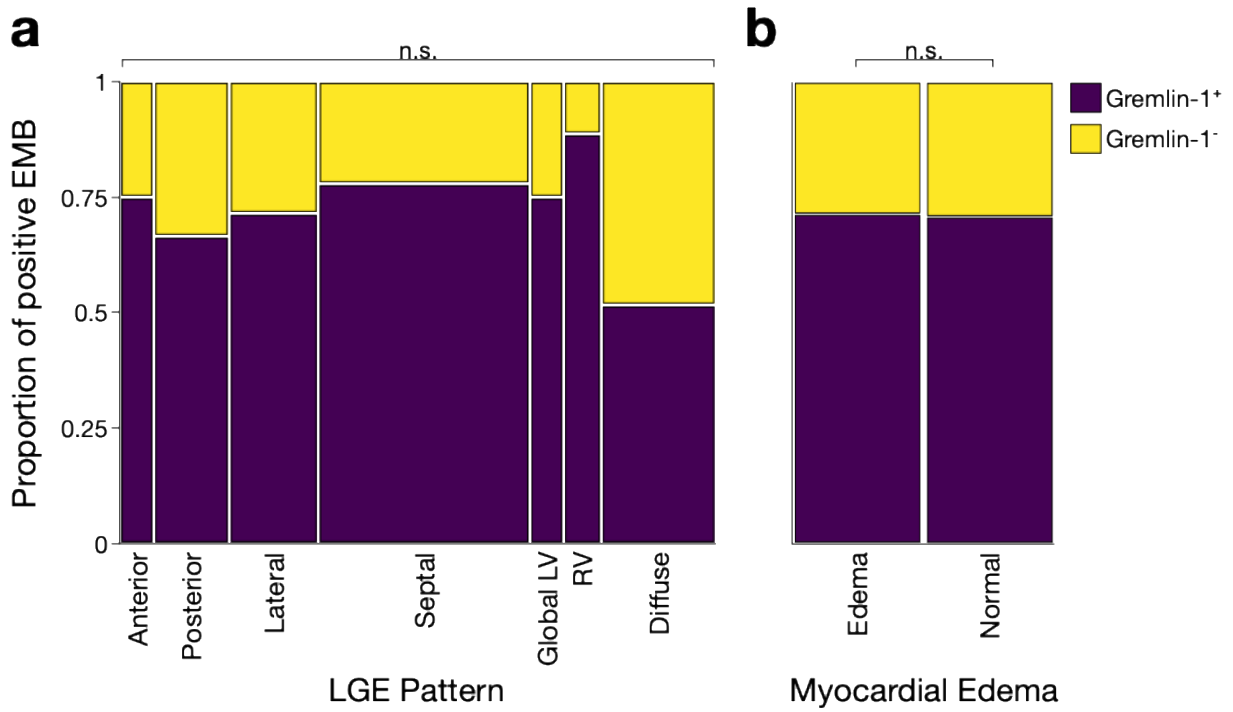

Supplementary Figure S9. Distribution pattern of LGE and myocardial edema among patients with NICM. (A) The expression of Gremlin-1 in endomyocardial biopsy (EMB) did not differ based on the corresponding pattern of late gadolinium enhancement (LGE) observed in cardiac MRI. However, there was a trend toward higher Gremlin-1 expression in patients with right ventricular (RV) LGE compared to those with isolated left ventricular (LV) LGE (n=384). **(B)** The expression of Gremlin-1 did not differ between patients with extracellular edema and those with normal T1/T2 mapping on cardiac MRI (n=377).

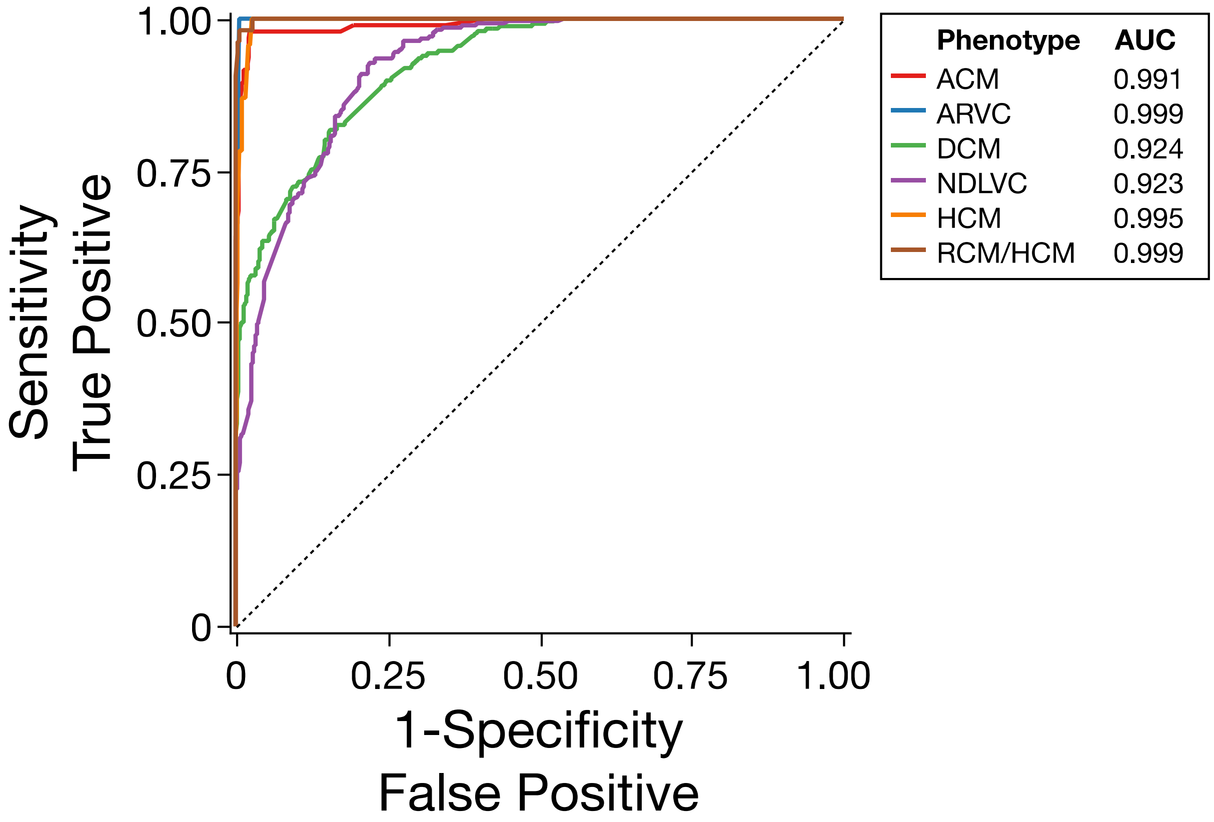

Supplementary Figure S10. XGBoost machine learning model predicting NICM phenotype integrating exclusively histological data. Receiver operating characteristic (ROC) analysis of XGBoost model integrating only histological data to predict the correlating phenotype in the training group of patients with cardiomyopathy. Area under the curve (AUC) unveils a high diagnostic accuracy of the model.

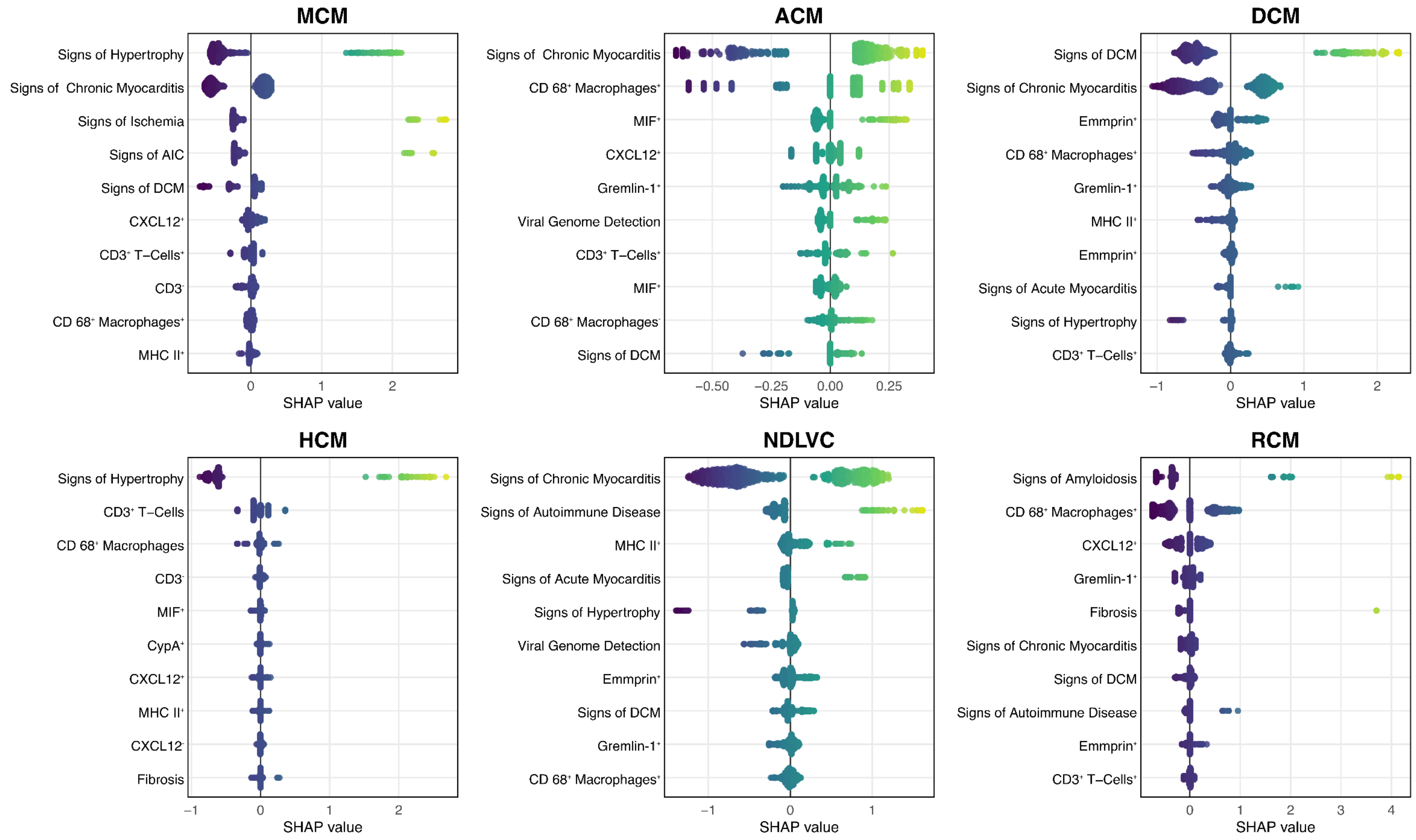

Supplementary Figure S11. Feature Importance of histological prediction of NICM phenotype. SHAP (Shapley additive explanation) beeswarm plots illustrating the top contributing features for each phenotype. The distribution of SHAP values indicates the direction and magnitude of each feature's contribution to phenotype-specific model predictions.

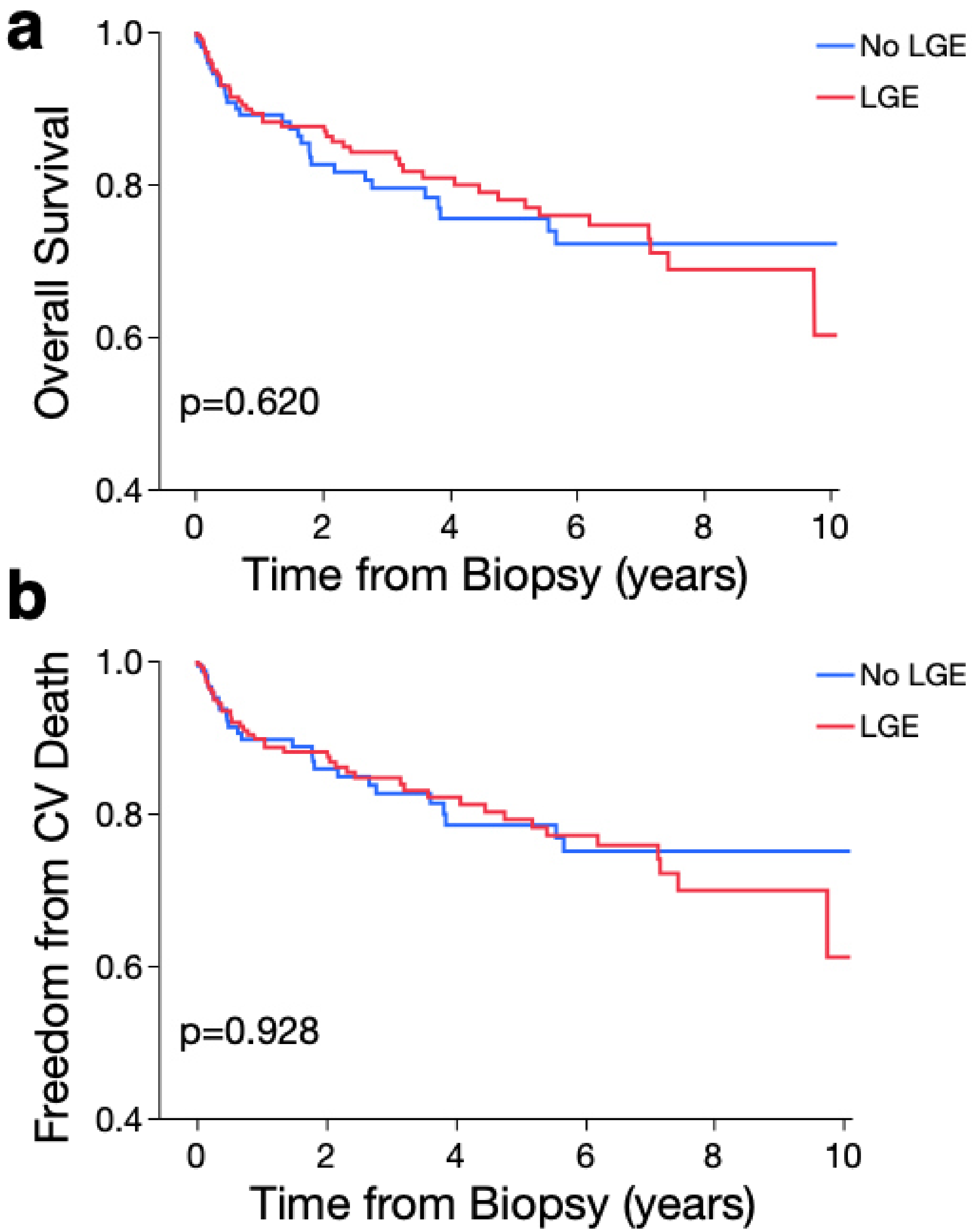

Supplementary Figure S12. Overall and cardiovascular survival analysis based on LGE. Kaplan-Meier curves displaying the adjusted 10-year estimates of **(A)** all-cause mortality and **(B)** cardiovascular (CV) mortality in patients with and without LGE on cardiac MRI. There were no significant ($p<0.05$) differences in survival curves between the distinct subcohorts (n=377).

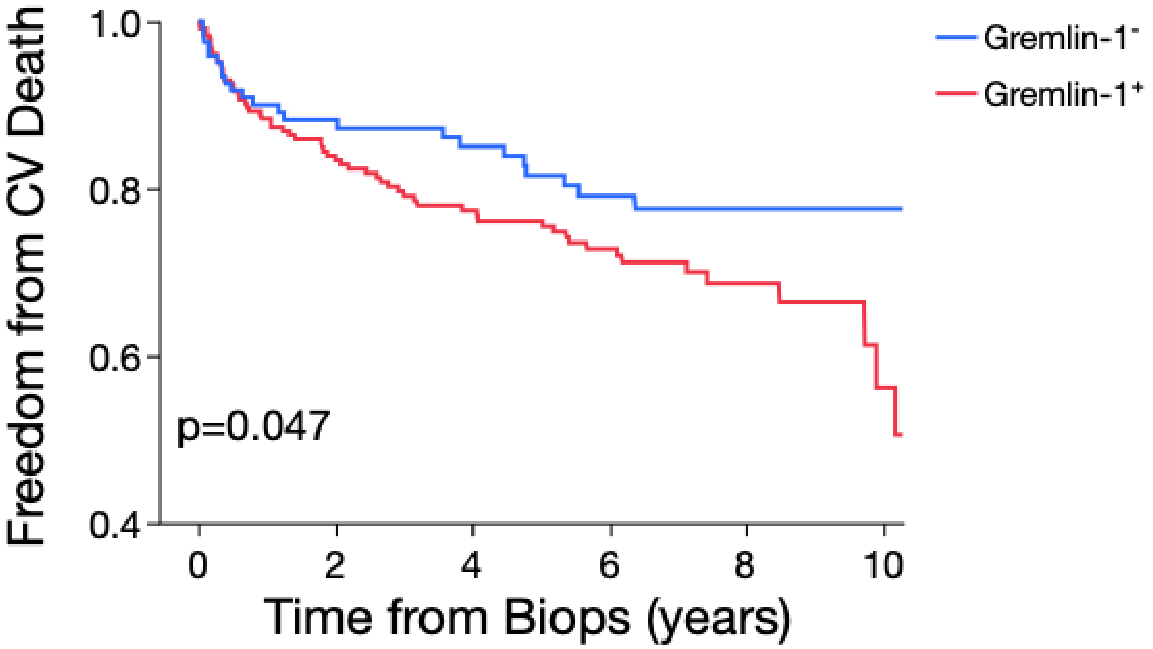

Supplementary Figure S13. Gremlin-1 was significantly associated with enhanced cardiovascular mortality. Kaplan-Meier curves showing transformed 10-year estimates of cardiovascular (CV) mortality in patients with non-ischemic cardiomyopathy. Survival curves exhibit a significantly ($p<0.05$) increased risk of patients with Gremlin-1^{+} EMB (n=360).

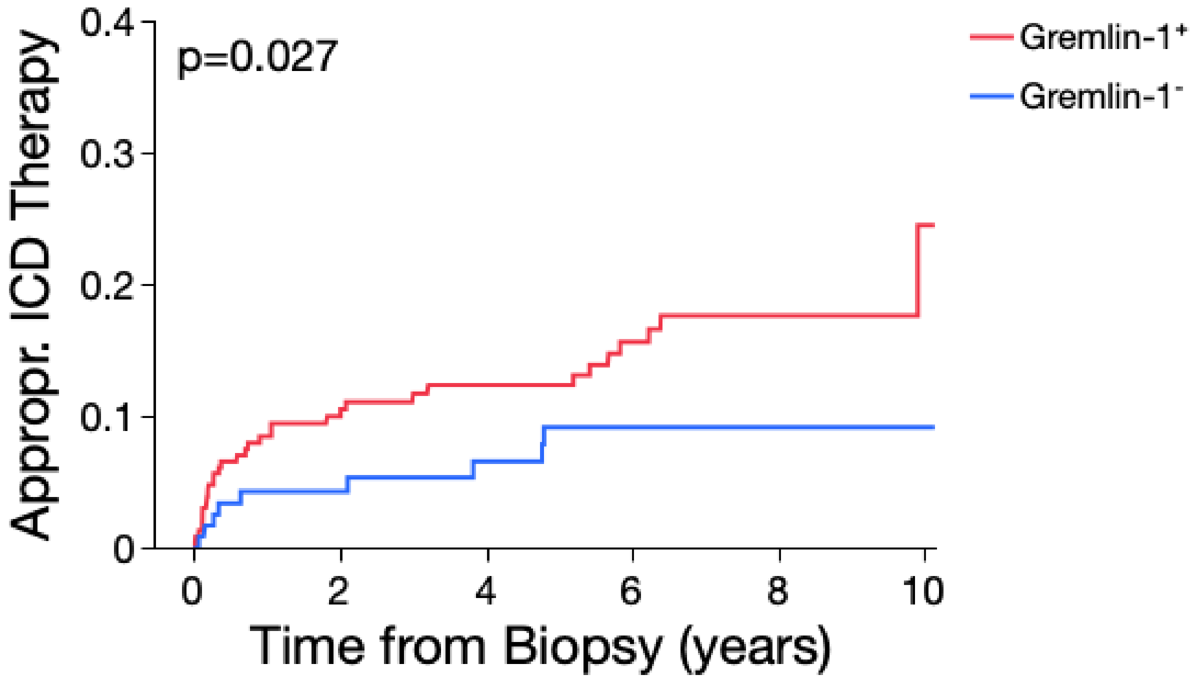

Supplementary Figure S14. Endomyocardial Gremlin-1 was significantly associated with an enhanced risk for appropriate ICD discharge. Kaplan–Meier transformed 10-year estimates for appropriate implantable cardioverter-defibrillator (ICD) therapy. Failure curves show a significantly ($p<0.05$) increased risk of ICD discharge for patients with Gremlin-1^{+} EMB (n=360).

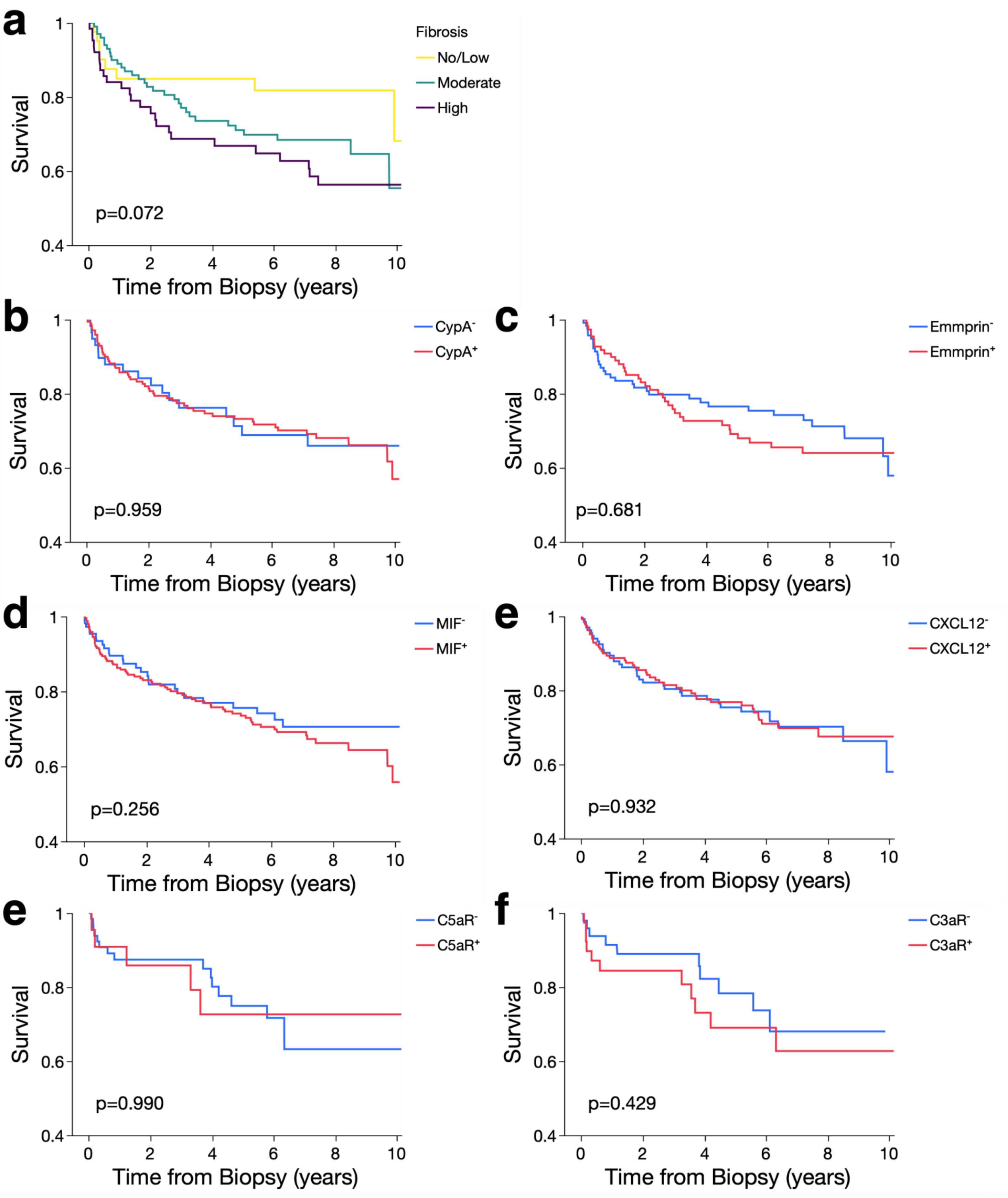

Supplementary Figure S15. Kaplan–Meier transformed 10-year estimates for all-cause mortality. (A-F) Failure curves show cumulative survival rates for patients grouped according to histological findings (e.g., the extend of cardiac fibrosis, the expression of CypA, Emmprin, MIF, CXL12, C5aR, and C3aR).

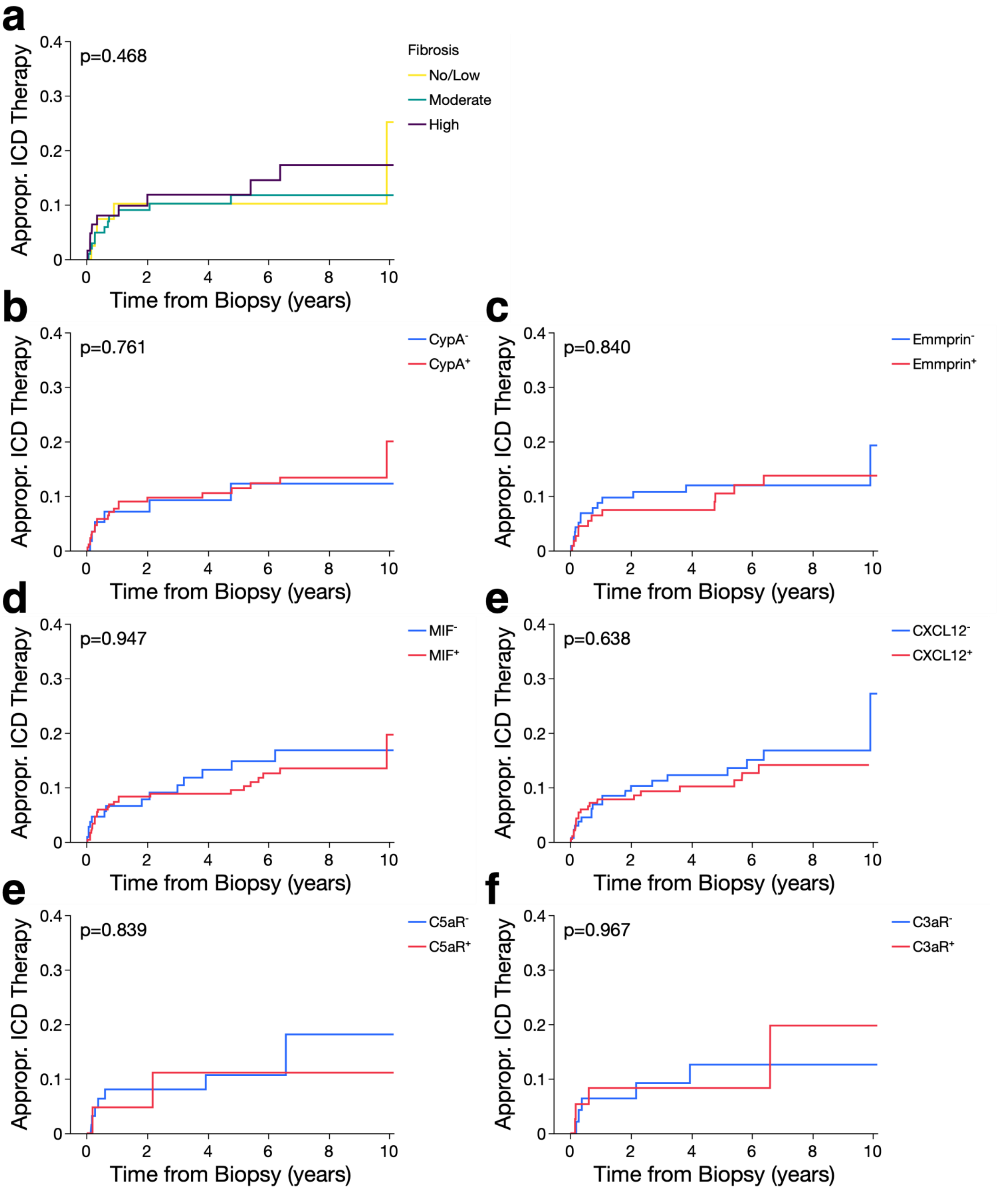

Supplementary Figure S16. **Kaplan–Meier transformed 10-year estimates for appropriate ICD therapy. (A-F)** Failure curves show cumulative event rates for patients grouped according to histological findings (e.g., the extend of cardiac fibrosis, the expression of CypA, Emmprin, MIF, CXL12, C5aR, and C3aR).

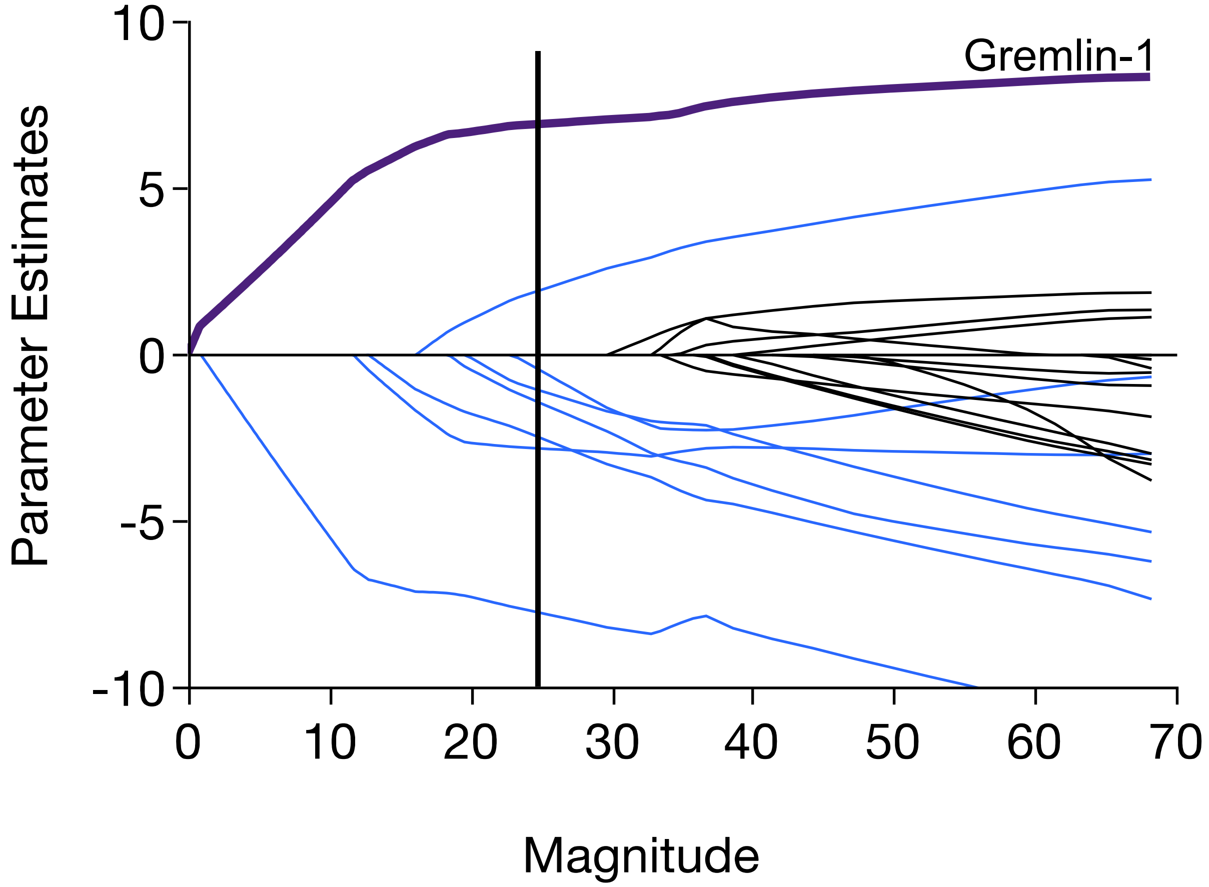

Supplementary Figure S17. Validation of the least absolute shrinkage and selection operator method (LASSO) model for variable selection of histologic data to predict adverse events (death/ICD implantation) in patients with cardiomyopathy. Shrinking of regression coefficients (y-axis) from L1-regularized regression models is plotted against magnitude (x-axis) of variables adjusted for age, gender, and phenotype. Gremlin-1 is highlighted with exhibition of a non-zero coefficient and thus, significantly contributed to 10-year prediction of adverse events in patients with non-ischemic cardiomyopathy.

Supplementary Table S1. Baseline characterization of individuals with non-failing hearts

Variable	Control (n=20)
Female, n (%)	7 (35)
Age, years (mean, 95% CI)	52 (45.4-58.5)
Body mass index (mean, 95% CI)	26.1 (24-28.2)
LVEF (%) (mean, 95% CI)	58.8 (56.1-61.4)

95% CI, confidence interval; LVEF, Left ventricular ejection fraction

Supplementary Table S2. Nominal regression model identified Gremlin-1 as an independent parameter significantly ($p<0.05$) associated with cardiac fibrosis in endomyocardial biopsies of NICM patients (n=210). Significant predictors are highlighted in bold. Two-sided Pearson's chi-squared test.

Multivariable Regression Analysis (p<0.0001)			
Variables	ß Coefficient	Std. Error	p-Value
**Gremlin-1⁺**	1.91	0.30	**<0.0001**
Age	-0.01	0.02	0.535
Male Gender	0.17	0.31	0.583
BMI	0.03	0.06	0.553
Phenotype	0.10	0.62	0.566
ACE inhibitors	0.11	0.32	0.719
AT II receptor antagonists	0.26	0.40	0.527
Aldosterone antagonists	-0.11	0.39	0.786
β-blockers	-0.17	0.30	0.559
Diuretics	-0.05	0.31	0.860

BMI, body mass index; ACE, angiotensin-converting enzyme; AT II, angiotensin II;

Supplementary Table S5. Nominal regression model to identify parameters significantly ($p<0.05$) associated with adverse events including overall mortality and ICD implantation during the 10-year follow-up period. Significant predictors are highlighted in bold. Two-sided Pearson's chi-squared test (n=624).

Multivariable Regression Analysis (p<0.0001)			
Variables	Odds Ratio	95% CI	p-Value
**Gremlin-1⁺**	2.29	1.41-3.72	**<0.001**
**Age**	1.02	0.99-1.03	**0.015**
Female Gender	0.93	0.56-1.54	0.777
BMI	0.99	0.96-1.02	0.343
ACE inhibitors	0.86	0.41-1.81	0.693
AT II receptor antagonists	0.93	0.39-2.19	0.868
Aldosterone antagonists	1.44	0.86-2.44	0.169
β-blockers	1.13	0.55-2.31	0.745
Diuretics	1.60	0.98-1.81	0.062

95% CI, confidence interval; BMI, body mass index; ACE, angiotensin-converting enzyme; AT II, angiotensin II;

Supplementary Table S6. Nominal regression model to identify parameters significantly ($p<0.05$) associated with adverse events including overall mortality and appropriate ICD discharge during the 10-year follow-up period. Significant predictors are highlighted in bold. Two-sided Pearson's chi-squared test (n=624).

**Multivariable Regression Analysis (p=0.005)**			
Variables	Odds Ratio	95% CI	p-Value
**Gremlin-1^{+}**	1.76	1.03-2.98	**0.036**
**Age**	1.02	1.01-1.04	**0.006**
Female Gender	0.76	0.44-1.30	0.315
BMI	0.95	0.91-1.00	0.055
ACE inhibitors	0.80	0.37-1.72	0.567
AT II receptor antagonists	0.90	0.38-2.17	0.817
Aldosterone antagonists	0.85	0.49-1.48	0.576
β-blockers	1.03	0.49-2.20	0.924
Diuretics	1.15	0.67-1.96	0.619

95% CI, confidence interval; BMI, body mass index; ACE, angiotensin-converting enzyme; AT II, angiotensin II;

Supplementary Table S7. List of histological candidate variables for integration in the XGBoost machine learning model to predict the corresponding phenotype in patients with cardiomyopathy.

	**XGBoost Variable**
1.	Complement Component 3 Receptor (C3aR$^{+/-}$)
2.	Complement Component 5 Receptor (C5aR$^{+/-}$)
3.	C-X-C motif chemokine 12 (CXCL12$^{+/-}$)
4.	Ccylophilin A$^{+/-}$
5.	Emmprin+
6.	Gremlin$^{+/-}$
7.	Macrophage Migration Inhibitory Factor (MIF$^{+/-}$)
8.	CD 3$^{+/-}$ T Cells
9.	CD 68$^{+/-}$ Macrophages
10.	Major Histocompatibility Complex (MHC II$^{+/-}$)
11.	Fibrosis$^{low/moderate/high}$
12.	Detection of Viral Genome$^{+/-}$
	Histopathology:
13.	Acute Myocarditis (Symptom Onset <1Month)
14.	Arrhythmia-Induced Cardiomyopathy
15.	Cardiac Amyloidosis
16.	Arrhythmogenic Cardiomyopathy
17.	Autoimmune Disease
18.	Chronic Myocarditis (Symptom Onset ≥1Month)
19.	Dilative Cardiomyopathy
20.	Endomyocardial Fibrosis
21.	Graft Rejection
22.	Cardiomyocyte Hypertrophy
23.	Ischemic Heart Disease
24.	Non-Compaction Cardiomyopathy
25.	Toxic Cardiomyopathy
26.	Unsuspicious

Supplementary Table S8. Specification of the least absolute shrinkage and selection operator method (LASSO) model predicting adverse events (10-year death/ICD implantation) in patients with non-ischemic cardiomyopathy (n=703). Variables with non-zero coefficients were included in the model standardized for age and gender. Gremlin highly significantly contributed to the model (***p<0.001). Significant predictors are highlighted in bold. Two-sided Pearson's chi-squared test.

	**LASSO Regression**	
Variables (Estimate, 95% CI)	Histological Graft Rejection	2.70 (-0.06-5.46)
	**Gremlin*** **	1.50 (0.65-2.35)
	Gender	0.28 (-0.56-1.01)
	Cyclophilin A	0.15 (-0.57-0.88)
	Age	0.03 (0.01-0.05)
	Phenotype MCM	-0.66 (-2.47-1.14)
	Histological Myocarditis	-0.71 (-1.46-0.04)
	Histological Ischemia	-0.94 (-3.22-1.34)
	Phenotype NDLVC	-0.95 (-1.68-(-0.23))
	Histological Amyloidosis	-1.41 (-3.00-0.19)
	**Training Model**	**Validation Model**
AUC (95% CI)	0.80 (0.79-0.81)	0.76 (0.75-0.78)
Sensitivity (95% CI)	0.79 (0.78-0.80)	0.81 (0.79-0.82)
Negative Predictive Value (95% CI)	0.69 (0.67-0.70)	0.67 (0.64-0.69)

Supplementary Table S9. Data availability and missingness across study variables. The table provides the number of patients with available data and missing values for each variable. Differences in sample size reflect real-world data availability, including tissue quality for endomyocardial biopsy analyses and selective use of cardiac imaging.

Variable	Available N	Missing N	% Missing
Age	703	0	0
Sex	703	0	0
NICM phenotype	703	0	0
Cardiopathological analysis	703	0	0
NT-proBNP	202	501	71.3
LVEF (TTE)	606	97	13.8
LVEDD (TTE)	516	187	26.6
LVEF (CMR)	339	364	51.8
LGE (CMR)	373	330	46.9
Edema (CMR)	366	337	47.9
Complement Component 3 Receptor (C3aR)	93	610	86.8
Complement Component 5 Receptor (C5aR)	93	610	86.8
C-X-C motif chemokine 12 (CXCL12)	370	333	47.4
Ccylophilin A	237	466	66.3
Emmprin+	235	468	66.6
Gremlin (EMB)	362	341	48.5
Gremlin (Liquid Biopsy)	301	402	57.2
Macrophage Migration Inhibitory Factor (MIF)	357	346	49.2
CD 3^+ T Cells	632	71	10.1
CD 68^+ Macrophages	633	70	10.0
Major Histocompatibility Complex II (MHC II^+)	633	70	10.0
Fibrosis	210	493	70.1
Viral genome detection	182	521	74.1
mRNA dataset	373	330	46.9
