# Supplementary material for: Endomyocardial Gremlin-1 is associated with structural remodeling and adverse clinical outcomes in non-ischemic cardiomyopathy
Source: Commun Med (Lond). 2026 Jul 2;6:373. doi: 10.1038/s43856-026-01762-9 (PMC13328730; doi:10.1038/s43856-026-01762-9)
Supplement: Supplementary file 3 — Description of Additional Supplementary files [file 43856_2026_1762_MOESM3_ESM.docx]

**Description of Additional Supplementary Files**

File name: Supplementary Data 1

Description: Complete list of 594 analysed genes with the referring Log-transformed fold change between patients with Gremlin+ and Gremlin- tissue sections as well as the referring p-value with correction for multiple hypothesis testing (two-sided ANOVA, BH FDR<5%).

File name: Supplementary Data 2

Description: Complete list of significantly (p<0.05) regulated genes between distinct phenotypes of cardiomyopathies with the referring p-value corrected for multiple hypothesis testing (two-sided ANOVA, BH FDR<5%).

File name: Supplementary Data 3-7

Description: Source data underlying the main figures
