# Supplementary material for: Endomyocardial Gremlin-1 is associated with structural remodeling and adverse clinical outcomes in non-ischemic cardiomyopathy
Source: Commun Med (Lond). 2026 Jul 2;6:373. doi: 10.1038/s43856-026-01762-9 (PMC13328730; doi:10.1038/s43856-026-01762-9)
Supplement: Supplementary file 1 — Transparent Peer Review file [file 43856_2026_1762_MOESM1_ESM.pdf]

nature portfolio

Peer Review File

Endomyocardial Gremlin-1 Is Associated with Structural Remodeling and Adverse Clinical Outcomes in Non-Ischemic Cardiomyopathy

Corresponding Author: Dr Karin Mueller

This file contains all reviewer reports in order by version, followed by all author rebuttals in order by version.

Version 0:

Reviewer comments:

Reviewer #1

(Remarks to the Author)
Harm et al identified gremlin-1 as an independent predictor of fibrosis and clinical endpoints in a heart failure population. The data collected is remarkable and of substantial value. However, the manuscript in its current form is sometimes difficult to follow, as it lacks a clearly defined aim and consistent focus. In addition, the machine learning algorithm should be made publicly available and the novelty of the study remains uncertain, given gremlin-1 is already well described in the context of heart failure. The writing does not sufficiently highlight what is new and innovative.

Major comments:
- The abstract lacks relevant details. For example, it does not present the aim of the study, nor provides methodological details beyond data collection. The results are consequently unclear, making it difficult for the reader to understand how the conclusion is supported.

A more detailed and transparent description of the patient cohort is needed. It is not clear whether the 703 patients are before or after exclusion of CAD patients, and the reasons for lower sample sizes across fibrosis markers, RNA expression, and CMR need to be explained. Furthermore, the terms “HF” and “NICM” appear to be used interchangeably without clarification. A precise definition of terms would improve readability and understanding.

- The manuscript would benefit from a clear overview of the patient cohort used and reasons for lower sample sizes for fibrosis and fibroinflammatory markers, RNA expression, and CMR. The cohort is generally referred to as non-ischemic HF and it is mentioned that patients with CAD are excluded. It is unclear whether the 703 patients are after or before this exclusion. Additionally, the terms HF and NICM appear to have been used interchangeably or the difference is not intuitively clear to me. A proper explanation and definition of the terms would help the reader and increase understanding of the entire manuscript. The main text should adequately describe these details.
- A description of the controls is lacking in the main text, whereas they are depicted in figures. Such relevant information is required in the main text
- Authors mention RNA data, but sporadically specify what type of RNA, which should be clear at every occurrence of the word.
- The aim of the study is insufficiently expressed and the methods and results sections do not appear well aligned. The results section reads as a collection of associations without clear rationale.
- The discussion section is better structured, and the results are integrated more coherently here. However, it would benefit from being placed in a broader scientific context with reference to additional literature and envisioned clinical applications. At present, the discussion largely repeats the results rather than interpreting them in depth.
- The development of a predictive model is of interest, but the methodological details are insufficient, limiting reproducibility. For instance, it is not clear which variables were included in the XGBOOST model. Moreover, the algorithm is not made available, which conflicts with open science practices.
- The correlation with NT-proBNP is mentioned but not described in the methods. Was NT-proBNP included in the machine learning model? Were associations independent of NT-proBNP? Clarification is needed.

Minor comments:

- Introduction line 8-12: these sentences are repetitive.
- Results p. 11, line 14: the period after Subsequently should be replaced for a comma.

Reviewer #2

(Remarks to the Author)
This manuscript presents a thorough and well-executed characterization of multiple histopathological and molecular markers obtained from endomyocardial biopsies (EMBs) in patients with non-ischemic cardiomyopathies (NICM). The integration of clinical follow-up data, immunohistochemistry, and RNA sequencing adds valuable depth to the study and provides a comprehensive view of myocardial remodeling and inflammation. The overall approach is innovative and clinically relevant, and the findings regarding Gremlin-1 as a potential prognostic biomarker are intriguing.
However, several key aspects require clarification or further discussion to strengthen the manuscript and its translational impact.
First, the authors conclude that Gremlin-1 expression in EMBs correlates strongly with adverse outcomes and disease progression in heart failure. While this association is clearly demonstrated, it is not entirely clear what the rationale for emphasizing this invasive biomarker is, given that EMB is not routinely performed in most heart failure cases. The authors should explain more explicitly why a biomarker requiring an invasive procedure would be clinically advantageous, and whether their findings could inform the search for circulating or imaging surrogates of Gremlin-1 that might offer non-invasive diagnostic value. This clarification would substantially enhance the clinical relevance of the study.
Second, the authors report that the degree of fibrosis did not differ significantly among the different NICM phenotypes. This observation is unexpected, considering that various cardiomyopathies (e.g., DCM, RCM, ACM) typically exhibit distinct fibrotic patterns and distribution. Could the authors elaborate on the type and localization of fibrosis observed (interstitial, replacement, perivascular, or endocardial)? Might the lack of difference reflect sampling limitations inherent to EMB?
Third, the detection of viral genomes in 65% of NICM samples appears unusually high. The authors should comment on the methodological details of viral detection (e.g., PCR thresholds, genome copy number, potential contamination) and contextualize these findings relative to previously published epidemiological data on viral persistence in cardiomyopathies. Clarifying whether this rate reflects active infection, latent persistence, or incidental carriage would help readers interpret its biological and clinical implications.
Fourth, regarding late gadolinium enhancement (LGE), the absence of differences between phenotypes is somewhat surprising. In particular, arrhythmogenic (ACM) and non-dilated left ventricular cardiomyopathy (NDLVC) phenotypes are typically associated with more frequent or extensive LGE than hypertrophic cardiomyopathy (HCM). Furthermore, LGE generally correlates with fibrotic burden, yet in this study, Gremlin-1 expression was reduced in patients with LGE. The authors should discuss this apparent paradox—does Gremlin-1 reflect an earlier, pre-fibrotic inflammatory phase rather than established scarring? Or could this discordance result from sampling heterogeneity between MRI-detected fibrosis and biopsy sites?
Fifth, the RNA expression analysis is presented as showing relatively homogeneous transcriptional profiles across phenotypes, with no major differentially expressed genes. This finding warrants further discussion. Were the authors expecting to find transcriptomic signatures specific to distinct phenotypes (e.g., inflammatory vs fibrotic vs arrhythmogenic)? Could the lack of difference be due to the spatial limitation of EMB sampling? A brief discussion on these possible explanations would improve interpretability.
Finally, given the growing relevance of genetic testing in the etiological classification of cardiomyopathies, it would be important to clarify whether genetic analyses were performed in any of the patients.

Reviewer #3

(Remarks to the Author)
There is no doubt about the close relationship between heart failure and inflammation.
In this study, Harm et al. investigated the prognostic value of inflammatory markers from endomyocardial biopsy samples.
This exploratory analysis provides new insight into pathological markers and transcripts involved in heart failure progression.
I would like to congratulate the authors on their efforts; however, I have several concerns regarding the study design.

Major Comments
1. Study endpoint
The primary endpoint of this study is defined as all-cause mortality or ICD implantation.
Risk prediction for sudden cardiac death (SCD) in HCM and DCM is well established, but not in other phenotypes.
I am not sure whether the pathological findings were blinded to the attending physicians; ICD implantation as primary prevention may have introduced intentional bias.
This seems unusual, and the authors should provide a rationale for this point.
Alternatively, please refine the primary endpoint and re-analyze the data.

2. Comparison between phenotypes
The authors analyzed non-ischemic cardiomyopathy and discussed the association between systolic dysfunction and inflammatory status.
However, this dataset consisted of heterogeneous phenotypes — for example, patients with ACM and HCM had normal to mildly reduced systolic function.
It is challenging to discuss LV remodeling and cytokine/chemokine expression within such a heterogeneous entity.
From a molecular biological perspective, DCM and HCM are clearly different diseases, and the mechanisms of adverse

remodeling are substantially distinct
(e.g., myocardial replacement fibrosis in DCM vs. malignant myocardial hypertrophy in HCM).
To address their research question, the authors should focus on a more homogeneous phenotype.

3.Confounding
According to the results, Gremlin-1 might have prognostic potential to predict major adverse cardiac events (MACE).
Gremlin-1 is also an independent predictor of myocardial fibrosis in the multivariable analysis.
Therefore, the prognostic potential of Gremlin-1 could be confounded by myocardial fibrosis, which is already an established marker for poor prognosis and malignant arrhythmias.
It remains unclear whether Gremlin-1 provides any advantage over a simpler and lower-cost fibrotic assessment.

Minor Comments
1. Study population
The authors stated that this study only included patients with new-onset heart failure in the Methods section.
However, in Figure 2b, some cases were diagnosed with graft rejection.
Please clarify the inclusion criteria.
2.XGBoost model
In Figure 3k, the authors stated that the XGBoost model derived from this dataset showed promising predictive value for phenotype classification.
However, this does not represent external validation, and the possibility of overfitting cannot be excluded.
3.Figure 4
>Uniform Manifold Approximation and Projection (UMAP) of RNA data from myocardial biopsy samples unveiled a minor separation between cardiomyopathy phenotypes.
The significance of this “minor separation” is unclear in the manuscript.

>The presence of late gadolinium enhancement (LGE) did not vary significantly among phenotypes (p=0.16) (Figure 4H).
There is no panel labeled “H” in Figure 4.

Version 1:

Reviewer comments:

Reviewer #1

(Remarks to the Author)
I would like to thank the authors for their revisions and for improving the abstract. I still have the following concerns:

- While the abstract has improved, the methods section within the abstract could still be clarified to better reflect the study design and analytical approach.

- The authors state: "We have further explained that variations in sample size across EMB are attributable to differences in data availability and tissue quality. Further, CMR was performed selectively according to clinical indication". However, the manuscript does not sufficiently explain how missing data were handled in the analyses. Please clarify the approach to missing data (e.g., exclusion, imputation, sensitivity analyses) and discuss the potential impact of missingness and bias by indication on the reported results and their interpretation.

-In addition, the rationale underlying the methods remains insufficiently expressed. For each major analysis, please briefly state the specific objective and how it relates to the overall study aims. Including one concise line per analysis to explain its rationale would substantially improve clarity and help the reader understand the logical flow of the study.

- In the rebuttal letter, the authors state: "the primary objective of this study was to investigate whether endomyocardial biopsy–derived histological and molecular features capture disease-relevant pathophysiological mechanisms in non-ischemic cardiomyopathies and can (i) support guideline-based phenotypic classification and (ii) enable clinically meaningful risk stratification.". This formulation is clearer than the current description in the manuscript and would strengthen the introduction if incorporated there.

- Furthermore, it is often difficult to identify precisely what has been changed in the revised manuscript in response to specific comments. It would be helpful if essential textual modifications were quoted directly in the rebuttal letter to facilitate the review process.

Reviewer #2

(Remarks to the Author)
The authors have addressed all major comments from the first review. Gremlin-1 appears to be a promising invasive biomarker for long-term risk stratification and prognosis. However, this reviewer still has concerns regarding the limitations and clinical usefulness of Gremlin-1 as an invasive biomarker in NICM.

The authors did not identify significant differences in Gremlin-1 expression among NICM phenotypes, as it did not distinguish between DCM, NDLC, ACM, etc. In this context, the Abstract should be revised, as it currently claims that Gremlin-1 may "improve phenotypic characterization in patients with NICM."

Differences were observed between NICM (more than 700 EMB samples) and non-failing hearts (only 20 samples). This imbalance should be clearly acknowledged as a limitation of the study, since the control group included only 20 samples compared with more than 700 EMB specimens.

Furthermore, no genetic data were reported, as genetic testing was not performed in all samples. This should also be included as a limitation of the study.

I agree that Gremlin-1 could serve as an "anchor to guide the development of less invasive biomarkers." However, at present, it remains an invasive biomarker, as its assessment requires performing an EMB.

Reviewer #3

(Remarks to the Author)
The authors have addressed many of the previous concerns, and the manuscript has improved substantially after revision.

However, regarding my first comment on the primary endpoint, some uncertainty remains. I understand the authors' rationale that ICD implantation may serve as an indicator of a patient being at high risk of sudden cardiac death (SCD). Nevertheless, the validity of ICD implantation as a component of the primary endpoint is still questionable, as it represents a treatment decision rather than a clinical event.

Essentially, in non-ischemic dilated cardiomyopathy, the merit of ICD implantation as primary prevention remains controversial, and the threshold for implantation varies depending on additional clinical conditions and risk assessment strategies.

In the manuscript, the authors state that "Gremlin-1 was associated with the primary endpoint ($p = 0.02$) based on Kaplan–Meier transformed survival analyses." However, according to the legend, Figures 5e and 5f appear to present Kaplan–Meier curves for mortality only, which suggests that the authors themselves are focusing on death as the most robust outcome.

Would it be possible to perform multivariable analyses using either mortality alone, or a composite of mortality and appropriate ICD therapies, as the endpoint? At the very least, such analyses should be presented in the supplementary material. If the number of events necessitates reducing the number of covariates in the model, an appropriately adapted analysis would still be preferable and would strengthen the robustness of the conclusions.

Version 2:

Reviewer comments:

Reviewer #1

(Remarks to the Author)
The authors have addressed all comments from my review and I have no further comments.

Reviewer #2

(Remarks to the Author)
The authors have satisfactorily addressed all the comments and suggestions raised during the review process. The revised manuscript has been substantially improved in terms of clarity, completeness, and overall quality. The changes implemented have strengthened the work and resolved the concerns identified in the previous version. In my opinion, the manuscript is now suitable for publication in its current form.

Reviewer #3

(Remarks to the Author)
The author appropriately revised the manuscript, improving its clarity and quality.

Open Access This Peer Review File is licensed under a Creative Commons Attribution 4.0 International License, which permits use, sharing, adaptation, distribution and reproduction in any medium or format, as long as you give appropriate credit to the original author(s) and the source, provide a link to the Creative Commons license, and indicate if changes were made.
In cases where reviewers are anonymous, credit should be given to 'Anonymous Referee' and the source.
The images or other third party material in this Peer Review File are included in the article's Creative Commons license, unless indicated otherwise in a credit line to the material. If material is not included in the article's Creative Commons license and your intended use is not permitted by statutory regulation or exceeds the permitted use, you will need to obtain permission directly from the copyright holder.
To view a copy of this license, visit https://creativecommons.org/licenses/by/4.0/

Response to Reviewer #1

Harm et al identified gremlin-1 as an independent predictor of fibrosis and clinical endpoints in a heart failure population. The data collected is remarkable and of substantial value. However, the manuscript in its current form is sometimes difficult to follow, as it lacks a clearly defined aim and consistent focus. In addition, the machine learning algorithm should be made publicly available and the novelty of the study remains uncertain, given gremlin-1 is already well described in the context of heart failure. The writing does not sufficiently highlight what is new and innovative.

Response: We thank the reviewer for the constructive feedback. In response, we have carefully restructured the manuscript to clearly define the study aim and to improve focus and readability throughout the text. To enhance transparency and reproducibility, we have made the machine learning algorithms publicly available and added a corresponding availability statement. Regarding novelty, we agree that this work represents an independent, incremental validation of previous findings. However, our present study substantially extends prior research by providing a comprehensive, guideline-based phenotypic classification of NICM in one of the largest cohorts to date undergoing EMB including a long-term follow-up. We expanded the analysis to include transcriptomic, histological, and CMR data, enabling deeper insight into fibroinflammatory mechanisms associated with disease progression and risk stratification. Ultimately, we critically highlight novel insights by demonstrating its prognostic relevance in a well-characterized NICM cohort and by integrating histological EMB data with machine learning–based phenotyping. These novel aspects are now more clearly emphasized in the revised manuscript.

Major comment of Reviewer #1:

1. The abstract lacks relevant details. For example, it does not present the aim of the study, nor provides methodological details beyond data collection. The results are

consequently unclear, making it difficult for the reader to understand how the conclusion is supported.

Response: We thank the reviewer for this important comment. In response, we have substantially revised the abstract to clearly state the aim of the study, provide key methodological details, and present the main results in a structured and transparent manner. Specifically, the revised abstract now explicitly defines the study objective, outlines the cohort, EMB-based analyses, follow-up, analytical approach, and clearly links the principal findings to the conclusions. We believe these changes improve clarity and allow readers to better understand how the conclusions are supported by the results.

Major comment of Reviewer #1:

2. The manuscript would benefit from a clear overview of the patient cohort used and reasons for lower sample sizes for fibrosis and fibroinflammatory markers, RNA expression, and CMR. The cohort is generally referred to as non-ischemic HF and it is mentioned that patients with CAD are excluded. It is unclear whether the 703 patients are after or before this exclusion. Additionally, the terms HF and NICM appear to have been used interchangeably or the difference is not intuitively clear to me. A proper explanation and definition of the terms would help the reader and increase understanding of the entire manuscript. The main text should adequately describe these details.

Response: We thank the reviewer for this helpful comment and apologize for the imprecise description of the cohort, which may have led to misunderstandings. In accordance with the editor's recommendations, we have moved the supplementary methods section into the main text and clarified that the final cohort of 703 patients exclusively comprises individuals with symptomatic non-ischemic heart failure after exclusion of significant coronary artery disease. We have further explained that variations in sample size across EMB are attributable to differences in data availability and tissue quality. Further, CMR was performed selectively according to clinical indication, primarily in cases where clinical evaluation, echocardiography, and EMB findings were inconclusive for guideline-based classification of NICM. Limited CMR availability further

reflected patient-related contraindications, clinical instable patients, and real-world resource constraints in this all-comers heart failure cohort. These details are now explicitly stated in the revised Methods section. In addition, we have clearly distinguished between heart failure (HF) as the clinical syndrome at presentation and non-ischemic cardiomyopathy (NICM) as the underlying disease entity, with consistent definitions applied throughout the revised manuscript.

Major comment of Reviewer #1:

3. A description of the controls is lacking in the main text, whereas they are depicted in figures. Such relevant information is required in the main text

Response: We thank the reviewer for this important comment. In accordance with the reviewer's and editor's suggestions, we have restructured the methods section of the revised manuscript and now clearly describe the characteristics of the control group in the main text. Here, we state that the investigation of non-failing hearts, post-mortem myocardial samples were obtained from 20 individuals who had died from non-cardiac causes. All individuals had a documented normal left ventricular ejection fraction and no history of cardiovascular disease or treatment with antihypertensive or heart failure medications. Comprehensive review of medical history, clinical records, and pathological examination revealed no evidence of structural heart disease in any donor (**please see supplementary table S1**).

Major comment of Reviewer #1:

4. Authors mention RNA data, but sporadically specify what type of RNA, which should be clear at every occurrence of the word.

Response: We thank the reviewer for this important comment. We have restructured the manuscript and carefully expanded the methods section to describe the analysis of targeted messenger RNA (mRNA). In addition, we have revised the text to explicitly

specify the type of RNA analyzed at all relevant occurrences, thereby improving clarity and avoiding ambiguity.

Major comment of Reviewer #1:

5. The aim of the study is insufficiently expressed and the methods and results sections do not appear well aligned. The results section reads as a collection of associations without clear rationale.

Response: We thank the reviewer for this important comment. In response, and in line with the editor's and other reviewers' suggestions, we have moved the majority of the supplemental methods data to the main text, carefully restructured and streamlined the manuscript to more clearly articulate the overarching aims and to strengthen the alignment between the introduction, methods, and results. As outlined in the revised introduction, the primary objective of this study was to investigate whether endomyocardial biopsy–derived histological and molecular features capture disease-relevant pathophysiological mechanisms in non-ischemic cardiomyopathies and can (i) support guideline-based phenotypic classification and (ii) enable clinically meaningful risk stratification. To address this objective, the results section was reorganized into a multilevel, hypothesis-driven analytical framework, with explicit rationale provided for each analytical step. Specifically, histological and immunohistochemical analyses were used to define phenotype-associated inflammatory and remodeling patterns, integration with mRNA expression profiling identified Gremlin-1 as a central fibroinflammatory determinant, and machine learning and penalized regression approaches were applied to demonstrate the diagnostic and prognostic relevance of EMB-derived features. Through this stepwise integration of histological, molecular, and outcome-based analyses, the results now address the stated aims and highlight the added value of EMB-derived data across multiple biological and clinical levels. We hope that these revisions clarify the study rationale and coherence. Thus, we respectfully hope that the reviewer will find the revised manuscript suitable for acceptance.

Major comment of Reviewer #1:

6. The discussion section is better structured, and the results are integrated more coherently here. However, it would benefit from being placed in a broader scientific context with reference to additional literature and envisioned clinical applications. At present, the discussion largely repeats the results rather than interpreting them in depth.

Response: We thank the reviewer for this constructive comment and recognize the importance of a scientific discussion of our novel results. In response, we have substantially revised and expanded the discussion to place our findings in a broader scientific and translational context. Specifically, we now relate our results to prior work on fibroinflammatory remodeling, established circulating and tissue-based fibrosis biomarkers, and emerging concepts in precision and computational cardiology. We further elaborate on the potential clinical implications of Gremlin-1 as a marker of active myocardial remodeling, particularly in relation to current imaging-based risk stratification strategies and the limitations of conventional parameters such as LGE and LVEF. Importantly, the revised discussion now aims at a more interpretative synthesis, highlighting how EMB-derived Gremlin-1 assessment may complement guideline-based diagnostics, inform personalized risk stratification, and potentially guide future therapeutic strategies. We believe these additions significantly strengthen the conceptual contribution and clinical relevance of the study.

Major comment of Reviewer #1:

7. The development of a predictive model is of interest, but the methodological details are insufficient, limiting reproducibility. For instance, it is not clear which variables were included in the XGBOOST model. Moreover, the algorithm is not made available, which conflicts with open science practices.

Response: We thank the reviewer for highlighting the importance of transparency and reproducibility. The variables included in the XGBoost model were already reported in the

initial version of the manuscript (**please see supplementary table S6**). We fully agree with the reviewer that reproducibility and independent evaluation are essential in machine learning research. In response to the editor's and reviewers' suggestions, we have made the trained XGBoost model publicly available via a persistent repository, with the link (https://doi.org/10.5281/zenodo.18145288) provided in the data availability section.

Major comment of Reviewer #1:

8. The correlation with NT-proBNP is mentioned but not described in the methods. Was NT-proBNP included in the machine learning model? Were associations independent of NT-proBNP? Clarification is needed.

Response: We thank the reviewer for this important comment. NT-proBNP was intentionally not included in the machine learning or penalized regression models, as our objective was to develop a predictive score primarily based on histological EMB-derived parameters adjusted for age, gender, and phenotype. The included variables were reported in the initial version of the manuscript (**please see supplementary table S7**). The analysis of NT-proBNP was performed post hoc and demonstrated a significant correlation between the histological score and clinically relevant NT-proBNP levels, indicating that the EMB-based model closely reflects disease activity at the time of prediction.

Minor comment of Reviewer #1:

- Introduction line 8-12: these sentences are repetitive.

Response: We thank the reviewer for this constructive comment. We have revised the introduction accordingly to improve clarity and conciseness in the indicated section.

Minor comment of Reviewer #1:

- Results p. 11, line 14: the period after Subsequently should be replaced for a comma.

Response: We have corrected the punctuation as suggested.

Response to Reviewer #2

This manuscript presents a thorough and well-executed characterization of multiple histopathological and molecular markers obtained from endomyocardial biopsies (EMBs) in patients with non-ischemic cardiomyopathies (NICM). The integration of clinical follow-up data, immunohistochemistry, and RNA sequencing adds valuable depth to the study and provides a comprehensive view of myocardial remodeling and inflammation. The overall approach is innovative and clinically relevant, and the findings regarding Gremlin-1 as a potential prognostic biomarker are intriguing.
However, several key aspects require clarification or further discussion to strengthen the manuscript and its translational impact.

Response: We thank the reviewer for the thorough evaluation of our manuscript and for the positive assessment of its scientific rigor, innovation, and clinical relevance. We particularly appreciate the recognition of our integrative approach combining histopathological, molecular, and longitudinal clinical data, as well as the interest in Gremlin-1 as a potential prognostic biomarker. We agree that several aspects of the study would benefit from further clarification and discussion. Accordingly, we have carefully revised the manuscript to address these points and provide additional methodological detail, interpretative context, and translational perspective. Below, we respond to each of the reviewer's comments point by point.

Major comment of Reviewer #2:

1. First, the authors conclude that Gremlin-1 expression in EMBs correlates strongly with adverse outcomes and disease progression in heart failure. While this association is clearly demonstrated, it is not entirely clear what the rationale for emphasizing this invasive biomarker is, given that EMB is not routinely performed in most heart failure cases. The authors should explain more explicitly why a biomarker requiring an invasive procedure would be clinically advantageous, and whether their findings could inform the search for circulating or imaging surrogates of Gremlin-1 that might offer non-invasive

diagnostic value. This clarification would substantially enhance the clinical relevance of the study.

Response: We thank the reviewer for this important and insightful comment. We fully acknowledge that the invasive nature of EMB requires a clear clinical and translational rationale for emphasizing EMB-derived biomarkers. In the revised manuscript, we have therefore expanded the discussion to more explicitly justify this approach. First, EMB is an established and guideline-recommended diagnostic tool in selected patients with suspected non-ischemic cardiomyopathy, particularly when inflammatory, infiltrative, or unexplained etiologies are considered. In this clinically relevant setting, our findings demonstrate that Gremlin-1 substantially increases the informational and prognostic yield of EMB by capturing active fibroinflammatory remodeling that is not adequately reflected by conventional clinical parameters or imaging markers. Notably, Gremlin-1 expression was enriched in patients without late gadolinium enhancement, suggesting that it identifies ongoing disease activity at a stage not detectable by structural imaging alone. Second, Gremlin-1 should not be viewed as a screening biomarker for unselected HF populations but rather as a tissue-level reference marker that defines a biologically active disease state. In this context, Gremlin-1 provides mechanistic insight into TGF-β/BMP-driven fibroinflammatory remodeling and helps distinguish active from potentially “burnt-out” disease, thereby addressing a known limitation of current risk stratification strategies. Consistently, machine learning analyses identified Gremlin-1 as one of the most important predictors for phenotype classification and adverse outcomes, underscoring its relevance as a disease-defining marker rather than a purely associative finding. In addition, identification of NICM patients with Gremlin-1^{+} EMB might guide future therapies as the Gremlin1/TGF-ß axis was shown to be treatable in preclinical data.

Finally, we now explicitly discuss that EMB-derived Gremlin-1 assessment may serve as both a mechanistic and prognostic anchor to guide the development and validation of less invasive surrogate markers, including a liquid biopsy or imaging correlates reflecting Gremlin-1–associated pathways. Tissue-level biomarkers have historically played a critical role in calibrating non-invasive diagnostics and enabling precision medicine approaches, and we believe Gremlin-1 fulfills a similar role in NICM. We have

incorporated these clarifications into the revised discussion to strengthen the clinical relevance and translational impact of the study.

Major comment of Reviewer #2:

2. Second, the authors report that the degree of fibrosis did not differ significantly among the different NICM phenotypes. This observation is unexpected, considering that various cardiomyopathies (e.g., DCM, RCM, ACM) typically exhibit distinct fibrotic patterns and distribution. Could the authors elaborate on the type and localization of fibrosis observed (interstitial, replacement, perivascular, or endocardial)? Might the lack of difference reflect sampling limitations inherent to EMB?

Response: We thank the reviewer for this thoughtful and important comment. We agree that different cardiomyopathy phenotypes are typically associated with distinct fibrotic patterns and distributions when assessed at the whole-heart level, but are neither disease-specific nor consistent among distinct phenotypes as previously reported (e.g., doi.org/10.1016/j.tcm.2019.09.003, doi.org/10.1161/circheartfailure.116.003090, doi.org/10.1016/j.jacc.2020.03.019). As outlined in the present study, fibrosis was quantified on EMB specimens using a semiquantitative assessment of the total fibrotic area, without further subclassification, in order to enhance comparability across samples. The absence of significant differences in fibrosis extent across NICM phenotypes therefore likely reflects both the focal nature of fibrosis and, to some extent, inherent sampling limitations of EMB, which captures small regions of the myocardium rather than global or transmural fibrotic patterns. For this reason, we intentionally did not perform a detailed analysis of fibrosis subtypes, as reliable classification of fibrosis patterns requires broader spatial context than EMB. In contrast, imaging modalities such as CMR are better suited to characterize regional or global fibrosis. Accordingly, we performed an additional analysis assessing the distribution and localization of LGE across NICM phenotypes (**please see additional figure 1 to reviewer 2**). While this analysis revealed trends ($p=0.09$) toward phenotype-associated patterns, with a predominance of septal and/or diffuse LGE particularly in patients with DCM and RCM, also no statistically significant

differences were observed across all NICM subgroups. This may in part reflect the inclusion of patients with new-onset HF in this study, who may not yet exhibit the extent of myocardial fibrosis or LGE typically observed in more advanced disease stages. Of note, despite the lack of phenotype-specific differences, fibrosis assessed by EMB was markedly increased compared with non-failing controls and was strongly associated with fibroinflammatory signaling, particularly Gremlin-1 expression, supporting its biological and prognostic relevance at the tissue level. We have expanded the discussion to explicitly acknowledge these sampling and classification limitations and to emphasize that EMB-derived fibrosis reflects local disease activity rather than global fibrotic burden, thereby complementing imaging-based assessments rather than replacing them.

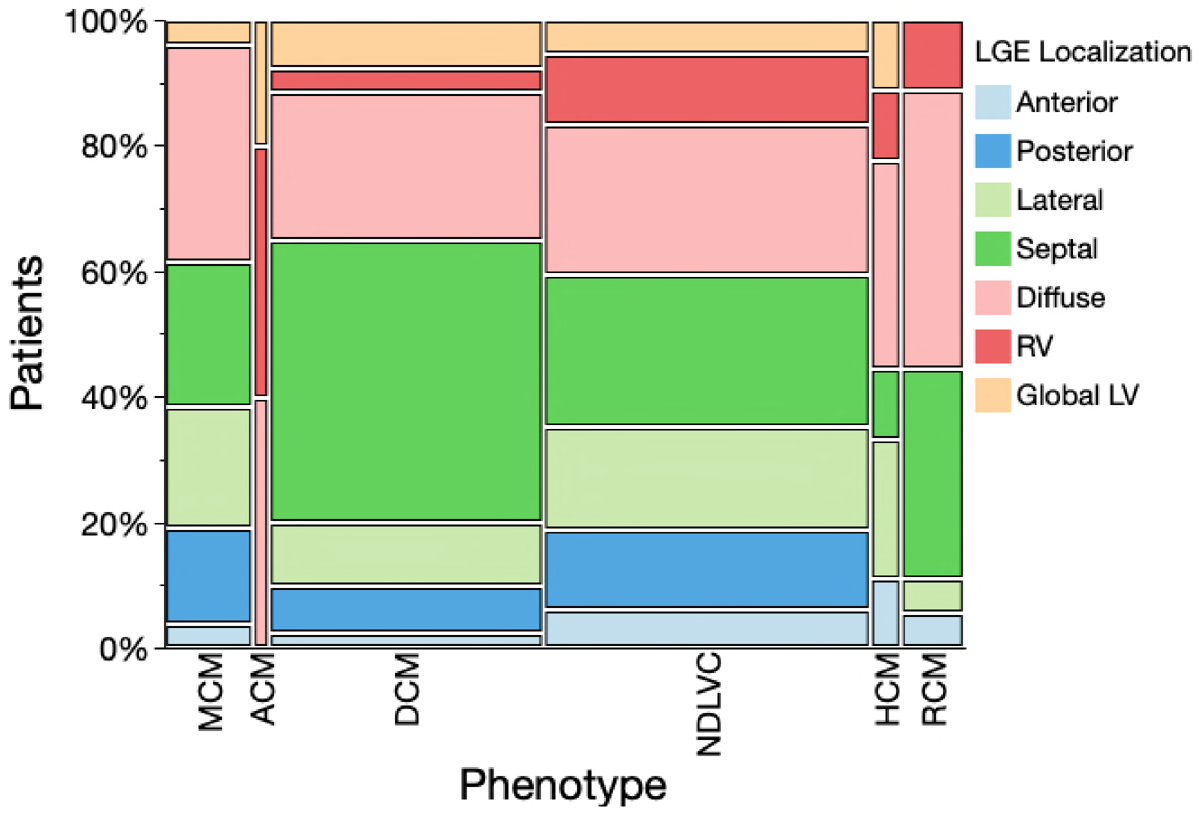

Additional Figure 1 to Reviewer 2. *Mosaic plot illustrating the distribution and localization of late gadolinium enhancement (LGE) patterns across non-ischemic cardiomyopathy phenotypes. No statistically significant differences (Pearson p=0.089) were observed among phenotypes, indicating substantial overlap in LGE distribution in this cohort of patients with new-onset HF.*

Major comment of Reviewer #2:

3. Third, the detection of viral genomes in 65% of NICM samples appears unusually high. The authors should comment on the methodological details of viral detection (e.g., PCR thresholds, genome copy number, potential contamination) and contextualize these findings relative to previously published epidemiological data on viral persistence in cardiomyopathies. Clarifying whether this rate reflects active infection, latent persistence, or incidental carriage would help readers interpret its biological and clinical implications.

Response: We thank the reviewer for this important comment. Viral genomes were detected using established and previously validated nested PCR/RT-PCR protocols applied to deep-frozen or RNAlater-fixed endomyocardial biopsy specimens. Detection included enteroviruses (coxsackie- and echoviruses), parvovirus B19, adenoviruses, Epstein–Barr virus, and human herpesvirus 6. Strict control procedures were applied, including internal controls for nucleic acid integrity (GAPDH), predefined PCR cycling conditions, and confirmation of specificity by automatic DNA sequencing of amplification products, thereby minimizing the risk of contamination or false-positive results. For parvovirus B19, quantitative assessment was available, with the lowest detected viral load being 63 copies/µg myocardial DNA, whereas detection of other viruses was performed qualitatively and classified as positive or negative based on genome presence rather than replication activity. Accordingly, the reported prevalence reflects viral genome detection rather than active infection and is in line with previously published epidemiological data demonstrating frequent viral persistence, particularly of parvovirus B19 and HHV-6, in NICM (https://doi.org/10.1161/01.CIR.0000155616.07901.35). We now explicitly clarify that viral detection in this study was interpreted as latent persistence or prior exposure that may modulate the myocardial inflammatory milieu rather than as evidence of active viral disease, and this clarification has been incorporated into the revised manuscript.

Major comment of Reviewer #2:

4. Fourth, regarding late gadolinium enhancement (LGE), the absence of differences between phenotypes is somewhat surprising. In particular, arrhythmogenic (ACM) and non-dilated left ventricular cardiomyopathy (NDLVC) phenotypes are typically associated with more frequent or extensive LGE than hypertrophic cardiomyopathy (HCM). Furthermore, LGE generally correlates with fibrotic burden, yet in this study, Gremlin-1 expression was reduced in patients with LGE. The authors should discuss this apparent paradox—does Gremlin-1 reflect an earlier, pre-fibrotic inflammatory phase rather than established scarring? Or could this discordance result from sampling heterogeneity between MRI-detected fibrosis and biopsy sites?

Response: We thank the reviewer for this insightful comment. As mentioned in the previous comments, this cohort predominantly included patients with new-onset heart failure, in whom extensive replacement fibrosis detectable by CMR may not yet be established. In line, we show a heterogenous pattern of LGE among phenotypes rather than an absolute difference in the presence of LGE. Notably, the overall extent of fibrosis assessed by EMB was comparable between LGE-positive and LGE-negative patients, indicating that LGE does not necessarily reflect total fibrotic burden at the tissue level (**please see additional figure 2 to reviewer 2**). Thus, the lack of significant differences in LGE across phenotypes likely reflects a combination of disease stage and methodological considerations. The inverse association between LGE and Gremlin-1 expression supports the interpretation that Gremlin-1 preferentially reflects an earlier, biologically active fibroinflammatory phase rather than established replacement fibrosis. This is consistent with our observation that Gremlin-1 is enriched in LGE-negative patients yet strongly associated with fibrosis adverse outcomes, suggesting that histological markers may capture ongoing disease activity before irreversible myocardial scarring becomes apparent on imaging. We carefully discuss this hypothesis including its limitations in the revised manuscript.

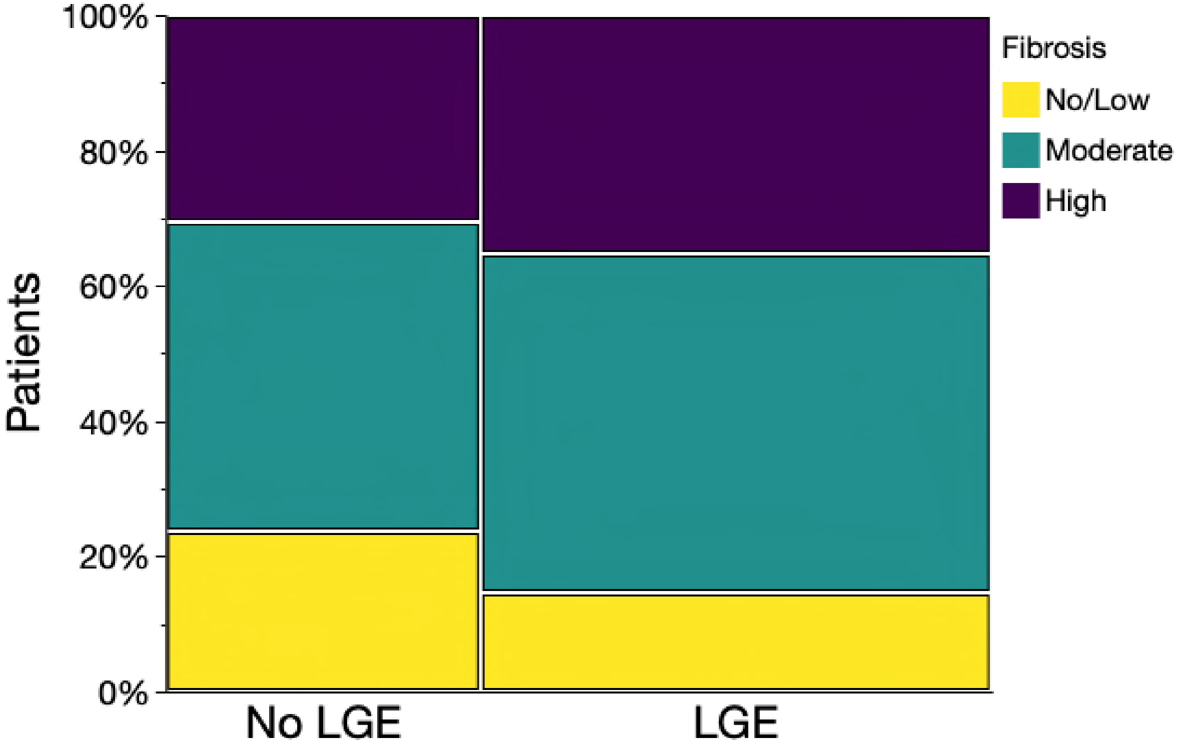

Additional Figure 2 to Reviewer 2. *Distribution of cardiac fibrosis did not differ significantly among patients with LGE on CMR and those without LGE (Pearson p=0.457).*

Major comment of Reviewer #2:

5. Fifth, the RNA expression analysis is presented as showing relatively homogeneous transcriptional profiles across phenotypes, with no major differentially expressed genes. This finding warrants further discussion. Were the authors expecting to find transcriptomic signatures specific to distinct phenotypes (e.g., inflammatory vs fibrotic vs arrhythmogenic)? Could the lack of difference be due to the spatial limitation of EMB sampling? A brief discussion on these possible explanations would improve interpretability.

Response: We agree with the reviewer that this observation warrants further discussion. While we initially hypothesized that distinct NICM phenotypes might exhibit characteristic transcriptomic signatures as shown in previous research (i.e. doi.org/10.1038/s44161-022-00028-6), our UMAP analysis (as shown in **figure 4A)** indicates that mRNA

expression data alone are insufficient to generate a robust molecular fingerprint discriminating NICM phenotypes. This likely reflects both biological overlap in inflammatory and fibrotic pathways across NICM entities and methodological constraints related to the focal nature of EMB sampling. Nevertheless, in-depth analyses revealed phenotype-associated differences in specific regulatory mRNA pathways (as shown in **figure 4B**), indicating that transcriptomic data can capture phenotypic discrepancies of NICM. In the revised discussion subchapter, we have clarified that the mRNA analyses were designed to uncover shared and Gremlin-1–associated disease mechanisms rather than to serve as stand-alone classifiers of NICM phenotypes.

Major comment of Reviewer #2:

6. Finally, given the growing relevance of genetic testing in the etiological classification of cardiomyopathies, it would be important to clarify whether genetic analyses were performed in any of the patients.

Response: We thank the reviewer for highlighting the growing importance of genetic testing in the etiological classification of cardiomyopathies. In the present cohort, genetic analyses were not performed systematically in all patients and were limited to selected cases based on clinical indication. As a result, genetic/sequencing data were not included in the current analyses. We carefully state this in the revised methodological subchapter of our manuscript. We concur with the reviewer that the integration of comprehensive genetic testing with EMB-based phenotyping represents an important perspective for future studies aiming to refine etiological classification and risk stratification in NICM.

Response to Reviewer #3

There is no doubt about the close relationship between heart failure and inflammation. In this study, Harm et al. investigated the prognostic value of inflammatory markers from endomyocardial biopsy samples. This exploratory analysis provides new insight into pathological markers and transcripts involved in heart failure progression.

I would like to congratulate the authors on their efforts; however, I have several concerns regarding the study design.

Response: We sincerely thank the reviewer for the positive and thoughtful assessment of our work. We greatly appreciate the recognition of our novel findings in identifying new markers for improved risk stratification and management of patients with heart failure. We also value the reviewer's constructive comments and in the following we will carefully address the concerns raised regarding the study design.

Major comment of Reviewer #3:

1. Study endpoint: The primary endpoint of this study is defined as all-cause mortality or ICD implantation. Risk prediction for sudden cardiac death (SCD) in HCM and DCM is well established, but not in other phenotypes. I am not sure whether the pathological findings were blinded to the attending physicians; ICD implantation as primary prevention may have introduced intentional bias. This seems unusual, and the authors should provide a rationale for this point. Alternatively, please refine the primary endpoint and re-analyze the data.

Response: We thank the reviewer for this important comment and acknowledge that the endpoint selection needs further clarification. In the present study, our primary objective was to stratify patients with newly diagnosed NICM based on histological parameters derived from EMB, particular focusing on Gremlin-1, in order to identify individuals at increased risk for adverse disease progression and to inform clinically relevant management decisions. Thus, patients were enrolled at the time of new-onset HF and therefore most patients did not have an ICD at study entry. However, this patient population is known to carry a substantial risk for ventricular arrhythmias and SCD during subsequent disease progression. Against this background, we deliberately included ICD as part of the combined primary endpoint to capture clinically important risk escalation and to assess whether EMB-derived markers could predict the need for future device therapy. Importantly, histological findings, including the extent of myocardial fibrosis and Gremlin-1 expression, were assessed blinded to clinical data and were not disclosed to the treating physicians. Consequently, decisions regarding ICD implantation in our cohort followed contemporary guideline-based recommendations and were based on comprehensive clinical risk assessment, including functional status, imaging findings, and additional risk factors, rather than on histopathological findings. In addition, we analyzed secondary endpoints including cardiovascular mortality and appropriate ICD therapy, both of which represent clinically robust outcome measures. Importantly, Gremlin-1 expression consistently predicted these secondary endpoints, confirming its association with arrhythmic risk and adverse cardiovascular outcome beyond the primary composite

endpoint. We agree that ICD implantation should not be interpreted as a surrogate for SCD per se; however, in the context of newly diagnosed NICM, guideline-based ICD implantation reflects an integrated clinical judgment identifying patients at high risk who are likely to benefit from intensified monitoring and device-based therapy. We have clarified this rational in the revised methodological subchapter. Ultimately, we hope that the reviewer will find our endpoint design appropriate to support the use of Gremlin-1 as a marker of disease progression and risk stratification in patients with new-onset NICM across multiple clinically relevant outcomes.

Major comment of Reviewer #3:

2. Comparison between phenotypes: The authors analyzed non-ischemic cardiomyopathy and discussed the association between systolic dysfunction and inflammatory status. However, this dataset consisted of heterogeneous phenotypes — for example, patients with ACM and HCM had normal to mildly reduced systolic function. It is challenging to discuss LV remodeling and cytokine/chemokine expression within such a heterogeneous entity. From a molecular biological perspective, DCM and HCM are clearly different diseases, and the mechanisms of adverse remodeling are substantially distinct (e.g., myocardial replacement fibrosis in DCM vs. malignant myocardial hypertrophy in HCM). To address their research question, the authors should focus on a more homogeneous phenotype.

Response: We fully agree with the reviewer that non-ischemic cardiomyopathy comprises phenotypically heterogeneous entities with substantial differences in their underlying molecular mechanisms and remodeling patterns. Although we concur that etiological drivers differ across NICM phenotypes, progressive disease is often characterized by convergence on shared downstream pathways of inflammation, fibrosis, and maladaptive remodeling, which are highly relevant for the clinical management of HF patients, as demonstrated in this study and previously (e.g. doi.org/10.1016/j.jacc.2020.01.014; doi.org/10.1002/ehf2.14217). Therefore, the primary objective of this study was not to directly or etiologically compare disease-specific remodeling pathways between

individual cardiomyopathy entities, but rather to identify shared, prognostically relevant fibroinflammatory mechanisms and histological markers associated with adverse outcomes across NICM in a large-scale allcomers cohort. In this context, inclusion of heterogeneous phenotypes was indented to reflect real-world clinical practice, particularly in patients presenting with new-onset HF, where etiological classification is often incomplete at initial evaluation, and early risk stratification is of paramount clinical importance. Here, patients were systematically stratified according to guideline-based phenotypes, and phenotypic heterogeneity was accounted for by adjusting survival and predictive analyses accordingly. Notably, phenotype-based classification alone did not discriminate prognosis in our cohort, whereas Gremlin-1 consistently identified patients at risk, supporting its relevance beyond phenotype-specific remodeling, suggesting that it may capture a common pathobiological process related to fibroinflammation and disease progression rather than phenotype-specific remodeling alone. We agree that disease-specific mechanisms, including patterns of fibrosis and myocardial inflammation, differ substantially between cardiomyopathy phenotypes. However, restricting the analysis to a single phenotype would primarily yield phenotype-specific pathophysiological insights while limiting the broader clinical and translational relevance of our findings for clinical practice. We have clarified this rationale in the revised manuscript and explicitly discuss the associated limitations, as well as the need for large, prospective studies focusing on individual phenotypes, which remain challenging to conduct.

Major comment of Reviewer #3:

3. Confounding: According to the results, Gremlin-1 might have prognostic potential to predict major adverse cardiac events (MACE). Gremlin-1 is also an independent predictor of myocardial fibrosis in the multivariable analysis. Therefore, the prognostic potential of Gremlin-1 could be confounded by myocardial fibrosis, which is already an established marker for poor prognosis and malignant arrhythmias. It remains unclear whether Gremlin-1 provides any advantage over a simpler and lower-cost fibrotic assessment.

Response: We thank the reviewer for this important comment regarding the importance of Gremlin-1 and myocardial fibrosis. We fully agree that myocardial fibrosis is an established marker of adverse prognosis and arrhythmic risk in NICM/HF patients.

Of note, in this study, although Gremlin-1 expression was strongly associated with myocardial fibrosis, fibrosis itself did not significantly predict adverse outcomes in survival analyses, whereas Gremlin-1 consistently remained an independent predictor of all-cause mortality, cardiovascular mortality, and appropriate ICD therapy in multivariable models. In addition, LGE as a non-invasive surrogate of myocardial fibrosis, was not predictive of all-cause or cardiovascular mortality in our cohort, whereas Gremlin-1 identified patients at risk even in the absence of LGE. Thus, our findings suggest that the prognostic value of Gremlin-1 cannot be explained by progressive fibrotic burden alone.

From a mechanistic perspective, Gremlin-1 likely reflects active fibroinflammatory signaling as shown by transcriptomic analysis. Whereas histological fibrosis and late LGE primarily reflect cumulative myocardial injury, Gremlin-1 appears to capture ongoing fibroinflammatory disease activity associated with active remodeling and progression, potentially preceding irreversible structural changes in patients with new-onset HF. This distinction may explain its incremental prognostic value over conventional fibrosis-based assessments, particularly in patients with new-onset NICM. Beyond its role as a potential prognostically relevant biomarker, Gremlin-1 is embedded in the TGF-β/BMP signaling axis, which has only recently been shown in preclinical models to be modulated, with inhibition of Gremlin-1 attenuating myocardial inflammation and fibrosis. These experimental data highlight Gremlin-1 as a potential therapeutic target, thereby extending its relevance beyond risk prediction using exclusively fibrosis.

We have clarified these aspects in the revised manuscript and acknowledge that future studies are required to directly compare the incremental prognostic value of Gremlin-1 over established fibrosis markers and to explore its therapeutic potential in prospective, adequately powered cohorts.

Minor comment of Reviewer #3:

1. Study population: The authors stated that this study only included patients with new-onset heart failure in the Methods section. However, in Figure 2b, some cases were diagnosed with graft rejection. Please clarify the inclusion criteria.

Response: We thank the reviewer for raising this point and to clarify the inclusion criteria. The primary inclusion criterion for this study was presentation with new-onset HF, irrespective of the underlying etiology. Thereafter, CAD was ruled-out in all patients enrolled into this prospective study. Thus, patients were therefore enrolled at the time of first clinical presentation with HF, prior to definitive etiological classification. Within this real-world diagnostic framework, subsequent endomyocardial biopsy revealed heterogeneous causes of new-onset heart failure, including non-ischemic cardiomyopathy as well as, in a small subset of patients (n=8), cardiac allograft rejection. These patients had previously been clinically stable, without HF and with normal NT-proBNP levels, but subsequently developed new symptoms and fulfilled diagnostic criteria for HF according to ESC guidelines. Accordingly, they were included because they met the predefined clinical criterion of HF failure at presentation. We have clarified this diagnostic sequence and the inclusion rationale in the revised methods section to avoid misunderstanding.

Minor comment of Reviewer #3:

2. XGBoost model: In Figure 3k, the authors stated that the XGBoost model derived from this dataset showed promising predictive value for phenotype classification. However, this does not represent external validation, and the possibility of overfitting cannot be excluded.

Response: We thank the reviewer for this important comment and agree that the XGBoost model does not represent external validation and that overfitting cannot be fully excluded. Accordingly, the ML analysis was intended as an exploratory, hypothesis-

generating approach rather than a clinically deployable classifier. To reduce overfitting, internal cross-validation and regularization were applied as carefully described in the revised methodological subchapter. In addition, we have clarified this fact in the revised limitations chapter of our manuscript and emphasize that external validation in independent cohorts is required.

Minor comment of Reviewer #3:

3. Figure 4: Uniform Manifold Approximation and Projection (UMAP) of RNA data from myocardial biopsy samples unveiled a minor separation between cardiomyopathy phenotypes. The significance of this “minor separation” is unclear in the manuscript.

Response: We acknowledge that the transcriptomic analyses need further clarification. As stated in the above comment, in the allcomers cohort, we initially hypothesized that distinct NICM phenotypes might exhibit characteristic transcriptomic signatures, as suggested by previous studies (e.g. doi.org/10.1038/s44161-022-00028-6). Therefore, we applied a UMAP analysis (**Figure 4A**) which demonstrates that global mRNA expression patterns alone do not allow to classify NICM phenotypes. Thus, the minor separation reflects subtle transcriptomic differences rather than robust molecular discrimination between phenotypes making it a comparable collective. As mentioned above, it is tempting to speculate that this finding likely results from both biological convergence of inflammatory and fibrotic pathways across NICM entities and methodological limitations related to the focal nature of endomyocardial biopsy sampling. Importantly, subsequent in-depth pathway and regulatory analyses identified phenotype-associated differences in specific mRNA signaling pathways (**Figure 4B**), particularly in Gremlin-1^{+} patients (**Figure 4C-E**), indicating that transcriptomic data can capture biologically meaningful phenotypic variation. Accordingly, we have carefully rephrased the results and discussion to enhance the interpretability of our transcriptomic analyses.

Minor comment of Reviewer #3:

4. The presence of late gadolinium enhancement (LGE) did not vary significantly among phenotypes (p=0.16) (Figure 4H).
There is no panel labeled “H” in Figure 4.

Response: We apologize for the labeling error and thank the reviewer for noting this inconsistency. Revised figure labels have been corrected accordingly in the manuscript referring to **Figure 3H**.

Response to Reviewer #1

I would like to thank the authors for their revisions and for improving the abstract. I still have the following concerns:

1. While the abstract has improved, the methods section within the abstract could still be clarified to better reflect the study design and analytical approach.

Response: We thank the reviewer for this helpful comment. We have further revised the Methods section to more clearly describe the study design and analytical approach, thereby improving clarity and alignment with the overall manuscript.

Abstract / Methods: *"In this prospective cohort study, 703 consecutive patients with symptomatic NICM underwent standardized multimodal evaluation, including clinical assessment, cardiac imaging, and endomyocardial biopsy. Biopsy specimens were analyzed using histology, immunohistochemistry, and targeted myocardial mRNA profiling. Associations between endomyocardial markers, and fibroinflammatory remodeling, imaging parameters, and molecular signatures were assessed cross-sectionally. Long-term prognostic relevance was evaluated using survival and multivariable prediction analyses during follow-up of up to fifteen years for all-cause mortality, cardiovascular mortality, implantable cardioverter-defibrillator (ICD) implantation, and appropriate ICD discharge. "*

Comment of Reviewer #1:

2. The authors state: "We have further explained that variations in sample size across EMB are attributable to differences in data availability and tissue quality. Further, CMR was performed selectively according to clinical indication". However, the manuscript does not sufficiently explain how missing data were handled in the analyses. Please clarify the approach to missing data (e.g., exclusion, imputation, sensitivity analyses) and discuss the potential impact of missingness and bias by indication on the reported results and their interpretation.

Response: In this study, no imputation of missing data was performed to avoid introducing model-based assumptions. Instead, analyses were conducted using an available-case approach, while all patients were retained in the cohort according to the

intention-to-treat principle. The availability of specific data modalities (e.g., immunohistochemistry, mRNA profiling, and CMR) depended on tissue quality, quantity, and clinical indication. Importantly, this reflects the real-world clinical workflow of endomyocardial biopsy–based diagnostics. In routine practice, standardized histological and immunohistochemical assessments (e.g., CD3, CD68, MHCII) are performed first, whereas extended marker panels and molecular analyses are conducted only if sufficient tissue quantity and quality are available. Thus, variation in sample size across analyses arises from predefined technical and clinical constraints rather than selective exclusion. We have clarified this approach in the Methods section and now explicitly report the number of subjects included in each analysis throughout the manuscript and figure legends. In addition, we provide a comprehensive overview of missing data per variable in the Supplementary Material, as requested. Furthermore, we have expanded the Discussion to address the potential impact of missingness. In particular, we acknowledge that incomplete molecular and imaging datasets may affect generalizability. However, the consistency of findings across independent analytical modalities supports the robustness of the overall conclusions.

Methods

"No imputation of missing data was performed to avoid introducing model-based assumptions. All patients were retained in the cohort according to the intention-to-treat principle. Analyses were conducted using an available-case approach, with sample sizes varying according to data availability and tissue quality for specific assessments (e.g., immunohistochemistry, mRNA profiling, and cardiac imaging), reflecting the real-world workflow of endomyocardial biopsy–based diagnostics. The number of patients included in each analysis is reported in ***supplementary table S8*** *and in the corresponding figures and tables.*

A further limitation of this study relates to the incomplete availability of multimodal data across all patients. Not all individuals had complete datasets for histological, immunohistochemical, molecular, and imaging analyses. Missing data primarily resulted from variability in tissue quality and quantity for EMB-based assessments, as well as the clinical applicability of specific diagnostic procedures. Consequently, analyses were performed using available-case data without imputation, which may introduce bias and limit generalizability. Importantly, this reflects the real-world clinical workflow of EMB diagnostics, where core histological assessments are performed routinely, while extended molecular analyses depend on sufficient tissue availability. "

Discussion

“A further limitation of this study is the incomplete availability of multimodal data across all patients. Not all individuals had complete datasets for histological, immunohistochemical, molecular, and imaging analyses. Missing data primarily resulted from variability in tissue quality and quantity for EMB-based assessments, as well as the clinical applicability of specific diagnostic procedures. Consequently, analyses were performed using available-case data without imputation, which may introduce bias and limit generalizability. Importantly, this reflects the real-world clinical workflow of EMB diagnostics, where core histological assessments are performed routinely, while extended molecular analyses depend on sufficient tissue availability. Accordingly, the observed associations should be interpreted in the context of available data and do not exclude the possibility of residual confounding or selection effects. Nevertheless, the consistency of findings across independent analytical modalities supports the robustness of the observed associations, although causal inference cannot be inferred.”

Comment of Reviewer #1:

3. In addition, the rationale underlying the methods remains insufficiently expressed. For each major analysis, please briefly state the specific objective and how it relates to the overall study aims. Including one concise line per analysis to explain its rationale would substantially improve clarity and help the reader understand the logical flow of the study.

Response: We thank the reviewer for this important suggestion. We agree that clearer articulation of the rationale underlying each major analysis improves the readability and logical flow of the manuscript. Accordingly, we have revised the manuscript to include a concise statement of the specific objective at the beginning of each major Results subsection, explicitly linking each analysis to the overall study aims. In addition, we have refined selected Methods subsections to better reflect their conceptual contribution to phenotypic characterization and risk stratification. These changes aim to provide a clearer and more structured narrative throughout the manuscript.

“This study was designed to establish a prospective cohort of patients with symptomatic NICM to enable integrated phenotypic characterization and long-term outcome assessment.”

“NICM phenotypes were defined using a guideline-based multimodal diagnostic framework to provide the clinical reference for subsequent histological and molecular analyses.”

“Phenotypic classification was further refined by integrating clinical, imaging, and histopathological data to link structural characteristics with underlying disease mechanisms.”

“EMB was performed to enable direct assessment of myocardial structure and biology, thereby capturing disease-relevant pathophysiological processes.”

“Immunohistochemical analyses were conducted to quantify myocardial inflammatory and fibroinflammatory markers associated with cardiac remodeling.”

“Targeted myocardial mRNA profiling was performed to characterize molecular pathways underlying fibroinflammatory remodeling linked to adverse outcomes.”

“Viral genome analysis was performed to assess potential infectious contributors to myocardial inflammation and adverse remodeling.”

“Statistical analyses were performed to identify key predictors and develop models supporting phenotypic classification and risk stratification of NICM.”

“To establish a comprehensive baseline of phenotypic and histological characteristics in NICM, we first assessed structural, inflammatory, and immunohistochemical patterns across cardiomyopathy subtypes.”

”To identify key fibroinflammatory drivers of myocardial remodeling beyond phenotypic classification, we next examined the association between histological markers, particularly Gremlin-1, and structural and functional cardiac alterations. ”

”To determine whether EMB-derived histological features can support guideline-based phenotypic classification, we applied machine learning approaches integrating histopathological data.”

”To assess the clinical relevance of Gremlin-1–associated remodeling, we investigated its association with long-term cardiovascular outcomes.”

”Finally, to translate these findings into a clinically applicable framework, we developed and validated a multivariable risk prediction model incorporating EMB-derived features.”

Comment of Reviewer #1:

4. In the rebuttal letter, the authors state: "the primary objective of this study was to investigate whether endomyocardial biopsy–derived histological and molecular features capture disease-relevant pathophysiological mechanisms in non-ischemic cardiomyopathies and can (i) support guideline-based phenotypic classification and (ii)

enable clinically meaningful risk stratification.". This formulation is clearer than the current description in the manuscript and would strengthen the introduction if incorporated there.

Response: We thank the reviewer for this helpful suggestion. We agree that this formulation more clearly reflects the primary objective of the study and have incorporated this statement into the Introduction to improve clarity and alignment with the overall study aims.

Introduction

"In this study, we performed comprehensive risk assessment, including endomyocardial biopsy, in a large prospective cohort of patients with symptomatic heart failure (n=703). We profiled structural, histological, and myocardial mRNA expression changes alongside guideline-based multimodal clinical assessment and cardiac imaging of cardiomyopathies. The primary objective was to determine whether EMB-derived histological and molecular features capture disease-relevant pathophysiological mechanisms in non-ischemic cardiomyopathies and can (i) support guideline-based phenotypic classification and (ii) enable clinically meaningful risk stratification. To this end, we applied machine learning approaches integrating histopathological data with long-term clinical follow-up of up to ten years. We demonstrate that myocardial Gremlin-1 is associated with adverse remodeling and independently predicts adverse cardiovascular outcomes."

Comment of Reviewer #1:

5. Furthermore, it is often difficult to identify precisely what has been changed in the revised manuscript in response to specific comments. It would be helpful if essential textual modifications were quoted directly in the rebuttal letter to facilitate the review process.

Response: We thank the reviewer for this helpful suggestion. To improve clarity and facilitate the review process, we have revised the rebuttal letter to explicitly quote key textual modifications where appropriate. In addition, all changes have been clearly indicated in the marked-up version of the manuscript.

Response to Reviewer #2

The authors have addressed all major comments from the first review. Gremlin-1 appears to be a promising invasive biomarker for long-term risk stratification and prognosis. However, this reviewer still has concerns regarding the limitations and clinical usefulness of Gremlin-1 as an invasive biomarker in NICM.

Response: We thank the reviewer for the positive assessment of our revisions and for recognizing the potential of Gremlin-1 as a prognostic biomarker. We fully agree that the invasive nature of endomyocardial biopsy (EMB) limits its broad applicability in routine clinical practice and represents an important limitation of tissue-based biomarker assessment. Accordingly, we have substantially strengthened the translational perspective of the manuscript by extending the clinical follow-up to 15 years and by adding exploratory liquid biopsy analyses of circulating plasma Gremlin-1 (**please see novel Figure 6**). These additional analyses demonstrate that elevated plasma Gremlin-1 is associated with impaired long-term survival and increased risk of appropriate ICD therapy, while also showing concordance with myocardial Gremlin-1 expression in EMB. Combined tissue and liquid biopsy assessment further revealed stepwise risk stratification, with the highest event rates observed in patients positive for both myocardial and circulating Gremlin-1. We therefore emphasize that Gremlin-1 should not be interpreted solely as an invasive tissue marker, but rather as a biomarker reflecting disease-relevant fibroinflammatory remodeling. Among the assessed tissue-based markers, Gremlin-1 was the only marker consistently associated with adverse clinical outcomes in this cohort. Importantly, the concordance between myocardial and circulating Gremlin-1 supports its role as a mechanistic anchor for the development of less invasive surrogate biomarkers and future serial risk monitoring approaches.

In addition, given its central role in fibroinflammatory signaling pathways, Gremlin-1 may represent a potential therapeutic target in inflammatory heart disease, warranting further experimental and translational investigation. These aspects have now been incorporated into the Discussion to provide a more clinically relevant and forward-looking interpretation of our findings.

Although EMB is inherently invasive and not routinely performed in all patients with suspected NICM, tissue-level assessment remains highly valuable for identifying disease-relevant myocardial remodeling. Importantly, the present extended 15-year follow-up and exploratory liquid biopsy analyses support the concept that Gremlin-1 may serve not only as a tissue-based prognostic marker but also as a mechanistic anchor for the development of less invasive circulating biomarkers.

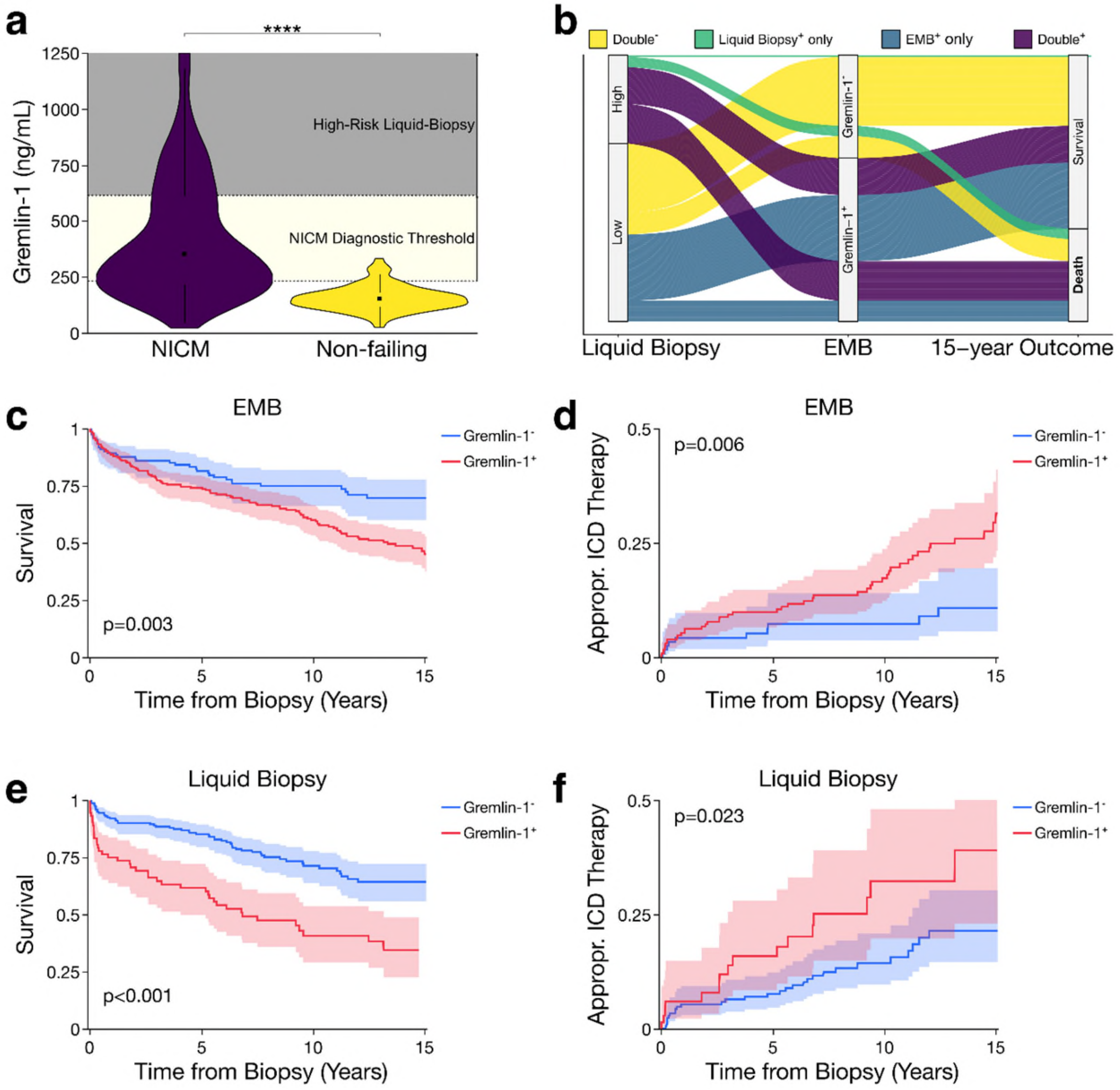

Figure 6. Gremlin-1 bridges tissue pathology and liquid biopsy to improve long-term risk stratification in non-ischemic cardiomyopathy. *(**a**) Circulating plasma Gremlin-1 concentrations in patients with non-ischemic cardiomyopathy (NICM; n=301) and non-failing controls (n=75). Plasma Gremlin-1 was significantly (two-sided Mann Whitney U test) elevated in NICM patients. Receiver operating characteristic (ROC)-derived thresholds identified a NICM diagnostic threshold of 235 ng/l for discrimination of NICM from healthy controls and a separate high-risk liquid biopsy threshold of 614 ng/l for long-term prognostic stratification.* $^{****}p<0.0001$. *(**b**) Combined liquid biopsy and endomyocardial biopsy (EMB) Gremlin-1 status revealed stepwise risk stratification of 15-year mortality. Patients with both elevated plasma Gremlin-1 and Gremlin-1–positive EMB (double positive) showed the highest mortality, whereas double-negative patients demonstrated the most favorable long-term outcome. (**c**) Kaplan–Meier analysis of 15-year all-cause mortality according to myocardial Gremlin-1 expression in EMB. Patients with Gremlin-1*$^{+}$ *EMB showed significantly increased long-term mortality compared with Gremlin-1*$^{-}$ *patients. (**d**) Kaplan–Meier analysis of appropriate ICD therapy according to myocardial Gremlin-1 expression in EMB. Gremlin-1*$^{+}$ *EMB was associated with a significantly increased risk of ventricular arrhythmic events requiring ICD therapy. (**e**) Kaplan–Meier analysis of 15-year all-cause mortality according to circulating plasma Gremlin-1 levels (liquid biopsy). Patients above the predefined high-risk prognostic threshold demonstrated significantly impaired long-term survival. (f) Kaplan–Meier analysis of appropriate ICD therapy according to circulating plasma Gremlin-1 levels. Elevated plasma Gremlin-1 identified patients at significantly increased risk for ventricular arrhythmias and appropriate ICD discharge.*

Comment of Reviewer #2:

The authors did not identify significant differences in Gremlin-1 expression among NICM phenotypes, as it did not distinguish between DCM, NDLC, ACM, etc. In this context, the Abstract should be revised, as it currently claims that Gremlin-1 may "improve phenotypic characterization in patients with NICM."

Response: We thank the reviewer for this important comment. We agree that Gremlin-1 did not distinguish between NICM phenotypes in our study. Accordingly, we have revised the Abstract to remove this statement and now more accurately reflect that Gremlin-1 is associated with myocardial remodeling and adverse clinical outcomes rather than phenotypic classification.

Comment of Reviewer #2:

Differences were observed between NICM (more than 700 EMB samples) and non-failing hearts (only 20 samples). This imbalance should be clearly acknowledged as a limitation of the study, since the control group included only 20 samples compared with more than 700 EMB specimens.

Response: We thank the reviewer for this important comment. We agree that the imbalance between NICM samples and non-failing controls represents a potential limitation of the study and have now explicitly acknowledged this in the discussion.
However, non-failing myocardial samples were obtained post-mortem from carefully selected individuals who had died from non-cardiac causes and had documented normal cardiac function without any history of cardiovascular disease. Comprehensive clinical and pathological evaluation confirmed the absence of structural heart disease in all donors. The limited number of control samples reflects a clinical and ethical constraint, as EMB cannot be obtained from healthy individuals. Despite the smaller control group, we believe that the strict selection criteria ensure an appropriate reference population for comparison.

Although the control group was smaller compared to the NICM cohort, this reflects ethical and practical constraints in obtaining myocardial tissue from truly healthy individuals, and the use of carefully selected post-mortem samples provides a well-defined and clinically appropriate reference despite the imbalance in group sizes.

Comment of Reviewer #2:

Furthermore, no genetic data were reported, as genetic testing was not performed in all samples. This should also be included as a limitation of the study.

Response: We thank the reviewer for this important comment. We agree that the incomplete availability of genetic data represents a limitation of the study. Accordingly, we have revised the manuscript to explicitly acknowledge this point in the discussion, noting that genetic testing was not performed in all patients and may limit the interpretation of associations between geno- and phenotype.

A further limitation of this study is the incomplete availability of multimodal data across all patients. In addition, genetic testing was not performed in all patients, which may limit the interpretation of genotype–phenotype relationships. Not all individuals had complete datasets for histological, immunohistochemical, molecular, and imaging analyses.

Comment of Reviewer #2:

I agree that Gremlin-1 could serve as an “anchor to guide the development of less invasive biomarkers.” However, at present, it remains an invasive biomarker, as its assessment requires performing an EMB.

Response: As mentioned above, our novel data support that Gremlin-1 is not merely a descriptive histological marker, but the only assessed fibroinflammatory marker consistently associated with adverse long-term outcomes, including all-cause mortality, cardiovascular mortality, and appropriate ICD therapy. Importantly, this association remained evident during extended 15-year follow-up, supporting its persistent prognostic relevance beyond conventional structural markers such as fibrosis or LGE. To strengthen the translational applicability, we additionally included exploratory liquid biopsy analyses of circulating plasma Gremlin-1. Plasma Gremlin-1 was significantly elevated in NICM patients, showed concordance with myocardial Gremlin-1 expression, and identified patients with impaired long-term survival and increased arrhythmic risk. Combined tissue and liquid biopsy assessment revealed stepwise risk stratification, with the highest event rates observed in patients positive for both myocardial and circulating Gremlin-1. These findings support the concept that Gremlin-1 may serve not only as a prognostic EMB marker in selected patients, but also as a mechanistic anchor for less invasive screening and future serial risk monitoring using circulating biomarkers. In this context, plasma Gremlin-1 may help identify high-risk patients who could benefit from intensified follow-up, multimodal imaging, or more selective consideration of EMB and device-based therapies. We have incorporated this translational perspective more explicitly into the revised Discussion to provide a clearer and clinically relevant interpretation of our findings.

Response to Reviewer #3

The authors have addressed many of the previous concerns, and the manuscript has improved substantially after revision.

Response: We sincerely thank the reviewer for this positive and encouraging assessment. We greatly appreciate the careful evaluation of our manuscript and are pleased that the revisions have improved its clarity and overall quality.

Comment of Reviewer #3:

However, regarding my first comment on the primary endpoint, some uncertainty remains. I understand the authors' rationale that ICD implantation may serve as an indicator of a patient being at high risk of sudden cardiac death (SCD). Nevertheless, the validity of ICD implantation as a component of the primary endpoint is still questionable, as it represents a treatment decision rather than a clinical event.

Response: We thank the reviewer for this important and thoughtful comment. We agree that ICD implantation represents a treatment decision; however, in the context of non-ischemic cardiomyopathy, it is strongly guided by established guideline criteria and reflects a clinically adjudicated high-risk state for sudden cardiac death rather than an arbitrary intervention. Large RCT and contemporary guideline recommendations consistently demonstrate that ICD therapy reduces sudden cardiac death and is closely linked to disease severity and prognosis, as summarized in recent state-of-the-art literature (e.g., please see doi.org/10.1016/j.jchf.2024.09.014; doi.org/10.1093/eurheartj/ehad194; doi.org/10.1093/eurheartj/ehac262). Moreover, clinical decision-making for primary prevention in NICM already follows structured risk stratification algorithms; however, there remains a clear unmet need for additional biomarkers to further refine individual risk assessment and support clinical decision-making. In this context, our rationale for including ICD implantation in the composite endpoint was to evaluate Gremlin-1 as a potential biomarker that captures clinically relevant risk across the entire clinical pathway, from initial risk stratification and indication

for ICD implantation to the occurrence of arrhythmic events, as reflected by appropriate ICD discharge.

Importantly, to further strengthen the robustness and clinical relevance of our findings, we additionally performed extended 15-year follow-up analyses focusing on hard clinical endpoints, including all-cause mortality and appropriate ICD therapy (**please see novel Figure 6**). Importantly, Gremlin-1 showed consistent associations with both endpoints, with Gremlin-1–positive patients demonstrating significantly increased long-term mortality and a higher incidence of appropriate ICD therapy during extended follow-up. These findings support that the prognostic relevance of Gremlin-1 extends beyond treatment decisions alone and reflects persistent adverse cardiovascular risk. Thus, Gremlin-1 may provide complementary biological information to current clinical criteria and help identify patients at increased risk who could benefit from intensified monitoring and personalized therapeutic strategies.

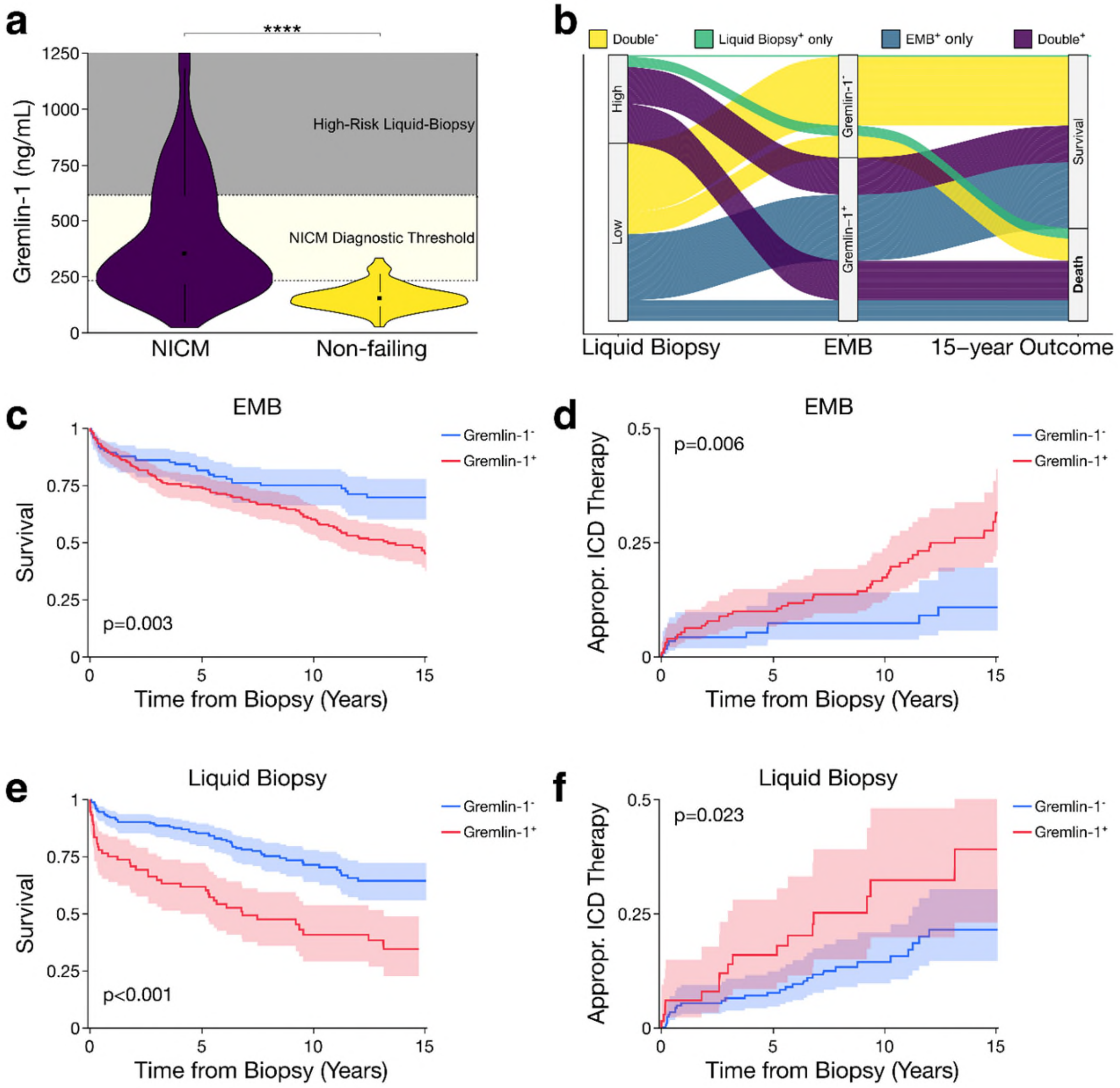

Figure 6. Gremlin-1 bridges tissue pathology and liquid biopsy to improve long-term risk stratification in non-ischemic cardiomyopathy. *(**a**) Circulating plasma Gremlin-1 concentrations in patients with non-ischemic cardiomyopathy (NICM; n=301) and non-failing controls (n=75). Plasma Gremlin-1 was significantly (two-sided Mann Whitney U test) elevated in NICM patients. Receiver operating characteristic (ROC)-derived thresholds identified a NICM diagnostic threshold of 235 ng/l for discrimination of NICM from healthy controls and a separate high-risk liquid biopsy threshold of 614 ng/l for long-term prognostic stratification. ****p<0.0001.(**b**) Combined liquid biopsy and endomyocardial biopsy (EMB) Gremlin-1 status revealed stepwise risk stratification of 15-year mortality. Patients with both elevated plasma Gremlin-1 and Gremlin-1–positive EMB (double positive) showed the highest mortality, whereas double-negative patients demonstrated the most favorable long-term outcome. (**c**) Kaplan–Meier analysis of 15-*

*year all-cause mortality according to myocardial Gremlin-1 expression in EMB. Patients with Gremlin-1⁺ EMB showed significantly increased long-term mortality compared with Gremlin-1⁻ patients. (**d**) Kaplan–Meier analysis of appropriate ICD therapy according to myocardial Gremlin-1 expression in EMB. Gremlin-1⁺ EMB was associated with a significantly increased risk of ventricular arrhythmic events requiring ICD therapy. (**e**) Kaplan–Meier analysis of 15-year all-cause mortality according to circulating plasma Gremlin-1 levels (liquid biopsy). Patients above the predefined high-risk prognostic threshold demonstrated significantly impaired long-term survival. (f) Kaplan–Meier analysis of appropriate ICD therapy according to circulating plasma Gremlin-1 levels. Elevated plasma Gremlin-1 identified patients at significantly increased risk for ventricular arrhythmias and appropriate ICD discharge.*

Comment of Reviewer #3:

Essentially, in non-ischemic dilated cardiomyopathy, the merit of ICD implantation as primary prevention remains controversial, and the threshold for implantation varies depending on additional clinical conditions and risk assessment strategies.

Response: As mentioned above, we agree with the reviewer that the role of ICD implantation for primary prevention in NICM remains subject to ongoing discussion, with indications being closely linked to clinical risk stratification according to current guideline recommendations. Importantly, our study reflects real-world clinical practice, in which ICD implantation was performed based on established risk stratification tools integrating structural abnormalities, symptoms, and additional clinical parameters. However, despite these structured approaches, we acknowledge that current strategies remain limited in their ability to precisely identify patients who will derive the greatest benefit from ICD therapy. In this context, both the early identification of patients at increased risk and the accurate recognition of an elevated risk for SCD are of utmost clinical and prognostic importance. Our approach was therefore designed to complement existing clinical decision-making by evaluating Gremlin-1 in relation to both treatment-related and event-based endpoints along the continuum of care. A biomarker reflecting underlying myocardial disease activity and arrhythmic susceptibility may provide incremental value by refining patient selection, improving timing of intervention, and supporting more individualized management strategies. However, we fully agree that large prospective studies investigating the diagnostic path of ICD implantation are mandatory in this setting.

Nonetheless, we are confident that the consistent association of Gremlin-1 with mortality and appropriate ICD discharge supports its relevance beyond ICD implantation alone.

Comment of Reviewer #3:

In the manuscript, the authors state that "Gremlin-1 was associated with the primary endpoint (p=0.02) based on Kaplan–Meier transformed survival analyses." However, according to the legend, Figures 5e and 5f appear to present Kaplan–Meier curves for mortality only, which suggests that the authors themselves are focusing on death as the most robust outcome.

Response: We thank the reviewer for this careful observation and for highlighting the need for greater clarity. We agree that Figures 5e and 5f display Kaplan–Meier curves for mortality, which we consider the most robust m endpoint. The statement referring to the primary composite endpoint (including all-cause mortality and ICD implantation) is based on additional survival analyses that were not fully illustrated in the main figures. To improve transparency and avoid confusion, we have revised the manuscript to clearly distinguish between analyses of the composite endpoint and those focused on mortality alone. In addition, we have clarified the corresponding figure legends and now explicitly indicate which endpoint is represented in each analysis. Where appropriate, we have also included complementary analyses in the supplementary material to provide a comprehensive overview of the different endpoints assessed.

*Gremlin-1 was associated with all-cause mortality (p=0.02) based on Kaplan-Meier transformed survival analyses. Thus, patients with Gremlin-1^{+} EMB had a significantly (p=0.022) increased 10-year overall mortality rate (48.1%) compared to patients with Gremlin-1^{-} EMB (25.4%) (**Figure 5e**).*

Comment of Reviewer #3:

Would it be possible to perform multivariable analyses using either mortality alone, or a composite of mortality and appropriate ICD therapies, as the endpoint? At the very least, such analyses should be presented in the supplementary material. If the number of events

necessitates reducing the number of covariates in the model, an appropriately adapted analysis would still be preferable and would strengthen the robustness of the conclusions.

Response: We thank the reviewer for this valuable suggestion. Accordingly, we performed additional multivariable analyses using a composite endpoint of mortality and appropriate ICD discharge (please see **novel supplementary figure S6**).

Therefore, we applied the modeling strategy including important baseline characteristics, medication alongside Gremlin-1. Notably, the latter remained independently associated with these endpoints, supporting the robustness of our findings beyond the originally defined composite endpoint. These additional analyses have been included in the revised Supplementary Material and are referenced in the revised manuscript.

Supplementary Table S6. *Nominal regression model to identify parameters significantly (p<0.05) associated with adverse events including overall mortality and appropriate ICD discharge during the ten-year follow-up period. Significant predictors are highlighted in bold. Two-sided Pearson's chi-squared test (n=624).*

Multivariable Regression Analysis (p=0.005)			
Variables	*Odds Ratio*	*95% CI*	*p-Value*
Gremlin-1^{+}	*1.76*	*1.03-2.98*	***0.036***
Age	*1.02*	*1.01-1.04*	***0.006***
Female Gender	*0.76*	*0.44-1.30*	*0.315*
BMI	*0.95*	*0.91-1.00*	*0.055*
ACE inhibitors	*0.80*	*0.37-1.72*	*0.567*
AT II receptor antagonists	*0.90*	*0.38-2.17*	*0.817*
Aldosterone antagonists	*0.85*	*0.49-1.48*	*0.576*
β-blockers	*1.03*	*0.49-2.20*	*0.924*
Diuretics	*1.15*	*0.67-1.96*	*0.619*

95% CI, confidence interval; BMI, body mass index; ACE, angiotensin-converting enzyme; AT II, angiotensin II;
